# What the presence of regulated chemical elements in beached lacustrine plastics can tell us: the case of Swiss lakes

**DOI:** 10.1007/s10661-021-09384-5

**Published:** 2021-10-06

**Authors:** Montserrat Filella, Juan-Carlos Rodríguez-Murillo, Andrew Turner

**Affiliations:** 1 Department F.-A. Forel, University of Geneva Boulevard Carl-Vogt 66, CH-1205 Geneva, Switzerland; 2 Museo Nacional de Ciencias Naturales, CSIC, Serrano 115 dpdo, 28006 Madrid, Spain; 3 School of Geography, Earth and Environmental Sciences, University of Plymouth, Drake Circus, PL4 8AA Plymouth, UK

**Keywords:** Lakes, Plastics, Additives, Antimony, Barium, Bromine, Cadmium, Chromium, Lead, Mercury

## Abstract

Plastics (*n* = 3880) have been sampled from 39 beaches of ten Swiss lakes of varying sizes, hydrodynamics, and catchments, with a selection (*n* = 598) analysed for potentially hazardous (and regulated) chemical elements (As, Ba, Br, Cd, Cr, Hg, Pb, Sb, Se) by X-ray fluorescence spectrometry. Plastic objects and fragments with identifiable or unidentifiable origins were present on all beaches surveyed, and were often most abundant in proximity to major riverine inputs. Chemical elements were detected in between two (Hg) and 340 (Ba) samples with maximum concentrations exceeding 2% by weight for Ba, Cd, Cr, Pb, and Sb. Inter-element relationships and characteristics of the samples suggest that elements are largely present as various additives, including pigments (e.g., Cd_2_SSe, PbCrO_4_), stabilizers (in polyvinyl chloride), and flame retardants (Br). Observations are similar to, and complement, those previously reported in Switzerland’s largest lake (Lake Geneva). Comparison of concentrations of targeted chemical elements in beached plastic with currently used plastics illustrate the interest of these types of measurements in providing an insight into the persistence of plastics in standing stocks and in lakes. This information could help to introduce management schemes that consider whether plastic pollution is new or old and act accordingly.

**Supplementary information:**

The online version contains supplementary material available at 10.1007/s10661-021-09384-5.

## Introduction

Due to their durability and persistence in aquatic environments, plastics have accumulated steadily since first being observed in the 1970s (Carpenter & Smith, 1972; Colton et al., 1974) and have been a ubiquitous contaminant of the whole planet for many years (France, 1992). The impact of plastics in the environment has been the object of enormous scientific study, with more than 3000 papers being published since 1972 according to a Web of Science search conducted in December 2020. However, different environmental compartments and aspects of the problem have received differing degrees of attention.

Thus, with respect to size, more attention has been paid to so-called microplastics than mesoplastics and macroplastics. However, the latter are also of concern because (1) they are a nuisance with effects in tourism and public perception (Beaumont et al., 2019; Krelling et al., 2017), (ii) they are an important source of microplastics through degradation and fragmentation (Barnes et al., 2009; Karlsson et al., 2018; Lambert & Wagner, 2016), and (iii), they have been shown to directly affect biota health through ingestion, entanglement, etc. (Collard et al., 2018; Scherer et al., 2017). Regarding aquatic compartments, there is much less information on plastics in freshwaters than in marine systems. For instance, a review by Blettler et al. (2018) revealed that between January 1980 and May 2018, 13% of studies on microplastics were devoted to freshwaters and 87% to marine waters. With respect to chemical associations, the emphasis of most studies has been on the capacity of plastics, and in particular microplastics, to sorb water-borne organic pollutants and to leach additives such as plasticizers (Hahladakis et al., 2018; Oehlmann et al., 2009). The roles and impacts of chemical elements as potentially hazardous additives have received relatively little scientific consideration with research often centred on trace element adsorption (Turner & Filella, 2021a, 2021b, 2021c).

The present study combines the three aspects described above: freshwaters, potentially hazardous chemical elements, and macroplastics and mesoplastics, by performing X-ray fluorescence (XRF) analysis of beached plastic samples collected from a variety of Swiss lakes. The study continues and complements a previous survey performed in Lake Geneva (Switzerland/France) in 2018 where the presence of potentially hazardous elements was determined in plastic items stranded on 12 beaches (Filella & Turner, 2018) and aims to check if observations in this lake are different to or representative of other lakes of varying physical and catchment characteristics. An additional aim is to evaluate whether the determination of metal contents in plastics might provide information on the age of plastics and their potential accumulation in lakes. Whether surveys of litter stranded on beaches provide a reliable indicator of the abundance of plastic waste in waters is an open question (Ryan et al., 2014, and references therein), but much of what we know about ocean litter is based on surveys of litter stranded on beaches and this is also the case for lakes.

## Materials and methods

### Study sites and sampling

**Fig. 1:**
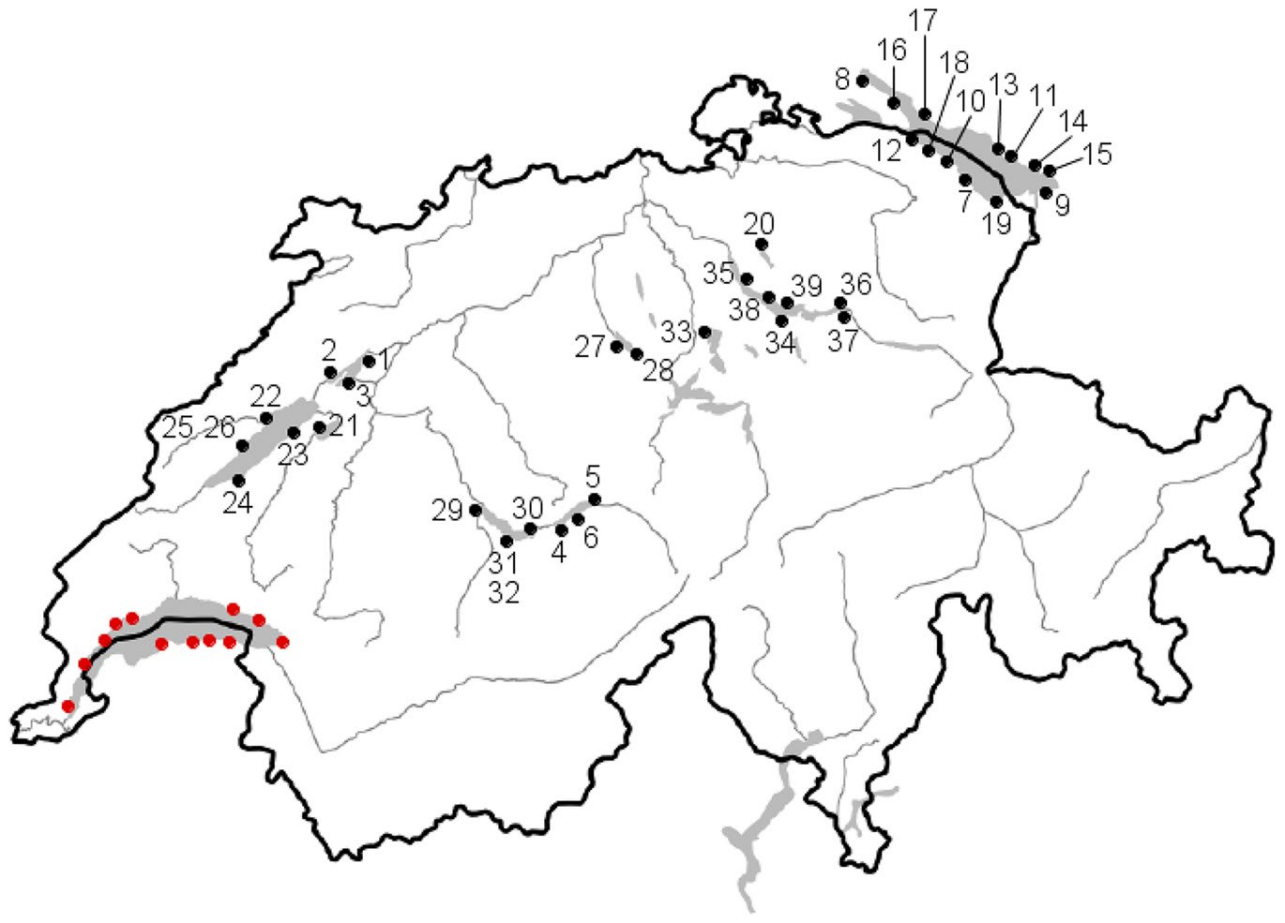
Location of the 39 beaches sampled in the ten Swiss lakes. See Table 2 for the correspondence of numbers. In red, beaches sampled in Lake Geneva in Filella and Turner (2018)

Thirty-nine beaches along the Swiss, German, and Austrian shores of ten Swiss lakes were sampled for the present study. Their locations are shown in Fig. 1, and the main characteristics of the lakes (altitude, catchment area, depth, volume, surface, residence time) are detailed in Table 1. Since lake beaches are used for recreational purposes and some beaches are routinely cleaned, particularly in summer, sampling took place before the Easter holiday break. Most anthropogenic litter observed on each beach was plastic, and all items of this nature retrieved are photographed in the Supporting Information files.

**Table 1:** Lakes studied and their physical characteristics^a^

	Lake	Altitude/m	Catchment area^b^/km^2^	Maximum depth/m	Surface area/km^2^	Volume/km^3^	Residence time/yr	Residents in catchment area^c^
1	Bienne	429	8196	74	37.8	1.12	0.16	1095.5
2	Brienz	564	1108	261	29.8	5.15	2.6	26.1
3	Constance	395	11,461	254	536	48	4.1	455
4	Geneva	372	7419	310	580.1	89	11.3	906.2
5	Greifen	435	156	32	8.5	0.1485	1.2	108.4
6	Morat	429	690	45	22.7	0.531	1.5	87.8
7	Neuchâtel	429	2664	153	242	13.9	8.0	322.6
8	Sempach	504	61	87	14.4	0.639	18.5	15.8
9	Thun	558	2404	217	47.7	6.42	1.9	108.2
10	Zug	414	212	198	38.4	3.2	16.7	102.2
11	Zurich Untersee Zurich Obersee	406 406	1757 1563	136 48	65.1 20.7	3.4 0.47	1.4 0.2	434.1 167.7

^a^Sources: Federal Office for the Environment (www.bafu.admin.ch)

^b^Excluding lake surface

^c^In thousands of inhabitants

Across the entire beach, any item wholly or largely made of plastic that was visible at the surface to the naked eye was retrieved by hand. Samples from each beach were returned to the laboratory in clear, polyethylene zip-lock bags. They were cleared of any visible extraneous material, counted, photographed, and weighed. Table 2 contains information about the total number of items collected, the number analyzed by XRF, and the average item weight for each beach, along with exact locations of the beaches, their lengths, and dates of sampling. Values of item linear densities (items m^−1^), also shown, allow survey results to be compared regardless of the size of the survey. Standardised protocols for litter surveys of marine beaches, such as OSPAR’s (OSPAR, 2010), recommend results to be expressed as items per 100 m or 1 km, but this guideline is linked to the minimum lengths recommended to be sampled (100 m and, if possible, over 1 km in length) which, in our opinion, is not applicable to most lake beaches.

**Table 2:** Name and location of each beach and information about the number (and average weight) of items retrieved and the number of XRF analyses performed

Beach ID	Lake	Beach^a^	Location	Beach length/m	Date	Number of items	Linear density/item m ^−1^	Number of analyses	Mean weight per item/g
1	Bienne	Ipsach	47°07′19″N 7°13′39″E	136	2 March 2019	54	0.40	6	0.67
2		La Neuveville	47°03′36″N 7°05′18″E	25	4 March 2019	18	0.72	0	0.11
3		Lüscherz	47°02′53″N 7°09′05″E	101	2 March 2019	150	1.49	33	1.1
4	Brienz	Böningen, Lütschisand	46°41′26″N 7°53′56″E	130	1 March 2019	44	0.34	7	0.86
5		Brienz	46°44′39″N 8°02′60″E	90	1 March 2019	443	4.92	66	3.0
6		Iseltwald	46°42′41″N 7°57′57″E	25	1 March 2019	20	0.80	5	3.9
7	Constance	Arbon, Strandbad Buchhorn	47°31′36″N 9°25′8″E	350	15 April 2019	55	0.16	14	4.1
8		Bodman G	47°47′20″N 9°03′24″E	1600	16 April 2019	68	0.04	12	2.8
9		Bregenz, Seecamping, A	47°30′25″N 9°42′48″E	90	15 April 2019	372	4.13	40	1.5
10		Kesswil, Badeplatz	47°36′02″N 9°18′54″E	250	15 April 2019	29	0.12	9	3.0
11		Kressbronn G	47°35′13″N 9°35′15″E	300	16 April 2019	156	0.52	43	1.6
12		Kreuzlingen, Seeburgpark	47°39′10 ″N 9°11′8″E	385	15 April 2019	8	0.02	4	13.4
13		Langenargen, Uferpark G	47°35′42″N 9°32′34″E	400	16 April 2019	246	0.62	48	1.3
14		Lindau, Reutiner Bucht G	47°33′2″N 9°42′47″E	30	14 April 2019	396	13.2	52	1.4
15		Lindau, Eichwald Park G	47°32′53″N 9°42′58″E	110	14 April 2019	380	3.45	22	0.93
16		Litzelstetten G	47°42′44″N 9°10′51″E	150	15 April 2019	42	0.28	3	2.4
17		Meersburg G	47°41′56″N 9°15′27″E	450	16 April 209	71	0.16	19	1.1
18		Münsterlingen	47°37′59″N 9°14′29″E	460	15 April 2019	44	0.10	9	1.9
19		Staad, Freibad Speck	47°28′58″N 9°32′25″E	75	15 April 2019	367	4.89	68	2.2
20	Greifen		47°22′00″N 8°40′18″E	50	14 April 2019	8	0.16	1	2.6
21	Morat	Vallamand	46°55′46″N 7°02′48″E	16	3 March 2019	36	2.25	7	3.2
22	Neuchâtel	Bevaix, Pointe du Grain	46°55′33″N 6°50′14″E	350	3 March 2019	54	0.15	7	1.1
23		Gletterens	46°54′30″N 6°55′43″E	107	3 March 2019	17	0.16	4	0.24
24		Ivonand	46°48′20″N 6°44′13″E	310	3 March 2019	60	0.19	13	6.3
25		Vaumarcus A	46°52′38″N 6°45′39″E	20	3 March 2019	10	0.50	4	4
26		Vaumarcus B	46°52′43″N 6°45′41″E	64	3 March 2019	19	0.30	6	3.4
27	Sempach	Nottwill	47°08′29″N 8°08′11″E	27	4 March 2019	48	1.78	3	0.31
28		Sempach, Restaurant Seeland	47°07′33″N 8°11′23″E	40	4 March 2019	44	1.10	10	1.25
29	Thun	Gwatt, Kander delta	46°43′12″N 7°38′22″E	40	2 March 2019	41	1.03	4	0.49
30		Interlaken, Lombach mouth	46°40′47″N 7°48′47″E	240	1 March 2019	84	0.35	20	1.4
31		Leissigen, church	46°39′19″N 7°46′22″E	22	2 March 2019	14	0.64	4	12.7
32		Leissigen, Schreinerei Heinrich	46°39′25″N 7°46′37″E	33	2 March 2019	14	0.42	1	0.43
33	Zug	Hünenberg	47°10′13″N 8°27′12″E	30	4 March 2019	16	0.53	1	0.36
34	Zürich	Au	47°15′07″N 8°38′37″E	600	19 March 2019	88	0.15	10	0.78
35		Küsnacht	47°19′03″N 8°34′36″E	110	18 March 2019	110	1.00	8	0.47
36		Meilen, Strandbad Ländeli	47°15′48″N 8°39′41″E	26	18 March 2019	62	2.38	13	2.1
37		Schmerikon	47°13′29″N 8°56′41″E	33	19 March 2019	80	2.42	11	0.78
38		Schmerikon, Aabach delta	47°13′17″N 8°56′25″E	150	19 March 2019	23	0.15	3	0.48
39		Uerikon, Seebad Risi	47°14′05″N 8°46′12″E	30	18 March 2019	89	2.97	8	0.66

^a^Always in Switzerland except when indicated otherwise. A: Austria, G: Germany

### XRF analysis

Selected samples of plastic (excluding rubbers and foamed materials) of various size, colour, texture, and condition from each location were analyzed by energy-dispersive XRF spectrometry. Multiple samples of relatively new objects known to be free of potentially hazardous elements were not considered (e.g., cotton buds, new plastic caps, food wrapping). Therefore, the percentage of objects where a given element is detected is not necessarily representative of its presence on all retrieved plastic items. Rather, our choice was driven by the objective of the study: to check whether plastics containing potentially hazardous chemical elements are present in lake beaches.

Details of the XRF analysis are given in Turner and Solman (2016) and are summarized as follows. A battery-powered Niton analyzer (model XL3t 950 He GOLDD +) was employed to determine the elements that are deemed to be potentially hazardous according to various EU regulations (European Parliament & Council of the EU, 2009; European Parliament and Council, 2011) and that are reported in our previous study of Lake Geneva (Filella & Turner, 2018): As, Ba, Br, Cd, Cr, Hg, Pb, Sb, and Se, as well as Cl as a proxy for polyvinyl chloride ([Cl] > 150,000 ppm). The instrument was employed in the laboratory in a bench-top accessory stand with the surface of the sample to be probed (usually the thickest and flattest part) suspended centrally over the detector window and 8-mm diameter X-ray source on 3.6 μm polyester film. Measurements were undertaken in a plastics mode coupled with a thickness correction algorithm down to 0.05 mm for a total period of 60 s (30 s each at 50 kV/40 μA and 20 kV/100 μA), and spectra were quantified by fundamental parameters to yield elemental concentrations on a dry weight basis (in ppm) and with a counting error of 2*σ* (95% confidence). A few characteristic spectra are shown for illustration purposes at the end of Supporting Information file 2.

For quality assurance purposes, two Niton reference plastics, certified for either or both of As, Ba, Br, Cd, Cr, Hg, Pb, Sb, and Se (PN 180–619, LOT#T-18 and PN 180–554, batch SN PE-071-N), were analysed at regular intervals, returning concentrations that within 15% of certified values in each case. Under the operating conditions described above, detection limits were generally lowest and below 10 ppm for As, Br, Cr, and Pb and highest and above 70 ppm for Ba, Sb, and Sn. Note that in the case of As, overlap of its Kα fluorescence peak with the Lα peak of Pb, coupled with the relatively low intensity of the As-Kβ line, means that concentrations cannot be effectively calculated where Pb:As exceeds about 10 (Environmental Protection Agency, 2007). Arsenic concentrations reported in this study are, therefore, restricted to those returned by the XRF where Pb was not detected.

## Results and discussion

### Number and type of plastic samples

Overall, 3880 samples were collected, with a range of 8 to 443 on individual beaches, and the total mass of plastic was 6.825 kg, with a mean item weight ranging from 0.11 to 13.4 g for individual beaches. Items could be classified as primary objects that were generally identifiable (e.g., bottle tops, straws, cotton buds, cartridges, clothes pegs, toys, pens, cigarette lighters, cable ties), secondary pieces whose source could be identified (e.g., remains of piping, insulating or adhesive tape, plant pots, sheeting, wrapping, bottles, footwear, rope, twine), secondary pieces that were fragmented beyond recognition, or fragments of expanded plastic that were typically fouled and discoloured (polystyrene, polyurethane).

The number of samples retrieved and the distribution of items in terms of classification and size varied considerably amongst the lakes studied. Although direct beach littering might account for a small number of items sampled (sampling was performed before the more general recreational use of the beaches), visual inspection of the samples (see photographs in Supporting Information files) and their preferential presence along the strandlines suggest that the majority of the beached plastic debris is derived from the water. This was also the case in our previous study of Lake Geneva but differs from observations in other lakes where sampling did not deliberately precede interventions like beach cleans (e.g., Hengstmann & Fischer, 2020; Hoellein et al., 2015).

In oceans, plastics typically enter from land as mismanaged waste transported via rivers or wind (Kershaw & Rochman, 2016; Rech et al., 2014; Schmidt et al., 2017), although local human deposition in coastal areas may also contribute (Hardesty et al., 2017). Given that land-based inputs are likely to be the most significant source of litter in the Swiss lakes rather than from beach visitors, variations in the amount and type of plastic accumulated on beaches might be expected to be attributable to factors such as proximity to inhabited areas and the size and nature of catchments and tributaries. Two cases, with relatively high quantities of plastic located close to river mouths, merit further discussion.

Beach #5 in Lake Brienz (~ 100 m in length), near to the mouth of the Aare River, returned 443 plastic items, many of which were relatively large and appeared to have been broken up recently. For comparison, the linear density of items was 4.92 items m^−1^ in beach #5 and 0.34 and 0.80 items m^−1^ in beaches at Lütschisand (#4), at the mouth of the Lütschine River, and Iseltwald (#6), approximately equidistant from both river mouths, respectively. A detailed map is shown in pages 8 and 9 of the Supporting Information file 1. Given the population upstream of both river mouths is similar and low (below 10,000), the high amount and characteristics of the plastics found in beach #5 suggest a point contamination source. The fact that plastics do not disperse throughout the lake is explained by its morphology and the circulation of Aare River waters once in the lake (Chanudet & Filella, 2008; Wuest et al., 2007).

A completely different situation exists in Lake Constance, where the eastern reaches accumulate all kinds of natural and anthropogenic debris because of the dominant westerly winds and the proximity to the inflow from the Rivers Rhine and Bregenzer Aach (Mürle et al., 2004). The Rhine mouth was diverted several times before 2000 by building an artificial channel into the lake which directs the riverine sediment load to the lake deep basin in order to prevent the silting up of Bregenzer and Fussacher bays (Mürle et al., 2004). However, the light fraction of the river suspended matter, including wood and plastics, is still carried eastwards by winds and surface currents. This is reflected by the highest quantities of plastic samples (~ 400) retrieved from the most easterly or southeasterly beaches at Lindau (#14 and #15), Bregenz (#9), and Staad (#19). The difference of the linear density of items (number of items normalised by beach length) between these four beaches and the rest of beaches in Lake Constance is statistically significant (*p* = 0.007, Mann–Whitney pairwise test). Thereafter, there is a broad reduction in plastic abundance with increasing distance from this region. A detailed map is found in pages 19 and 20 of the Supporting Information file 1.

Because land-based (riverine) sources are prevalent and common to all lakes studied, many of the types of items retrieved from each lake, and described above, are generic. Some objects, however, were present only in a given lake, indicating specific local origins of certain types of plastic waste. For instance, small green or blue pellets used in wastewater treatment plants were mostly found in beaches of Lake Zurich and many black plastic firework casings and other remains of fireworks were extremely abundant on some of the beaches of Lake Constance.

### Occurrence and concentrations of hazardous elements

Table 3 summarizes the detection frequencies and concentrations of hazardous elements in the beached lake plastics analyzed by XRF spectrometry (*n* = 598). Note that data have been combined for all lakes. Also given in Table 3 for comparison are corresponding data for Lake Geneva, Switzerland’s largest lake by surface area and volume, and as published in Filella and Turner (2018).

**Table 3:** A summary of detection frequencies and concentrations for the hazardous elements measured in plastics from the Swiss lakes given in Table 2. The total number of samples analysed was 598, and data from Lake Geneva are shown for comparison

Element	Number (%) of positives	Median/ppm	Minimum/ppm	Maximum/ppm	1Q/ppm	3Q/ppm	Lake Geneva median/ppm^a^
Sb	39 (6.5)	118	30.6	88,400	59.0	171	183
As^b^	10 (1.7)	9.9	3.7	48.3	7.3	14.1	6.3
Ba	340 (48.7)	598	157	144,000	391	1320	670
Br	105 (17.6)	32.4	3.4	7990	17.1	74.4	64.6
Cd	68 (11.4)	493	17.8	20,000	97.3	871	1120
Cr	216 (36.1)	118	17.1	20,900	46.9	687	48.8
Pb	201 (33.6)	192	6.3	35,700	48.3	2960	585
Hg^c^	2 (0.3)	33.5, 73.7					68.6
Se	16 (2.7)	460	141	2020	270	797	394

^a^Median concentration values as reported in Filella and Turner (2018) for 670 samples analysed. Note that the Ba value, not previously published, has been calculated here from the original data (*n* = 243)

^b^Excludes values arising from the spectral interference by lead

^c^The two individual concentrations measured are given rather than the median value

In our survey, only two objects (both retrieved from beach #11, Lake Constance, and indicated in the Supporting Information file 1, page 30) contained detectable Hg. Namely, the remains of a black shoe sole (73.7 ppm) that also contained detectable Pb (290 ppm), Br (63 ppm), and Cr (630 ppm), and a small red fragment (33.5 ppm) that also contained detectable Pb (95.5 ppm). The presence of Hg in the shoe sole may be attributed to a catalytic residue of polyurethane production, but the origin of the Hg in the red fragment is unknown because the absence of Cd rules out a Cd-Hg sulphide-based pigment that was found in several Lake Geneva plastics.

Cadmium has been used as a stabiliser in PVC and as a series of brightly coloured pigments in a variety of plastics. PVC producers and stake-holders voluntarily committed to the phasing out of Cd- (and Pb-) based stabilizers in the EU and, more generally, various regulations limit the amount or deliberate addition of Cd into consumer plastics (Turner, 2019). Despite these restrictions, Cd was detected in 68 samples analysed encompassing all lakes studied, of which 15 were composed of PVC. This may be attributed to the fact that rigid PVC and plastics pigmented with Cd have often been used in products with relatively long lifetimes (e.g., pipes and fittings, hoses, gutters, door and window frames, profiles and plates, roof plates, toys) that continue to contaminate the contemporary waste stream, and the metal readily contaminates plastic recycled from electronic waste (Tamaddon & Hogland, 1993).

**Fig. 2:**
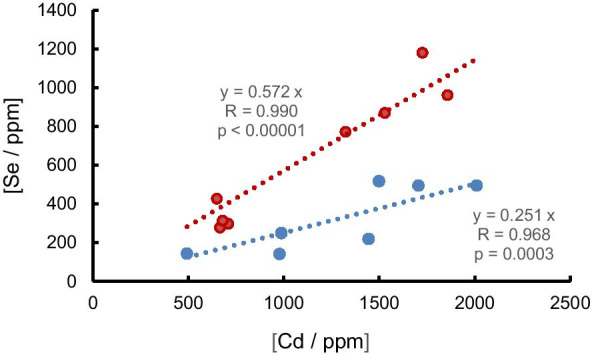
Concentrations of Se versus concentrations of Cd in lake plastic samples. Annotated are equations of best fit for the data split into two groups and defined by red circles (all red in colour) and blue circles (mostly red but also include an orange piece, a blue PVC cable, and a black non-PVC tube). They are given for illustrative purposes

Selenium was detected in 16 samples, two of which were PVC and all of which contained detectable Cd. Selenium is combined with Cd in sulphoselenide pigments whose precise colour (from yellow through to red) can be adjusted by increasing the ratio of Se to S in the solid solution. The Se:Cd ratios in our samples, plotted in Fig. 2, point to the use of at least two different sulphoselenides with mean ratios of about 0.57 and 0.25. The latter ratio is similar to that determined for Se:Cd in plastics sampled from beaches of Lake Geneva (about 0.22; Filella & Turner, 2018).

**Fig. 3:**
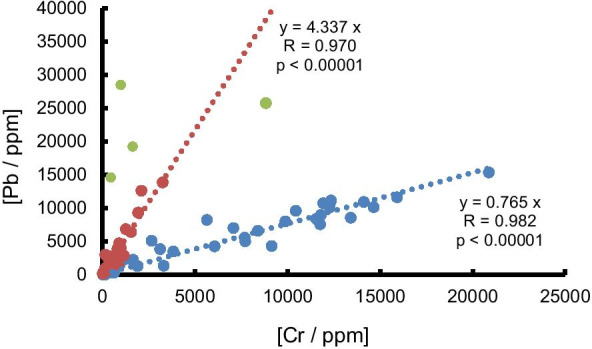
Concentrations of Pb versus concentrations of Cr in lake plastic samples. Data in red and blue are defined by the best-fit lines annotated, while data in green do not conform to either relationship. They are given for illustrative purposes

Like Cd, Pb has been used as a stabiliser in PVC and as a pigment in a variety of plastics and has been subject to similar regulations and voluntary phase-out (Turner & Filella, 2021a, 2021b, 2021c). Lead was detected in a third of the samples analysed (201) across all lakes, including 18 constructed of PVC, with concentrations ranging from about 6 to 35,000 ppm. All but four PVC objects contained Pb concentrations above 1000 ppm. The principal pigments of Pb used in a variety of plastics are based on lead chromate, PbCrO_4_, and the data plotted in Fig. 3 reveal that some of the samples can be defined by a mass ratio of Pb:Cr of about 4, close to the theoretical mass ratio of the metals in lead chromate. Data defined by a line of a smaller slope suggest the use of Cr in additional pigments and additives or its presence as catalytic residue (e.g., chromium oxide). Some black plastic firework casings, extremely abundant in some beaches of Lake Constance, also contained Pb.

Where any potential spectral interferences from Pb could be ruled out, ten samples from seven different beaches (in Lakes Zurich (2), Neuchâtel (2), and Constance (3)) contained detectable As with concentrations in the 3.7 to 16.3 ppm range. Arsenic compounds are known to have been used as antimicrobials in various plastics (Stenmarck et al., 2017) but no particular characteristics of the present objects (e.g., colour, type, usage) enable the origin of As to be ascertained.

Bromine was detected in 105 samples across all lakes, with concentrations ranging from 3.4 to 144,000 ppm. Bromine may be present in some cases as a component of the blue-green pigment, copper phthalocyanine (Lewis, 2004), but is more generally present in plastics because of the addition of brominated flame retardants to electronic plastics and furnishings or to their contamination of plastics more generally through recycling of waste electronic material (Guzzonato et al., 2017). Brominated flame retardants encompass a variety of compounds, some of which, including polybrominated biphenyls (PBB) and polybrominated diphenyl ethers (PBDE), are now restricted. Although XRF spectrometry is unable to discriminate different compounds of the same element, the co-association of Br with Sb, a component of the flame retardant synergist, Sb_2_O_3_, may afford an indirect means of establishing the broad nature of the brominated flame retardant(s) present (Puype et al., 2015). Antimony was detected in 39 samples across the lakes but was only co-associated with Br in six cases and, unlike samples from Lake Geneva, exhibited no distinct relationship with the halogen. The highest Sb concentration (88,400 ppm) occurred in a Br-free sample of grey PVC ribbon from Lüscherz (Lake Bienne) where it was present, presumably, as a flame retardant synergist for the chlorinated polymer. Antimony was more widely detectable at relatively low concentrations (< 500 ppm) and in the absence of Br which might be attributed to residual, catalytic Sb_2_O_3_ used in the production of polyethylene terephthalate (Filella, 2020).

Amongst the elements analysed, Ba was detected in the most cases (340) and across all lakes, with concentrations ranging from 157 to 144,000 ppm. The most commonly employed Ba compound in plastic is BaSO_4_ which acts as an inert, white filler and extender, especially where an increase in specific gravity is required (Gooch, 2011).

## Discussion

### Is the presence of beached plastics in Swiss lakes a surprise?

Obviously, not. Plastics have been reported on lake beaches (Driedger et al., 2015; Mayoma et al., 2019; Vincent & Hoellein, 2017) as a result of usage and subsequent mismanagement and include single-use and multi-use consumer articles and recognisable and unidentifiable pieces, fragments, and films. Indeed, according to Geyer et al. (2017), 8300 million metric tonnes of polymer resins, synthetic fibres, and additives had been globally produced up to 2015, of which 79% had accumulated in landfills or had been mismanaged and ended up in the environment. In the case of Switzerland, a probabilistic material flow analysis applied to seven polymers estimated a combined emission to surface waters in Switzerland of 109 ± 40 t of macroplastics and 15 ± 9 t of microplastics in 2014 (Kawecki & Nowack, 2019). Thus, the presence of a range of plastics on Swiss lake beaches is not unexpected.

### Can we establish the origins of beached plastics?

A detailed analysis and rigorous source appointment of plastic debris in all lakes studied require extensive temporal and spatial sampling campaigns, such as the national one currently in place for litter in Swiss freshwater systems (https://hammerdirt-analyst.github.io/IQAASL-End-0f-Sampling-2021/end_of_sampling_iqaasl/eos_asa.html/). For instance, this campaign has performed 386 surveys from 143 locations from April 2020 to May 2021 by using a modified OSPAR/Marine litter watch protocol and 54’744 objects have been collected. The influence of indicators such as population, surface area uses (e.g., buildings, woods, outdoor activities, agriculture, length of all roads and trails), number of river discharge intersections, etc. has been evaluated. Establishing such, or other, correlations with our data was not the objective of this study and, obviously, would have no statistical meaning with our limited number of samples and sample campaigns. It is clear, however, that the two cases discussed above (Lake Brienz and Lake Constance) confirm that rivers play a key role in ‘feeding’ lakes with plastics.

### What measuring some chemical element contents in plastics tell us

First, the presence of potentially hazardous elements in the mismanaged plastics is always of concern even if it is unclear whether they imply any serious ecotoxicological impact. A few studies that have been performed using unrealistically high concentrations of microplastics—a common trait in (eco)toxicity studies involving such particulates (Lenz et al., 2016; Phuong et al., 2016)—suggest toxic effects arising from metals as additives or having been acquired (adsorbed) from the environment (Lu et al., 2016; Boyle et al., 2020) but, in practice, it remains an unexplored question. Secondly, in some cases, the presence of certain chemical elements can also point to local and contemporary noxious sources. This is the case, for instance, of Pb-rich black plastic firework casings abundant in some beaches of Lake Constance.

However, the determination of the concentration of some targeted elements in beached plastics as a tool to understand the cycling of plastic litter has a utility well beyond possible ecotoxicological considerations or spotting problematic local sources. Plastics have been accumulating for several decades either in the lakes or in the standing stock and knowing the lifespan of plastic debris and their environmental fate are needed to introduce adequate management schemes. Plastic debris age might be deduced visually from their condition, but this approach provides very limited qualitative information. The degradation of plastics in the environment can be followed by using spectroscopic or microscopic techniques (Brandon et al., 2016; Ioakeimidis et al., 2016), but they only provide information about polymer degradation. This can be due to many factors, and degradation is not easy to link to the persistence of the plastic in the system. It is here where detecting the presence of some chemical elements in the plastics (e.g., As, Cd, Hg, Pb) provide a useful, alternative tool. In our initial study in Lake Geneva (Filella & Turner, 2018), we detected the presence of restricted additives, some of which, such as Hg, had been phased out many decades ago. Potentially hazardous chemical elements found in this study seem to be largely absent in common plastic consumer products in Switzerland (which is expected in accordance with current regulations), except in a very few recycled objects (Cd and Pb, with As and Hg entirely absent) (Filella, 2020). This points to long residence times of these items in lakes that considerably exceed hydraulic residence times.

A second group of chemical elements that might provide useful information is those present in additives that have an impact on the physical properties of plastics, and in particular on density. This effect has been exploited in the recycling industry in order to separate harmful and recyclable plastics (for example, Br-free and Br-positive materials; Retegan et al., 2010) and was recently addressed in terms of natural plastic fractionation in the marine environment (Turner & Filella, 2020; 2021a). Significantly, many polymers, including the polyolefins, have a density that is marginally below that of temperate freshwater (1.0 g mL^−1^) and the presence of moderate quantities of relatively dense additives, like BaSO_4_ (4.5 g mL^−1^) or PbCrO_4_ (6.3 g mL^−1^), may incur a positive settling velocity to the material and increase its retention time in lakes. Our median value for Ba (598 ppm) (and the corresponding value in Lake Leman, 670 ppm) is significantly different from the median value of 1540 ppm for consumer products reported in Turner and Filella (2020). Although this could suggest a fractionation of plastics, with ‘heavier’ ones remaining in lakes, this observation needs to be considered with caution because the choice of objects to analyse in this study was focused on objects prone to contain potentially hazardous elements. Since Ba is present in many ‘innocuous’ plastics, a sound conclusion would require the analysis of all objects collected.

In conclusion, the observations of the present study, coupled with their similarities with findings previously reported in Lake Geneva (median elemental concentrations not statistically different, *p* = 0.25, Wilcoxon two-sample paired test) and the lack of hazardous elements in current consumer plastics, illustrate how the measurement of these chemicals in lake beached plastics provides a powerful tool to understand plastic cycling in catchments of varying hydrodynamic and geomorphological characteristics. After this proof-of-concept exercise, a next step would be the coupling of this approach with a wider sampling exercise in order to evaluate the relative significance of different sources.

## Supplementary Information

Below is the link to the electronic supplementary material.

Supplementary file1 (PDF 14105 KB)

Supplementary file2 (PDF 12830 KB)

## Data Availability

Data will be made available on reasonable request.
