# Supplementary material for: What the presence of regulated chemical elements in beached lacustrine plastics can tell us: the case of Swiss lakes
Source: Environ Monit Assess. 2021 Oct 6;193(11):693. doi: 10.1007/s10661-021-09384-5 (PMC8492568; doi:10.1007/s10661-021-09384-5)
Supplement: Supplementary file 1 — Supplementary file1 (PDF 14105 KB) [file 10661_2021_9384_MOESM1_ESM.pdf]

Supplementary Information -1

What the presence of regulated chemical elements in beached lacustrine plastics can tell us: the case of Swiss lakes

Montserrat Filella[1], Juan-Carlos Rodríguez-Murillo[2] and Andrew Turner[3]

[1]Department F.-A. Forel, University of Geneva, Boulevard Carl-Vogt 66, CH-1205 Geneva, Switzerland

[2]Museo Nacional de Ciencias Naturales, CSIC, Serrano 115 dpdo., E-28006 Madrid, Spain

[3]School of Geography, Earth and Environmental Sciences, Plymouth University, Drake Circus, Plymouth PL4 8AA, UK

Lake Bienne – Ipsach (beach #1)

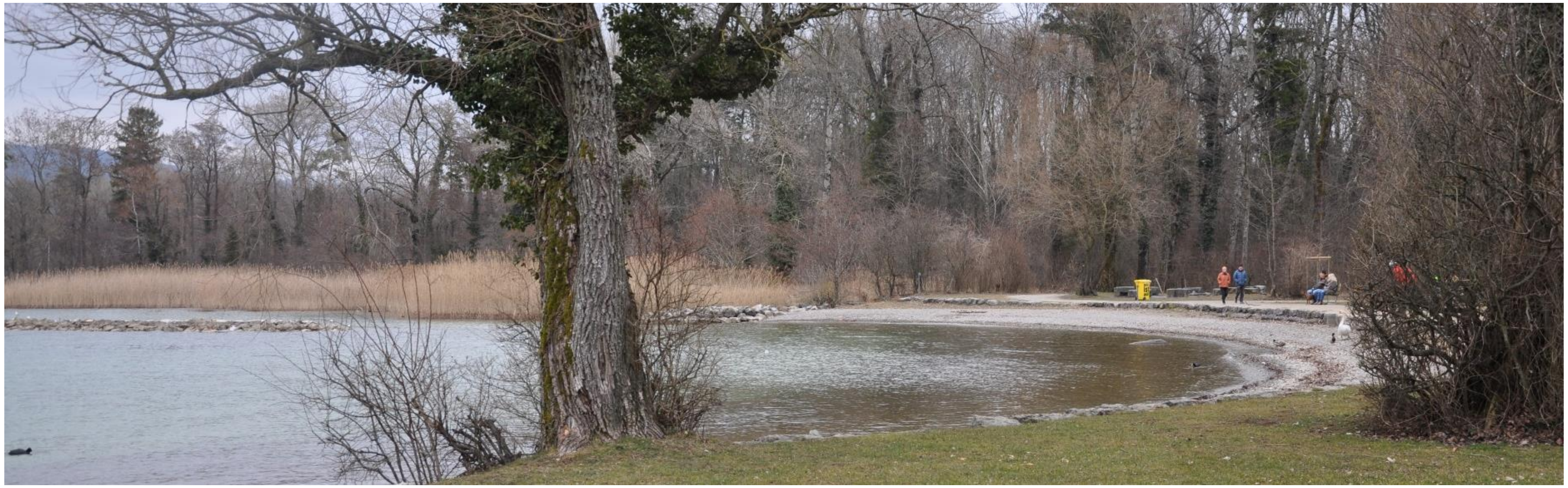

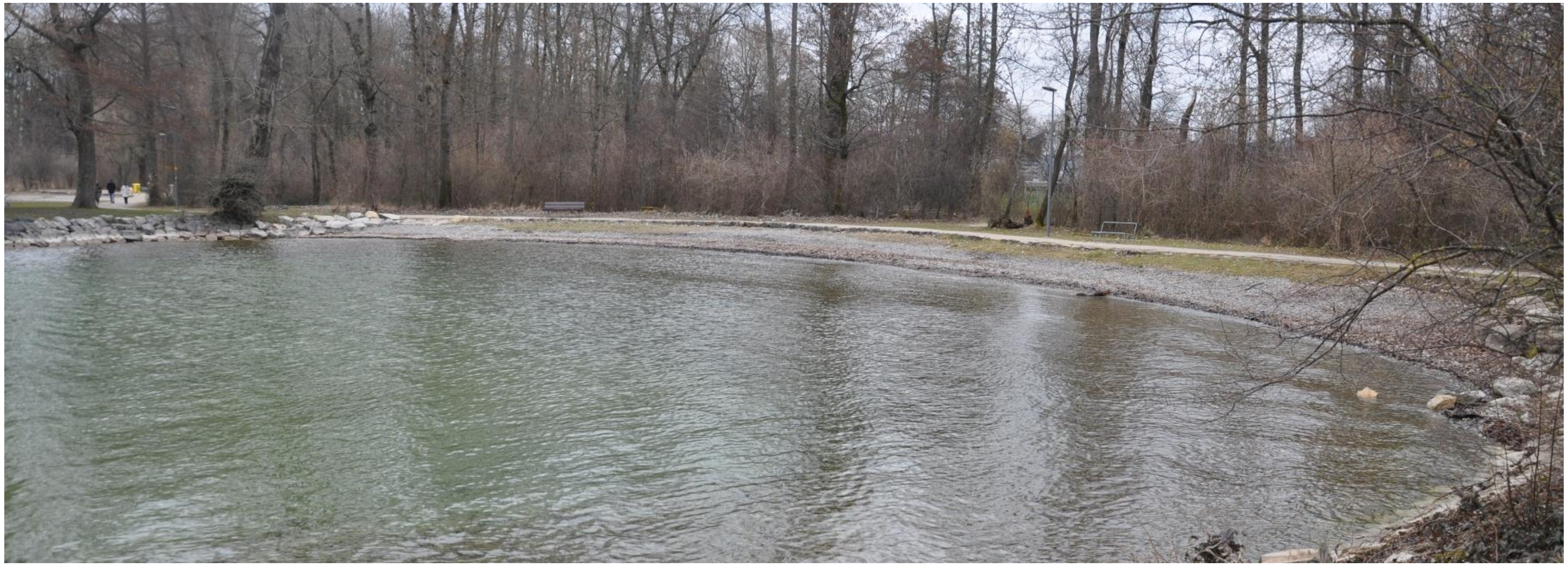

Lake Bienne – Ipsach (beach #1)

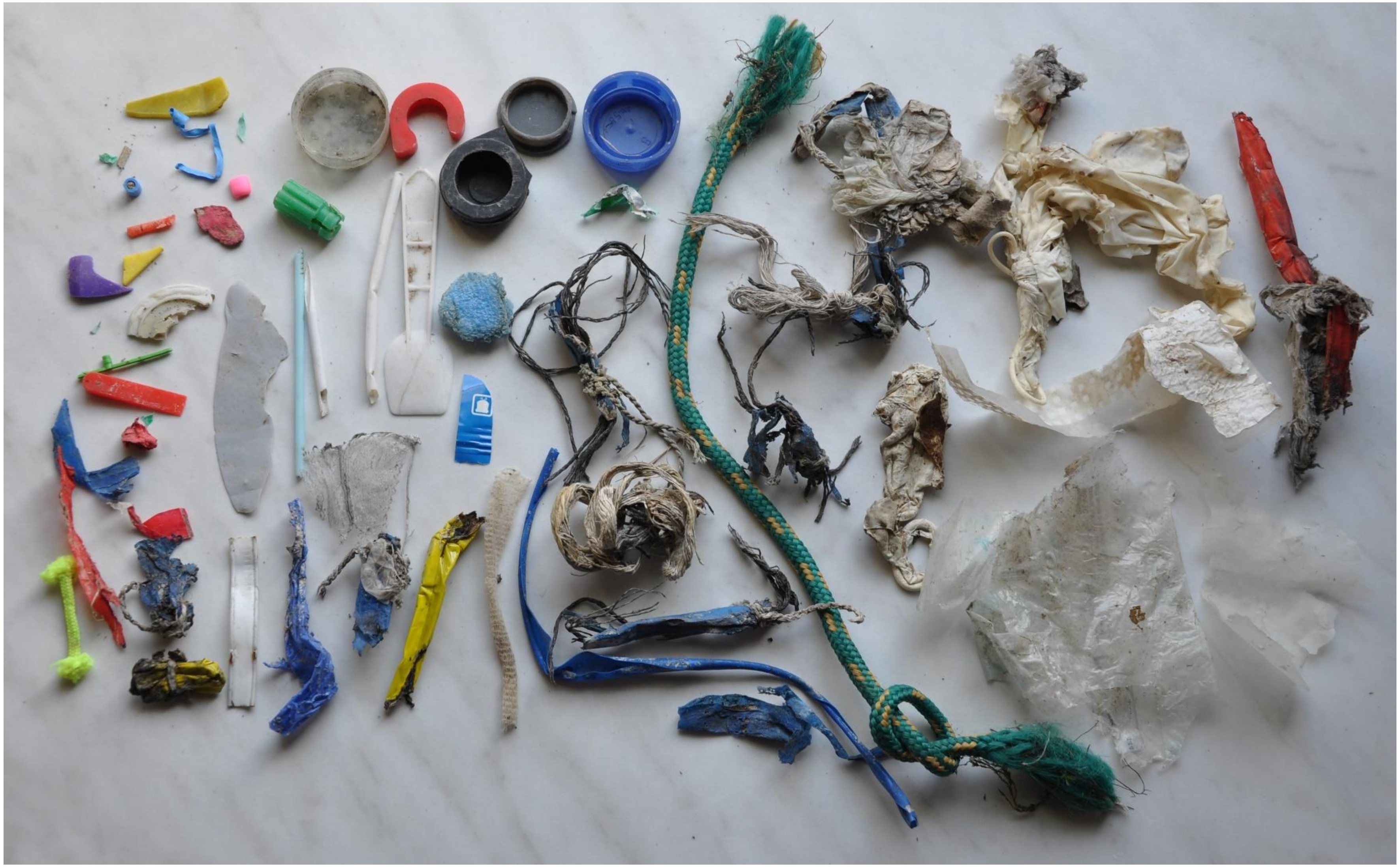

Lake Bienne – La Neuveville (beach # 2)

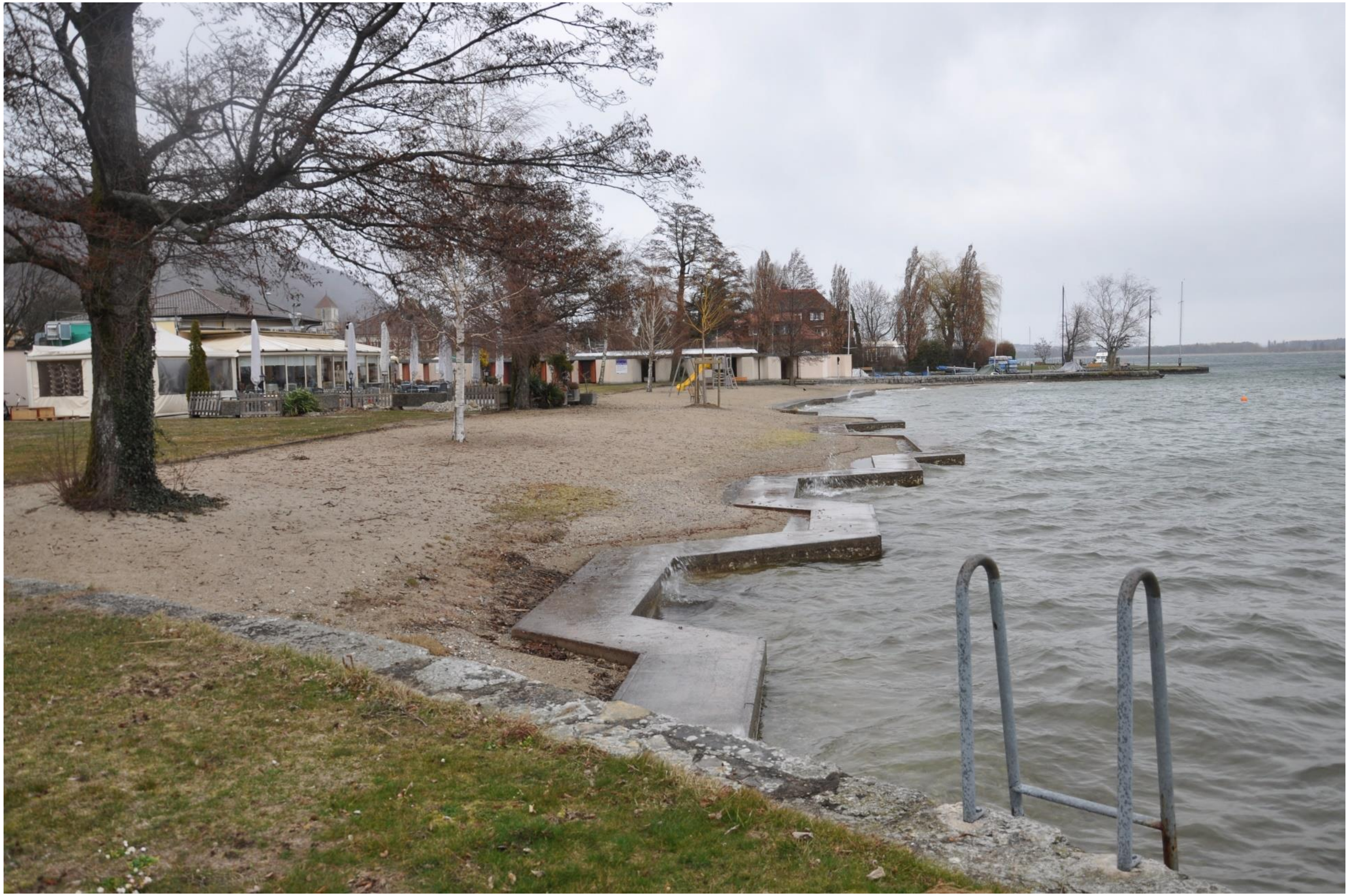

Lake Bienne – La Neuveville (beach #2)

Picture of samples missing

Lake Bienne – Lüscherz (beach #3)

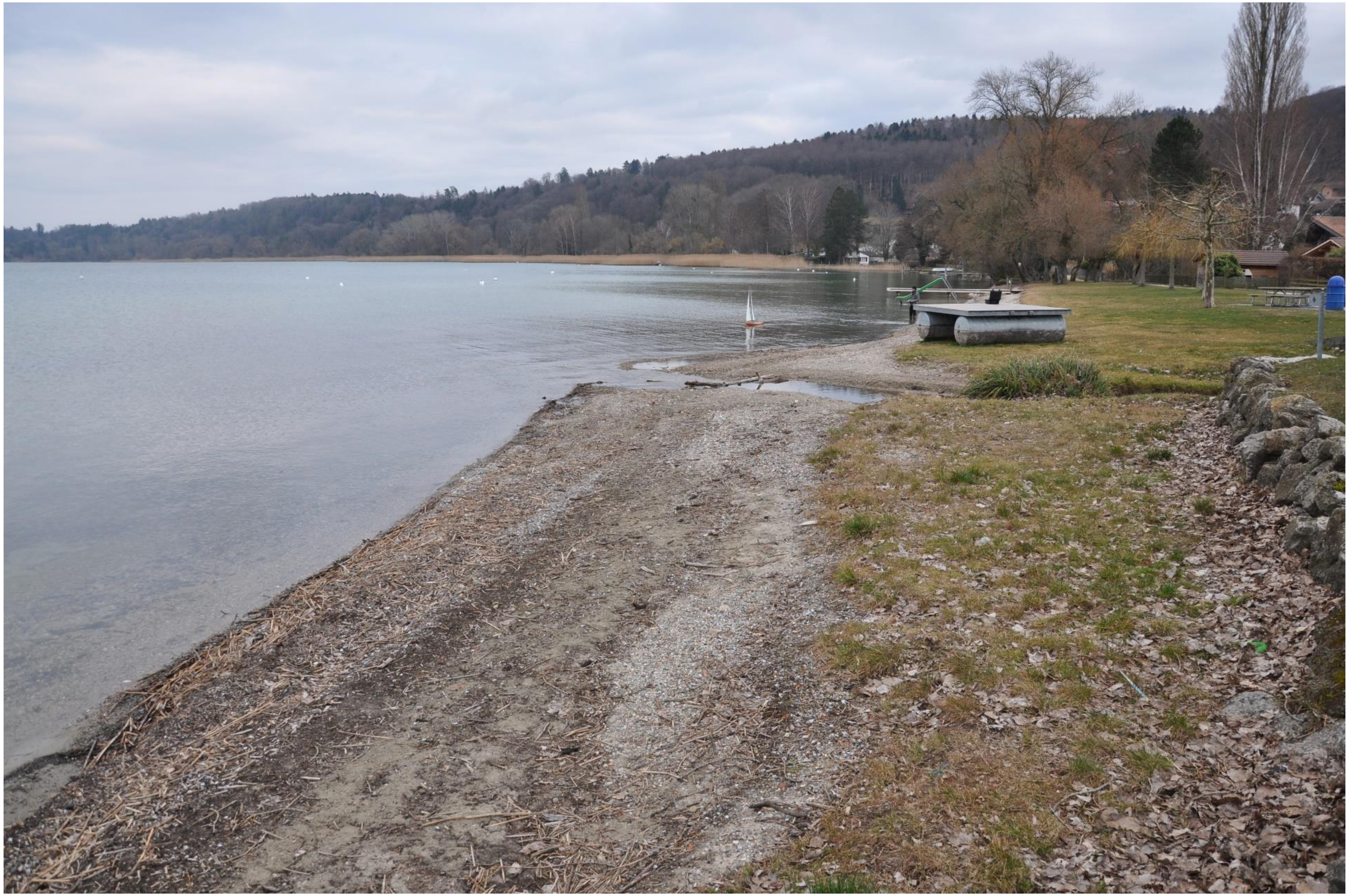

Lake Bienne – Lüscherz (beach #3)

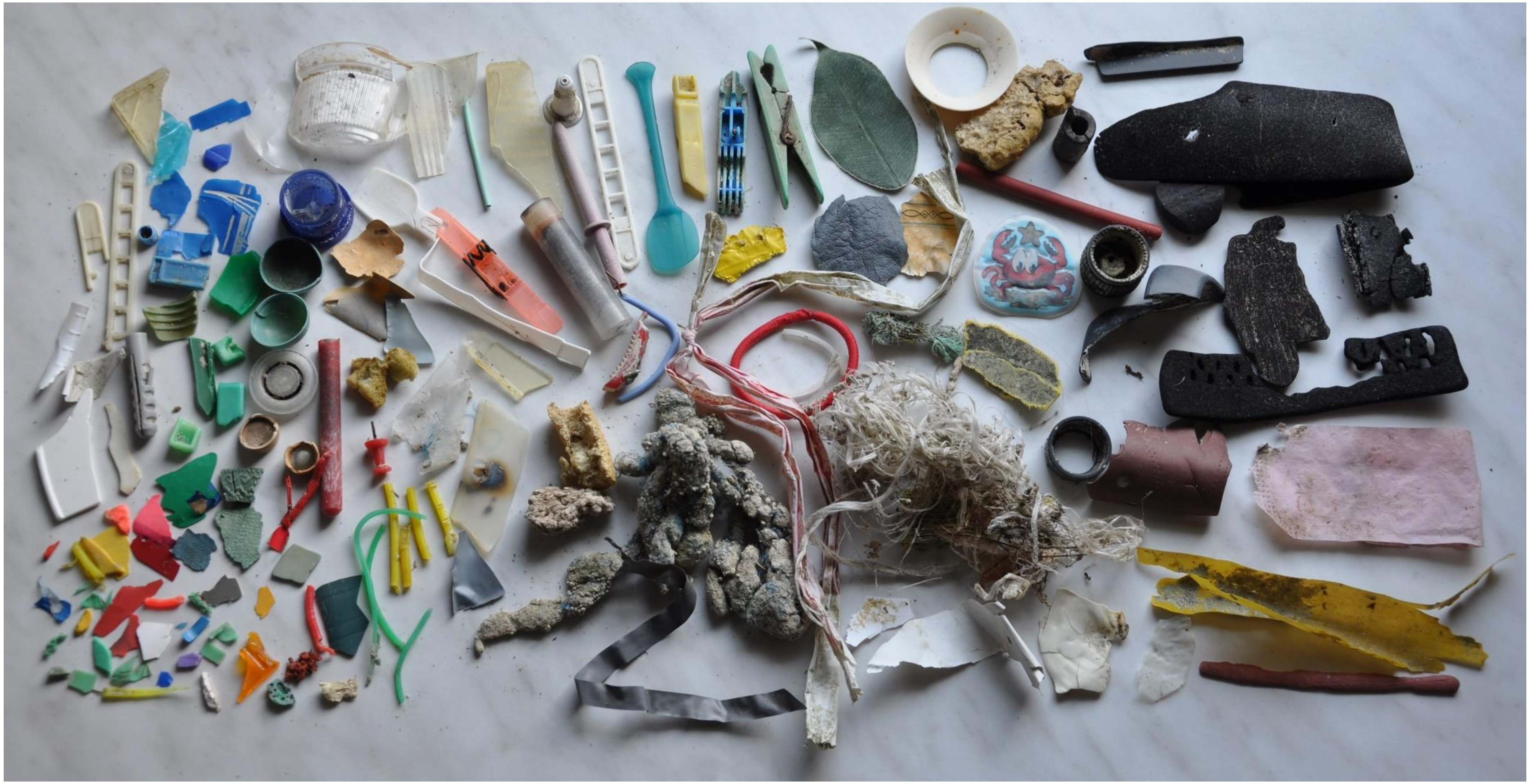

Lake Brienz – General overview

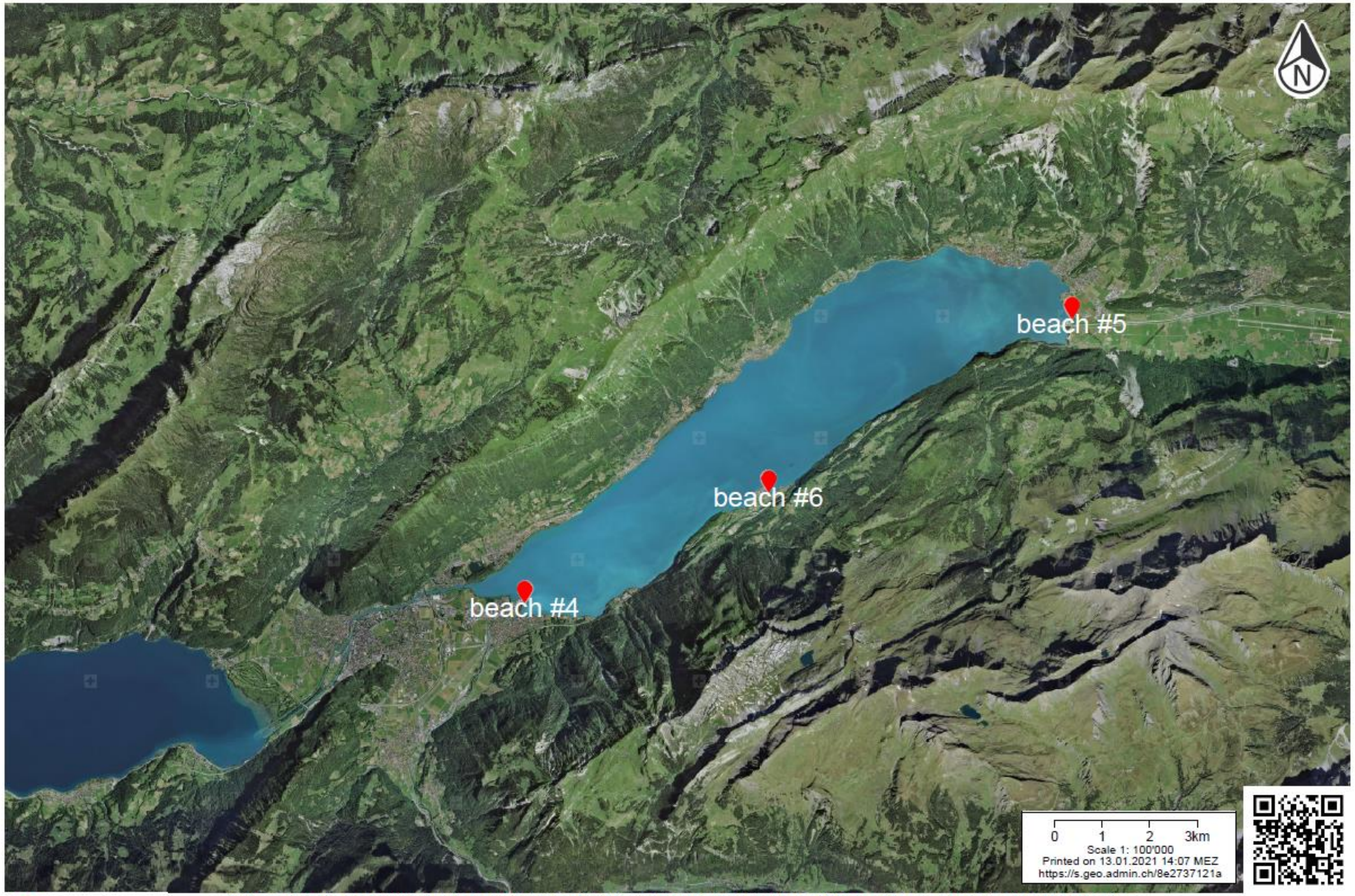

Schweizerische Eidgenossenschaft
Confédération suisse
Confederazione Svizzera
Confederaziun svizra
In collaboration with the cantons

www.geo.admin.ch is a portal provided by the Federal Authorities of the Swiss Confederation to gain insight on publicly accessible geographical information, data and services
Limitation of liability. Although every care has been taken by the Federal Authorities to ensure the accuracy of the information published, no warranty can be given in respect of the accuracy, reliability, up-to-dateness or completeness of this information. Copyright, Swiss federal authorities. http://www.disclaimer.admin.ch/terms_and_conditions.html
© CNES, Spot Image, swisstopo, NPOC, public.geo.admin.ch

Lake Brienz – Details of beaches # 4 and 5 (see discussion in text)

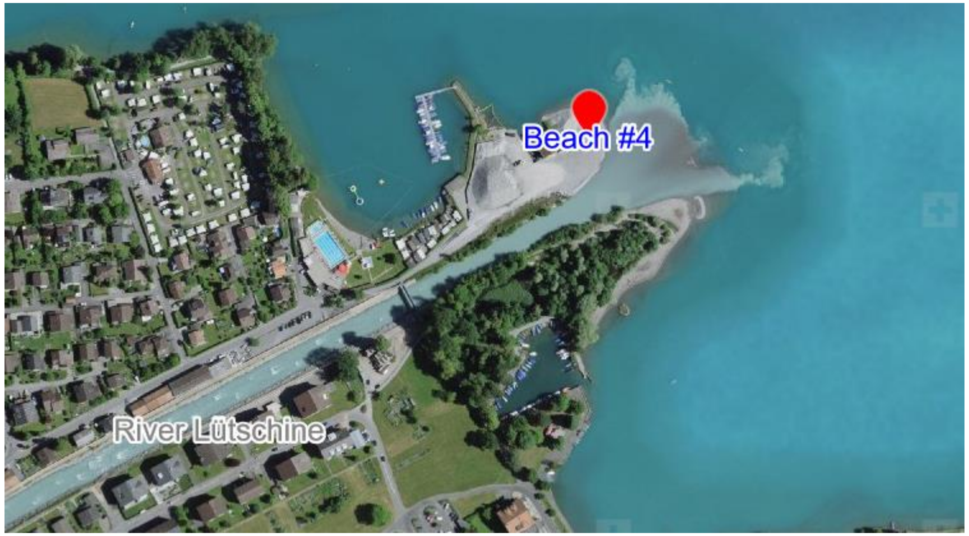

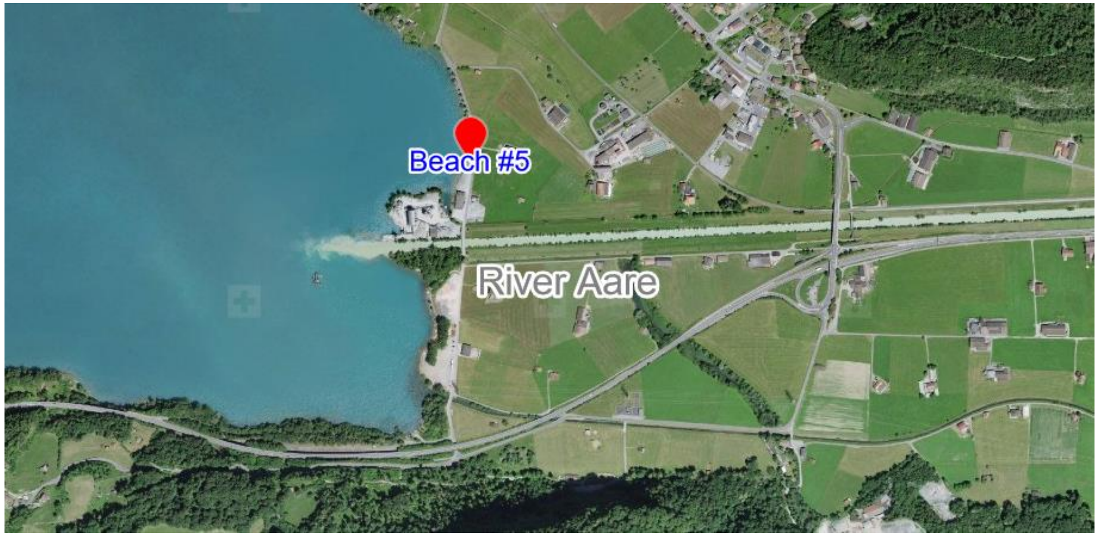

Lake Brienz – Lütschisand (beach #4)

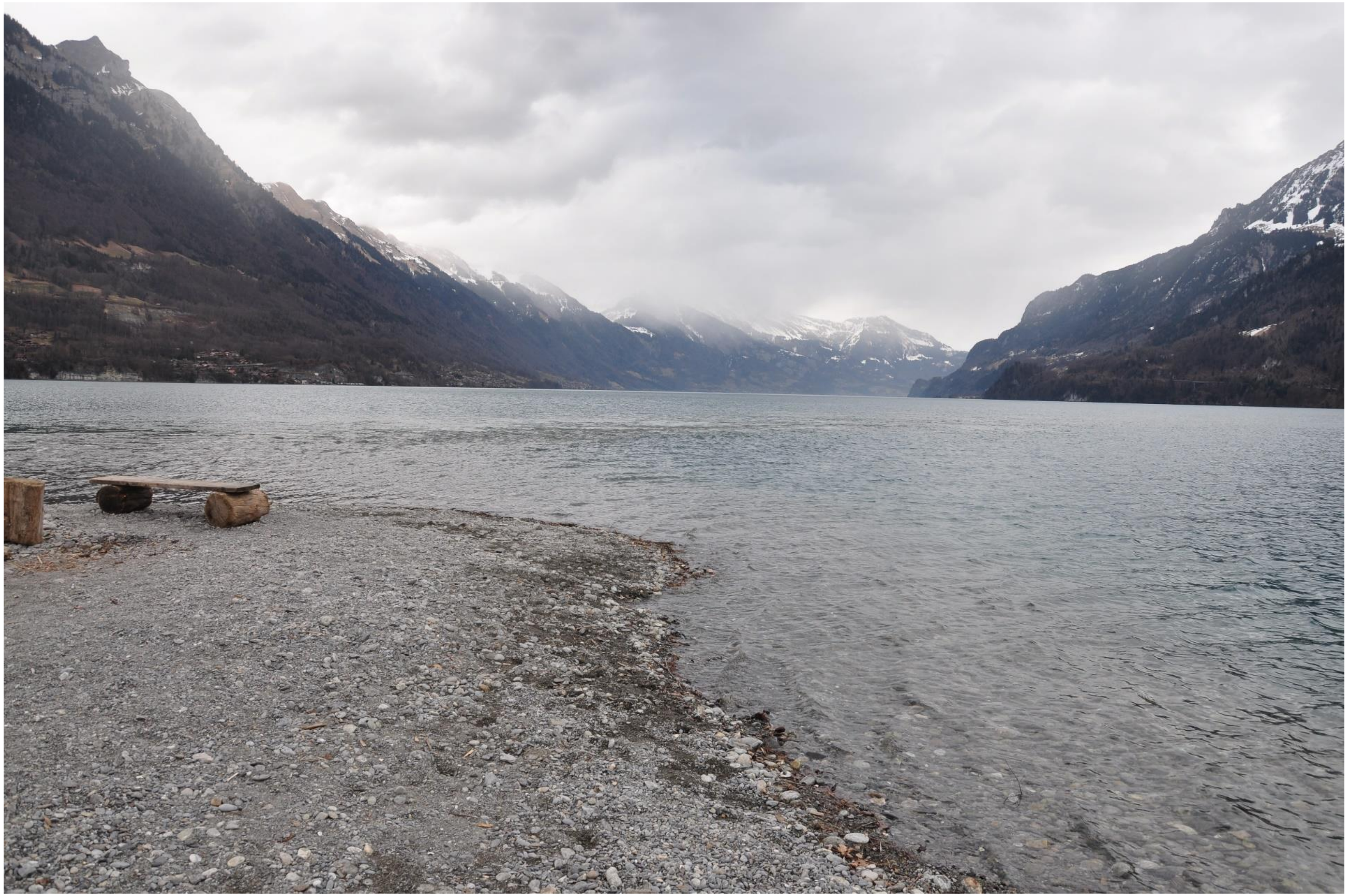

Lake Brienz – Lütschisand (beach #4)

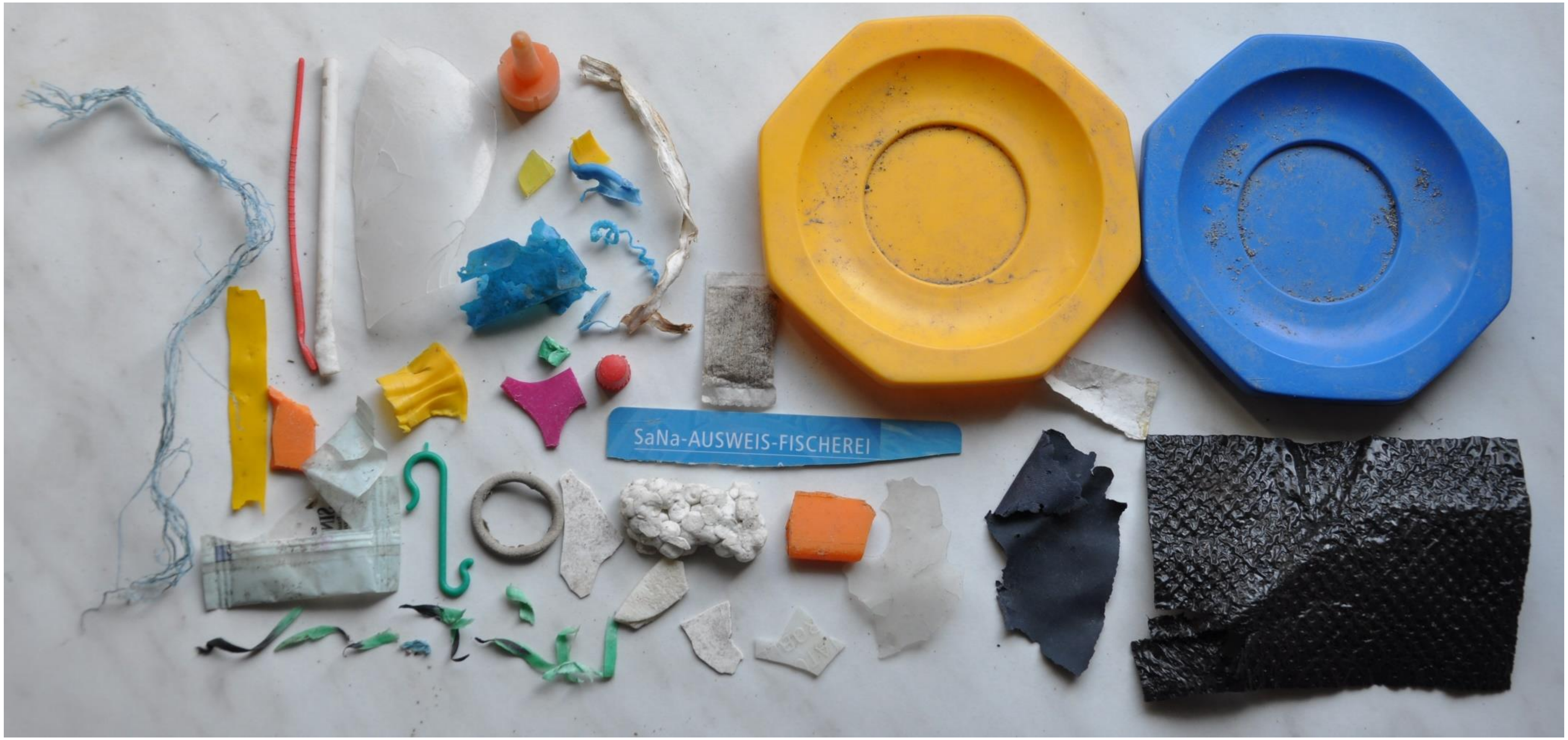

Lake Brienz – Brienz (beach #5)

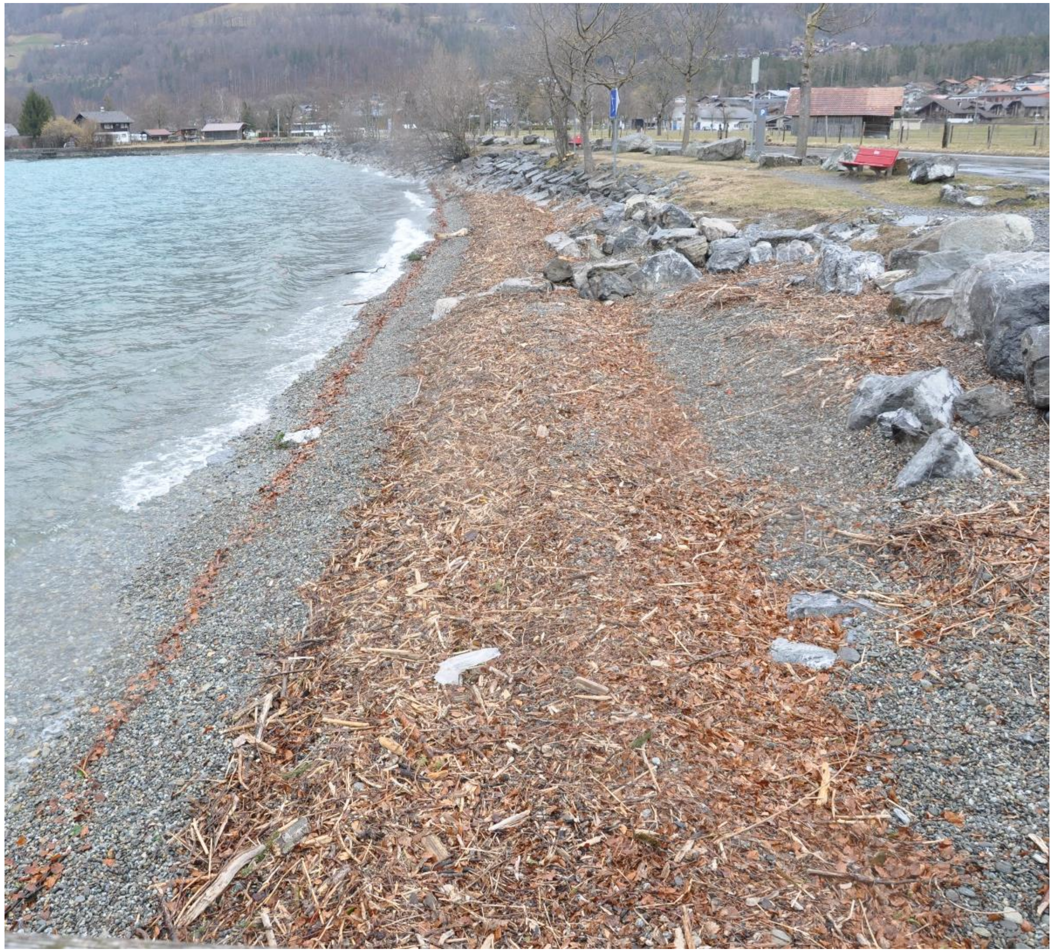

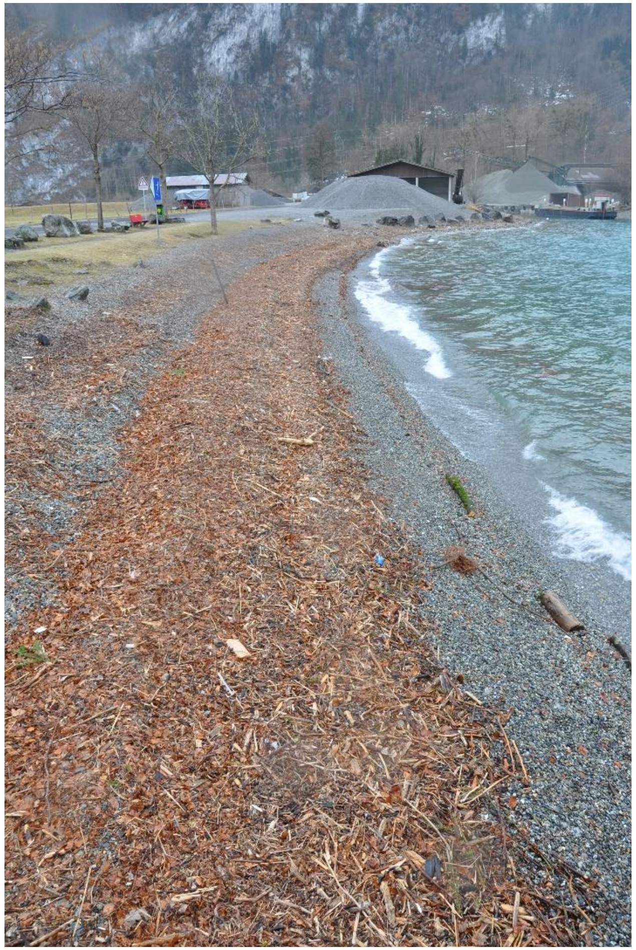

Lake Brienz – Brien: red objects (beach #5)

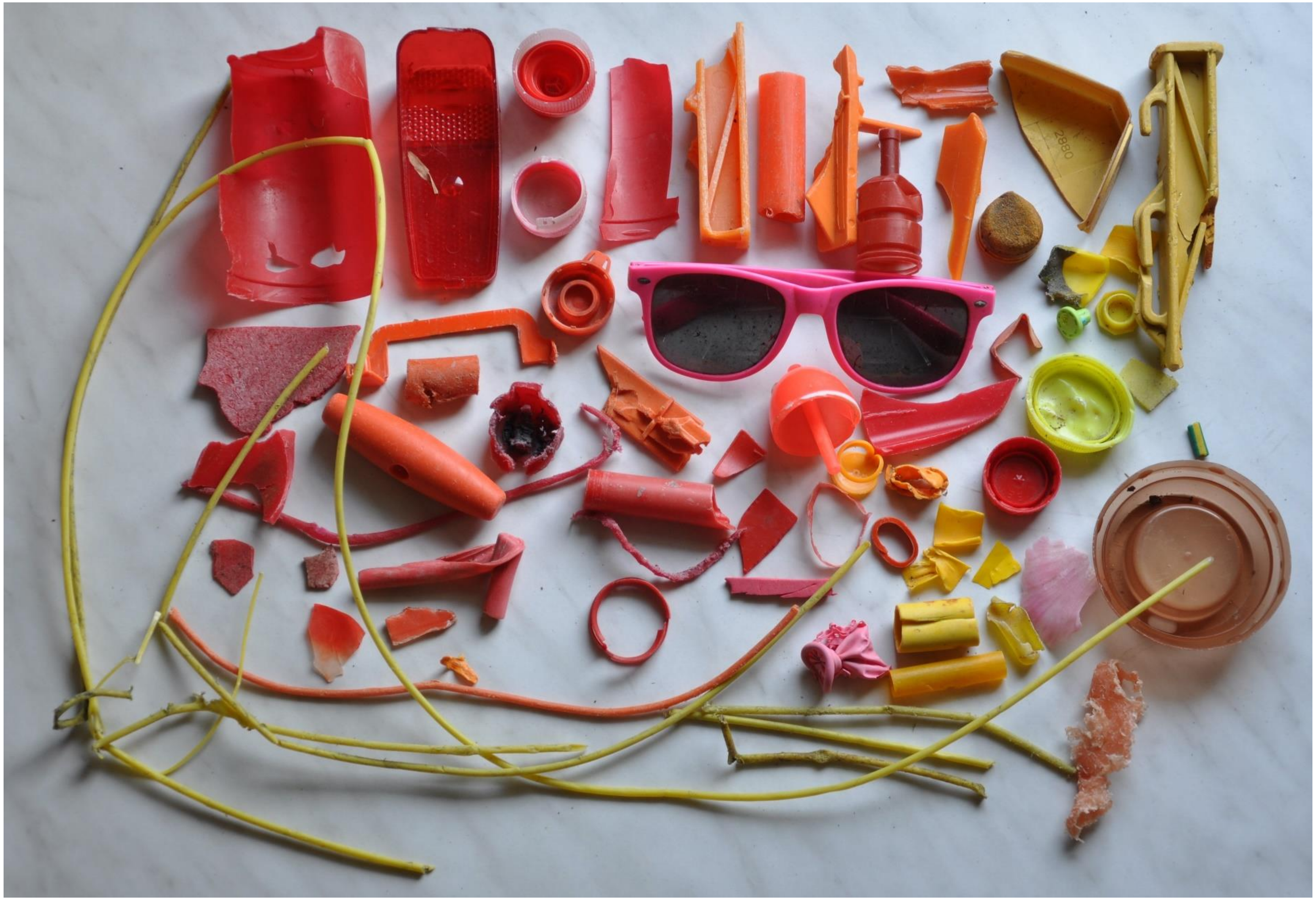

Lake Brienz – Brien: mostly blue objects (beach #5)

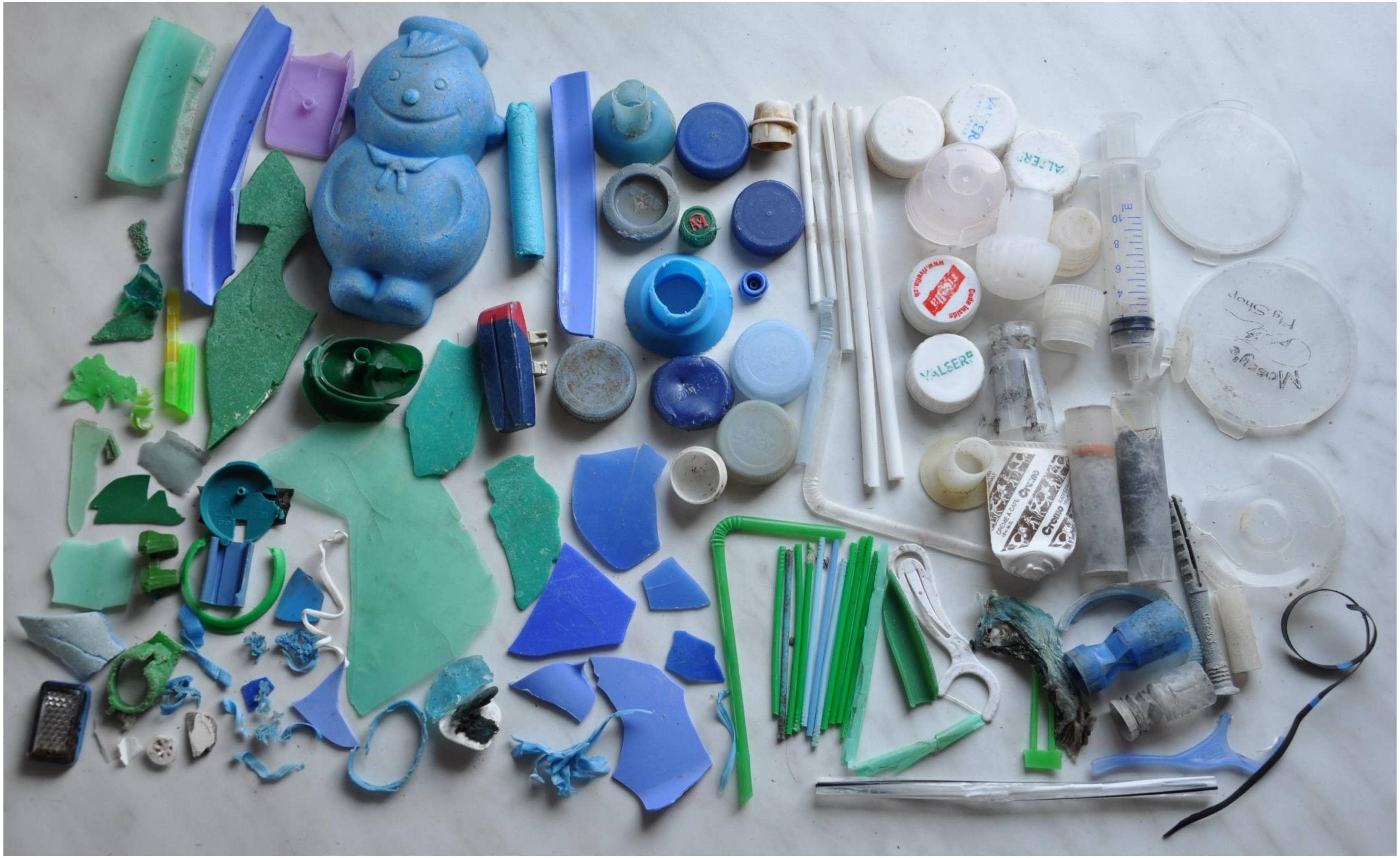

Lake Brienz – Brien: dark objects (beach #5)

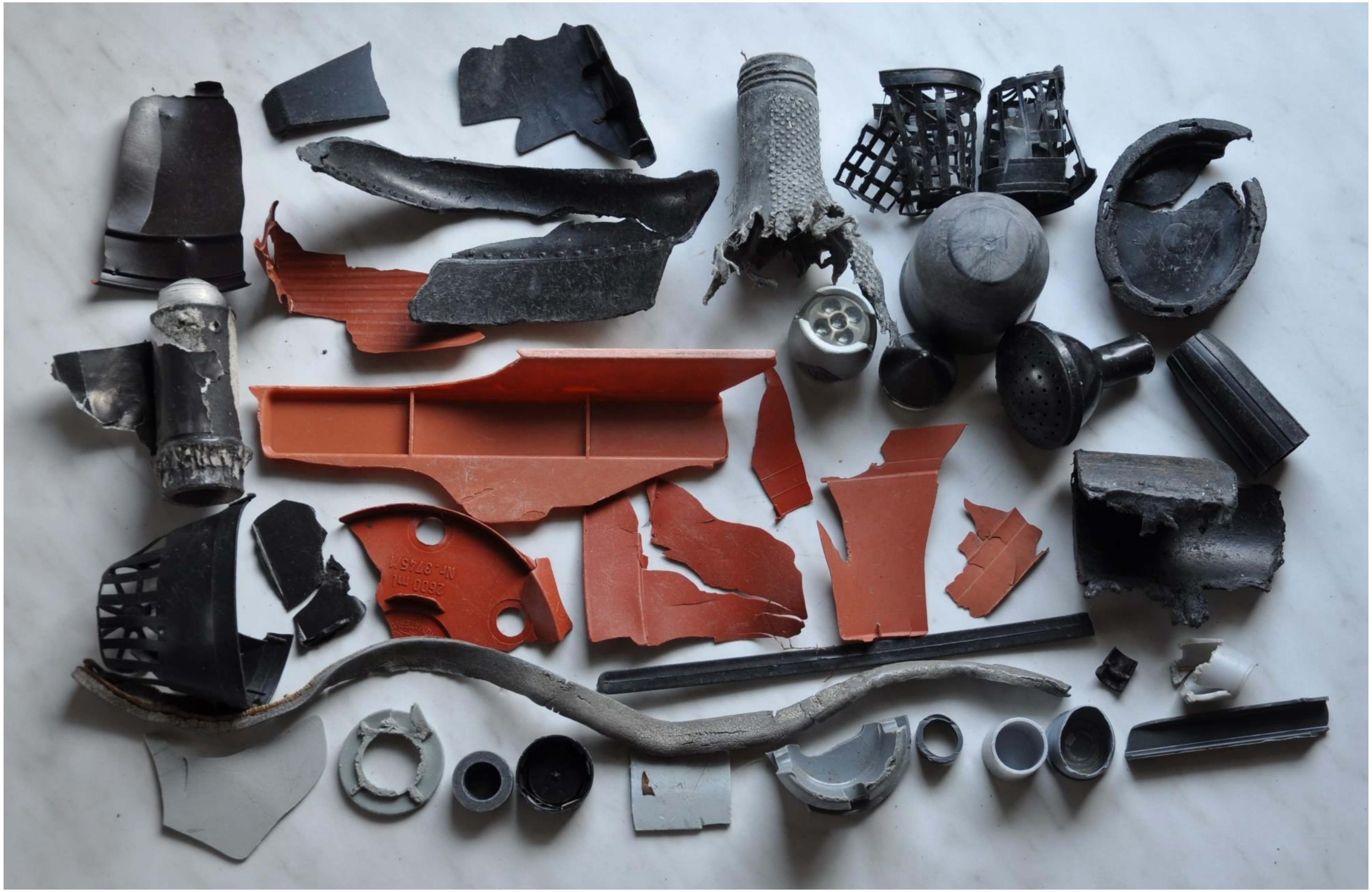

Lake Brienz – Brien: white objects (beach #5)

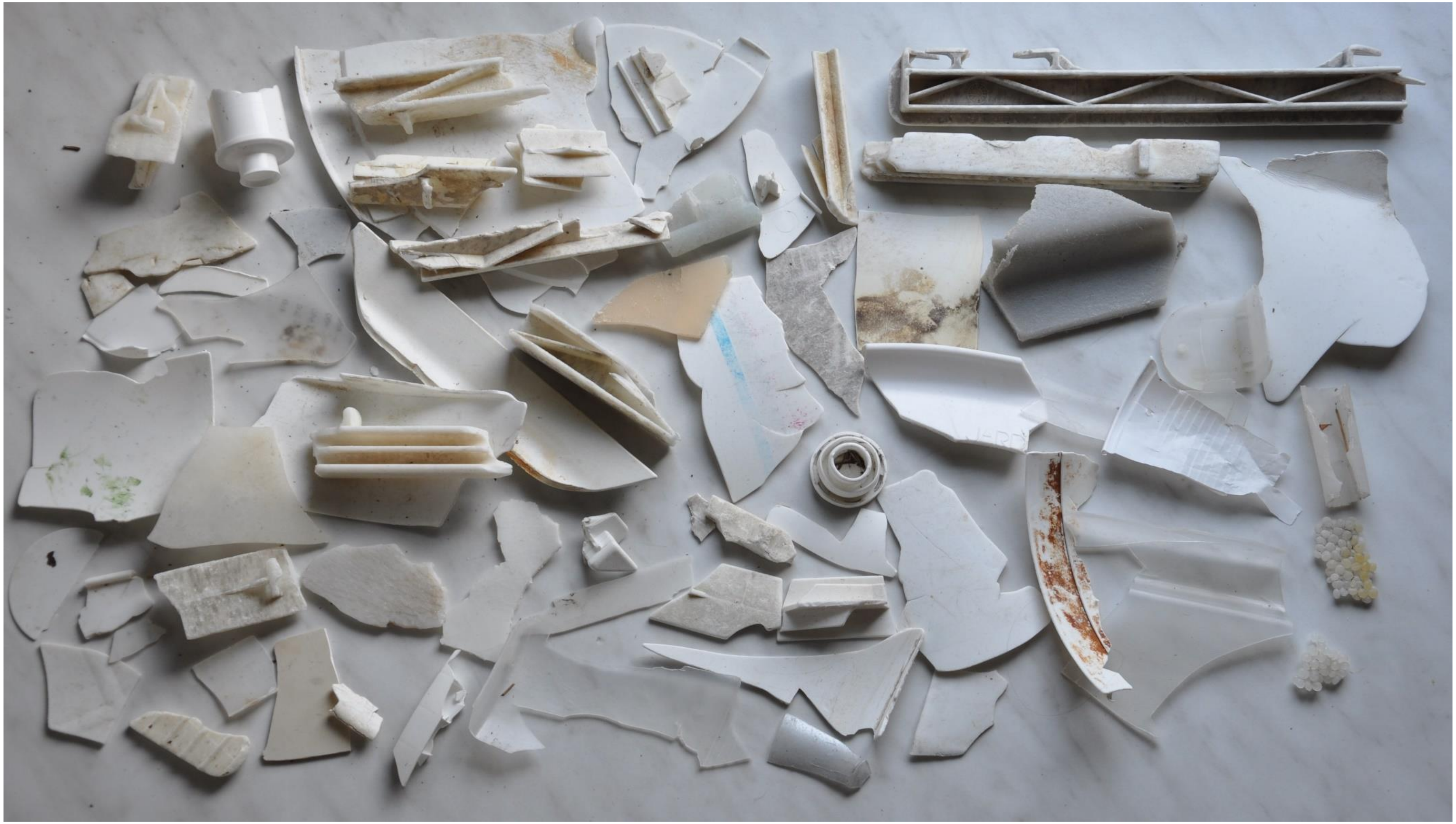

Brienz – Iseltwald (beach #6)

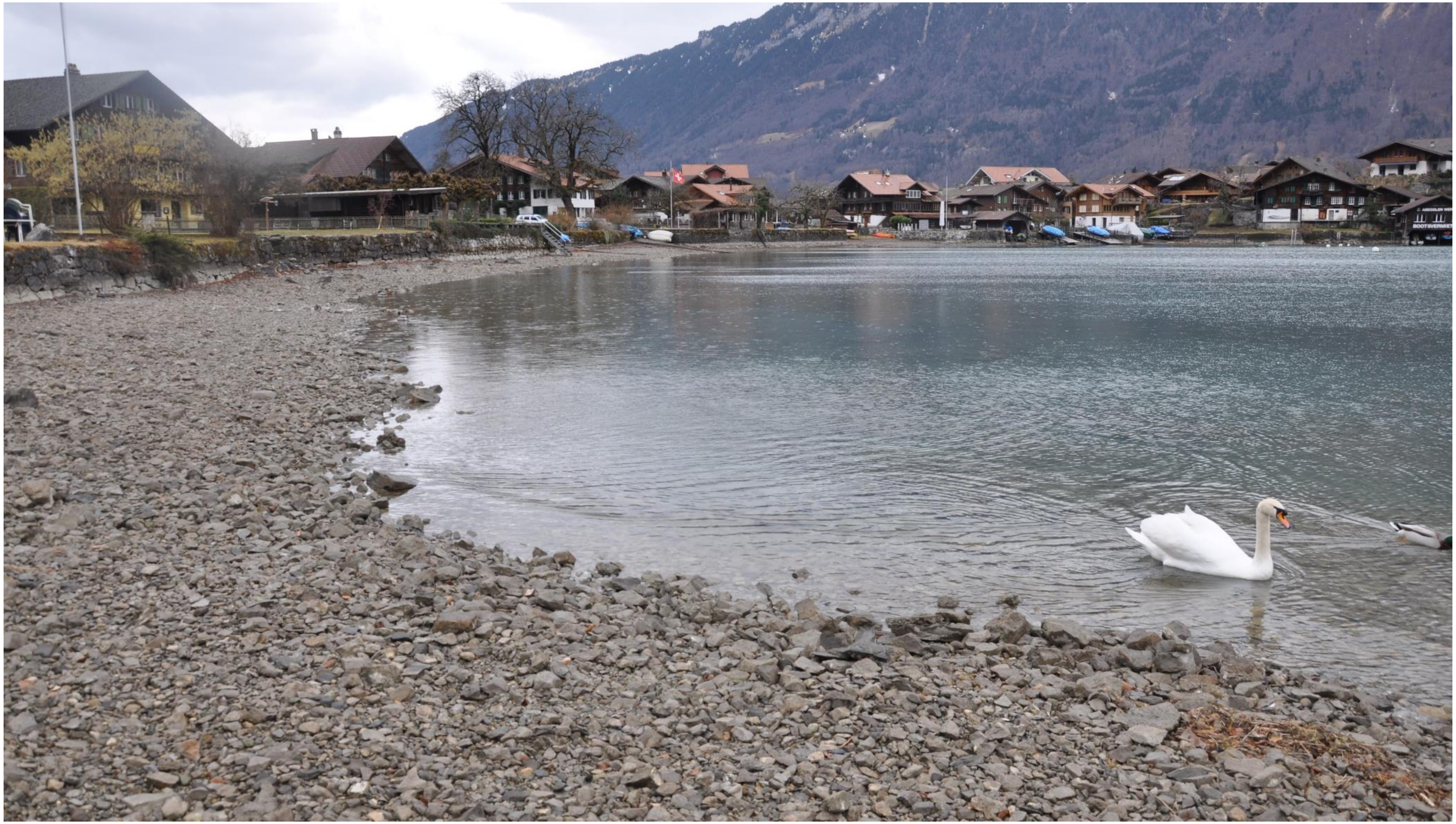

Brienz – Iseltwald (beach #6)

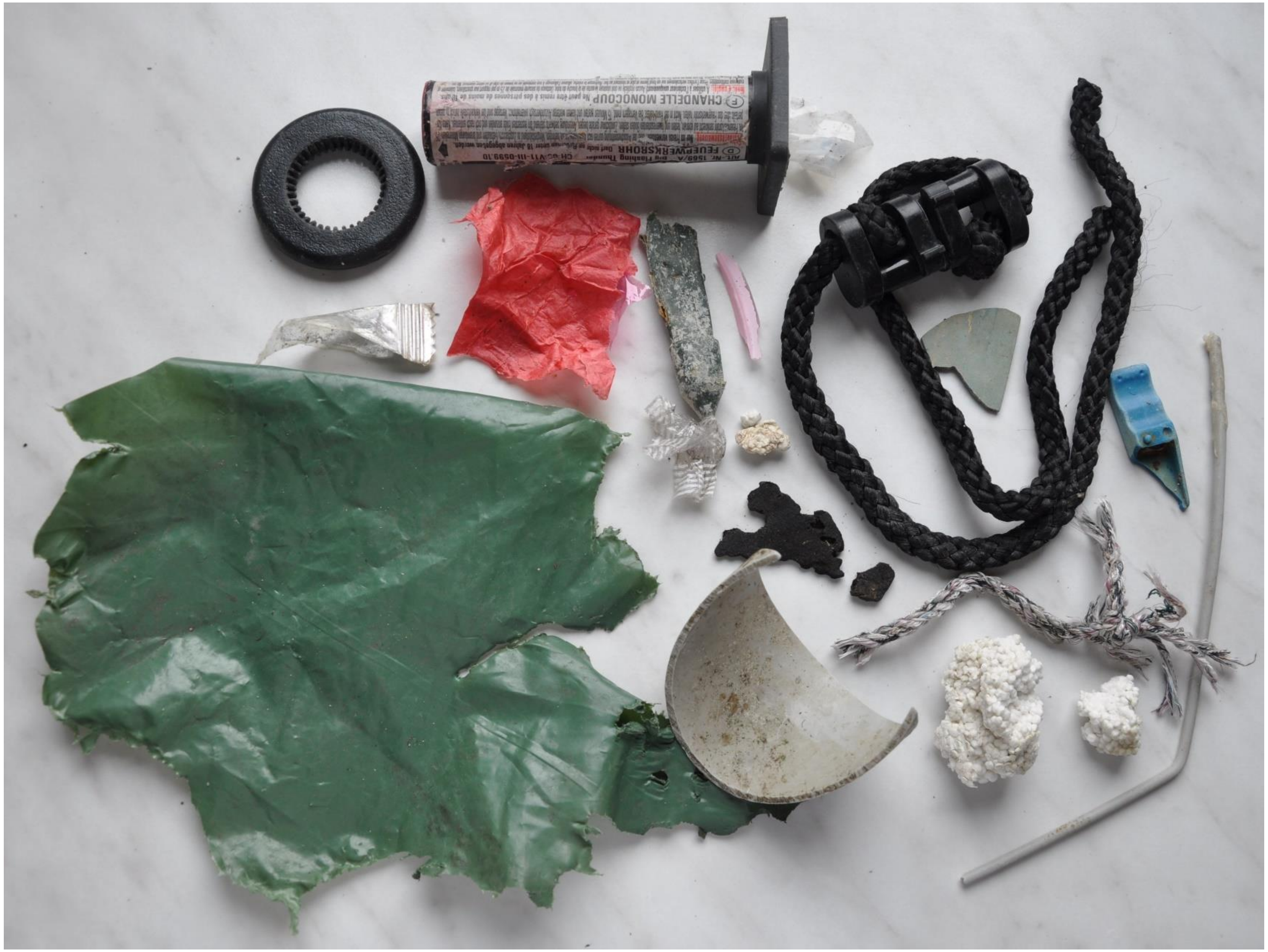

Lake Constance – General overview

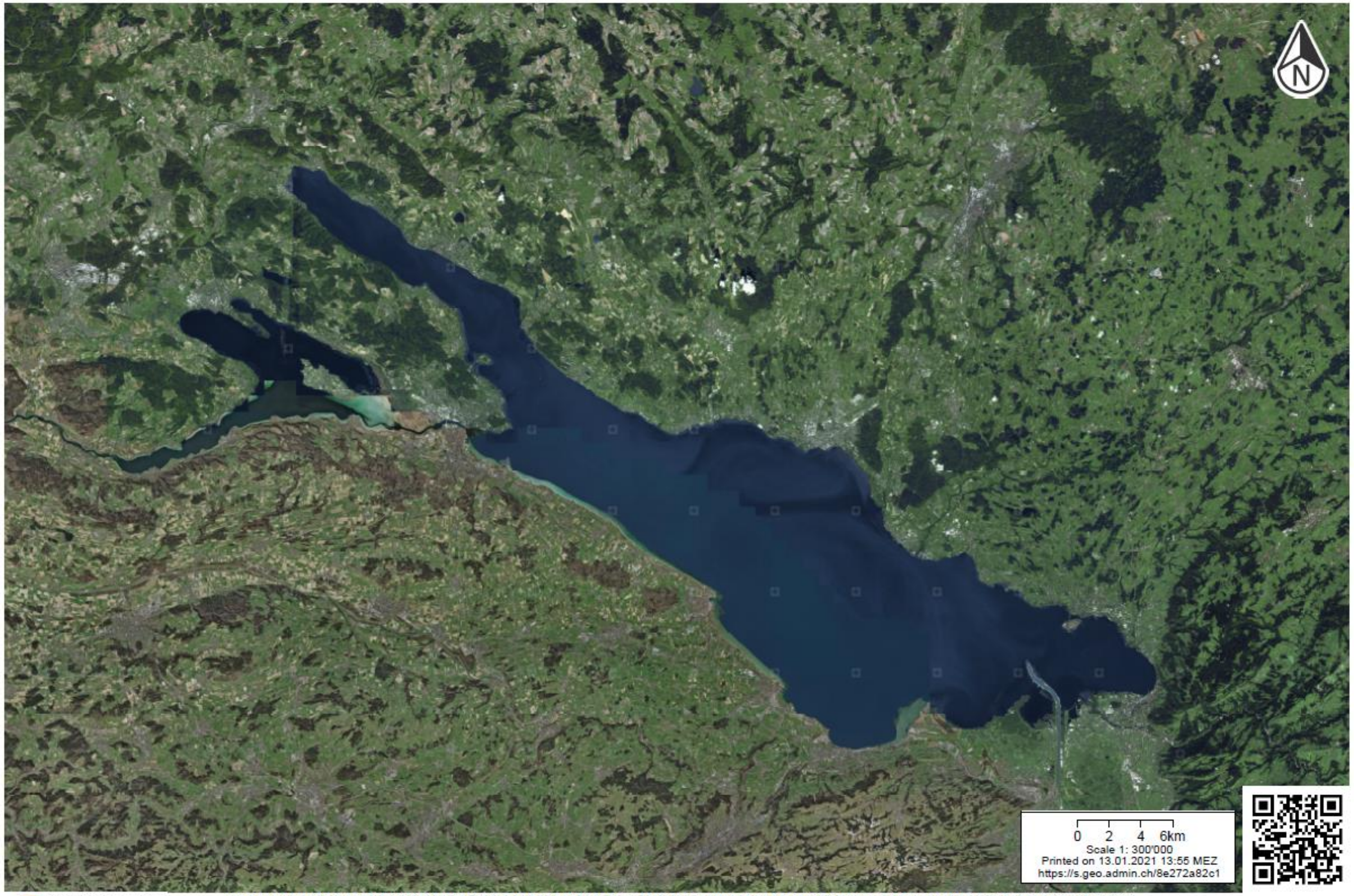

Schweizerische Eidgenossenschaft
Confédération suisse
Confederazione Svizzera
Confederaziun svizra
In collaboration with the cantons

www.geo.admin.ch is a portal provided by the Federal Authorities of the Swiss Confederation to gain insight on publicly accessible geographical information, data and services
Limitation of liability. Although every care has been taken by the Federal Authorities to ensure the accuracy of the information published, no warranty can be given in respect of the accuracy, reliability, up-to-dateness or completeness of this information. Copyright, Swiss federal authorities. http://www.disclaimer.admin.ch/terms_and_conditions.html
© CNES, Spot Image, swisstopo, NPOC, public.geo.admin.ch

Lake Constance – Details of beaches #9, 14, 15 and 19 (see discussion in text)

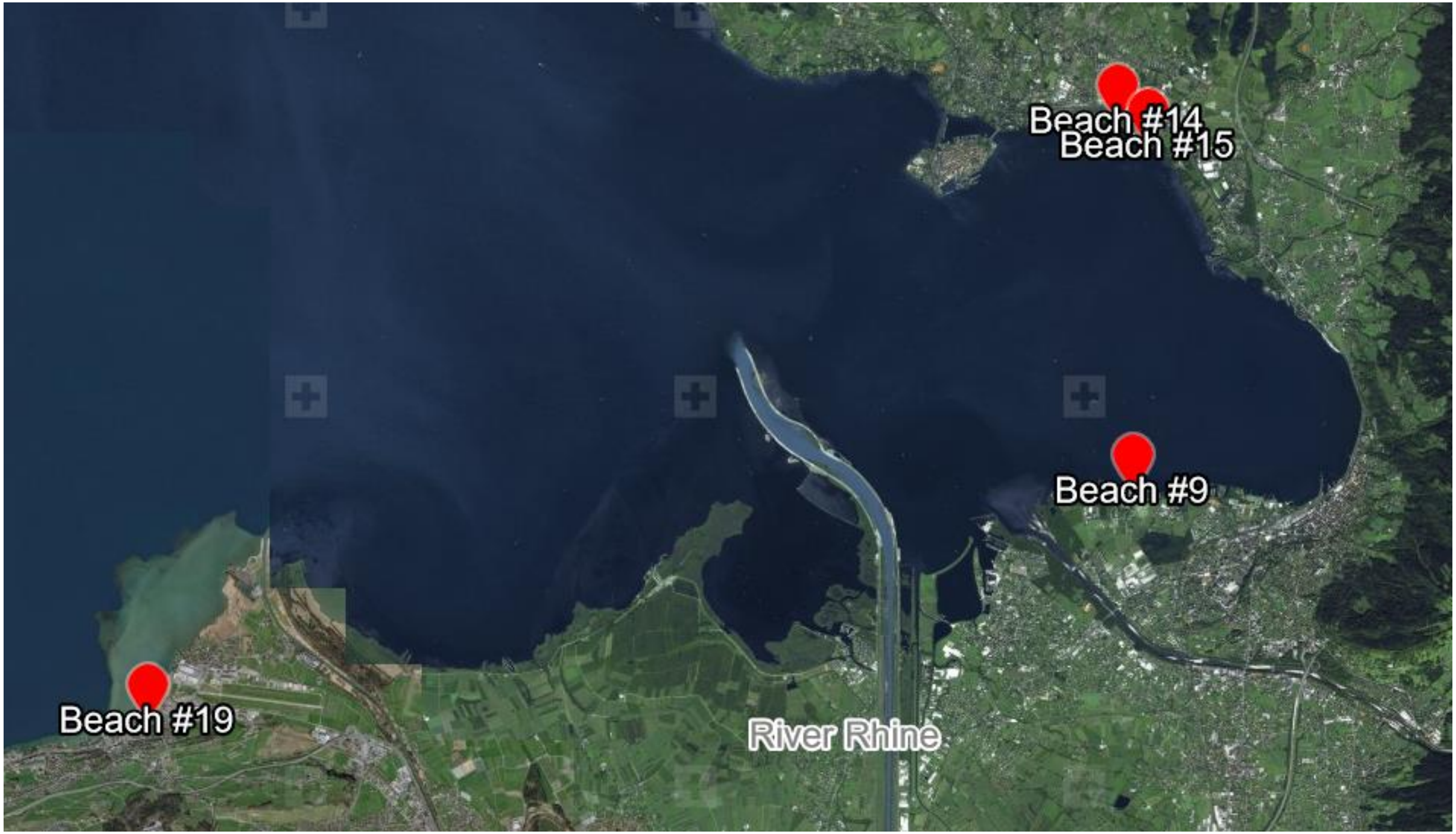

Lake Constance – Arbon, Buchhorn Strandbad (beach #7)

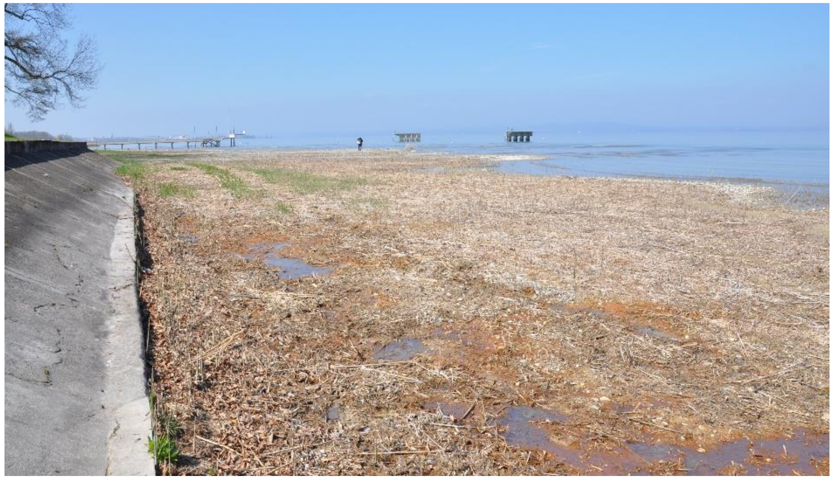

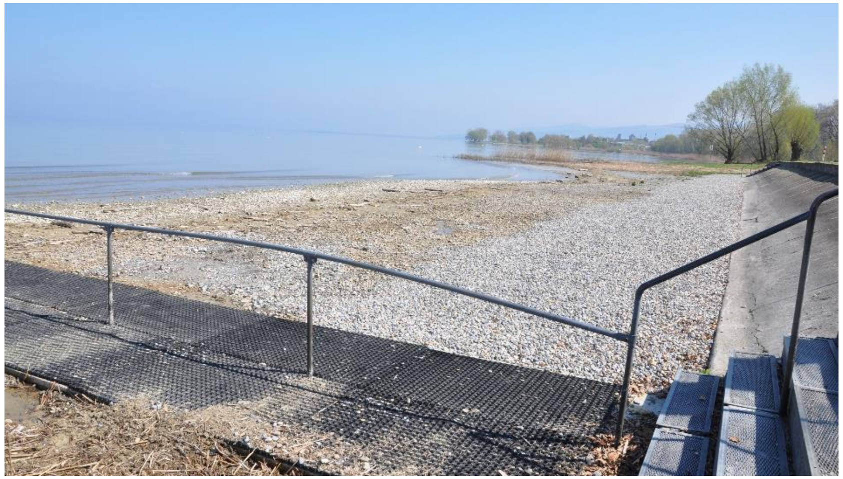

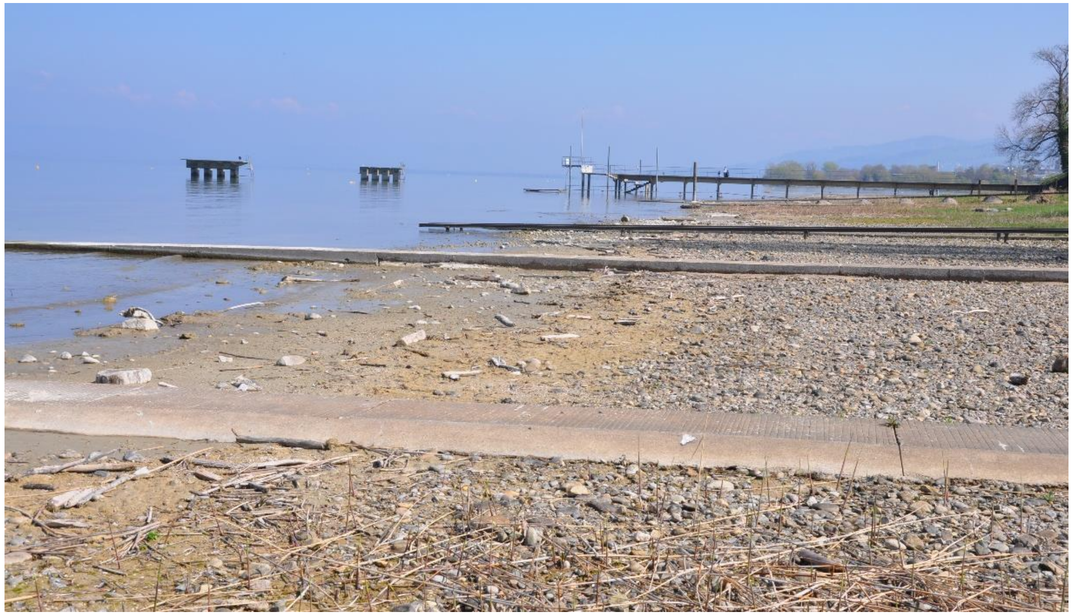

Lake Constance – Arbon, Buchhorn Strandbad (beach #7)

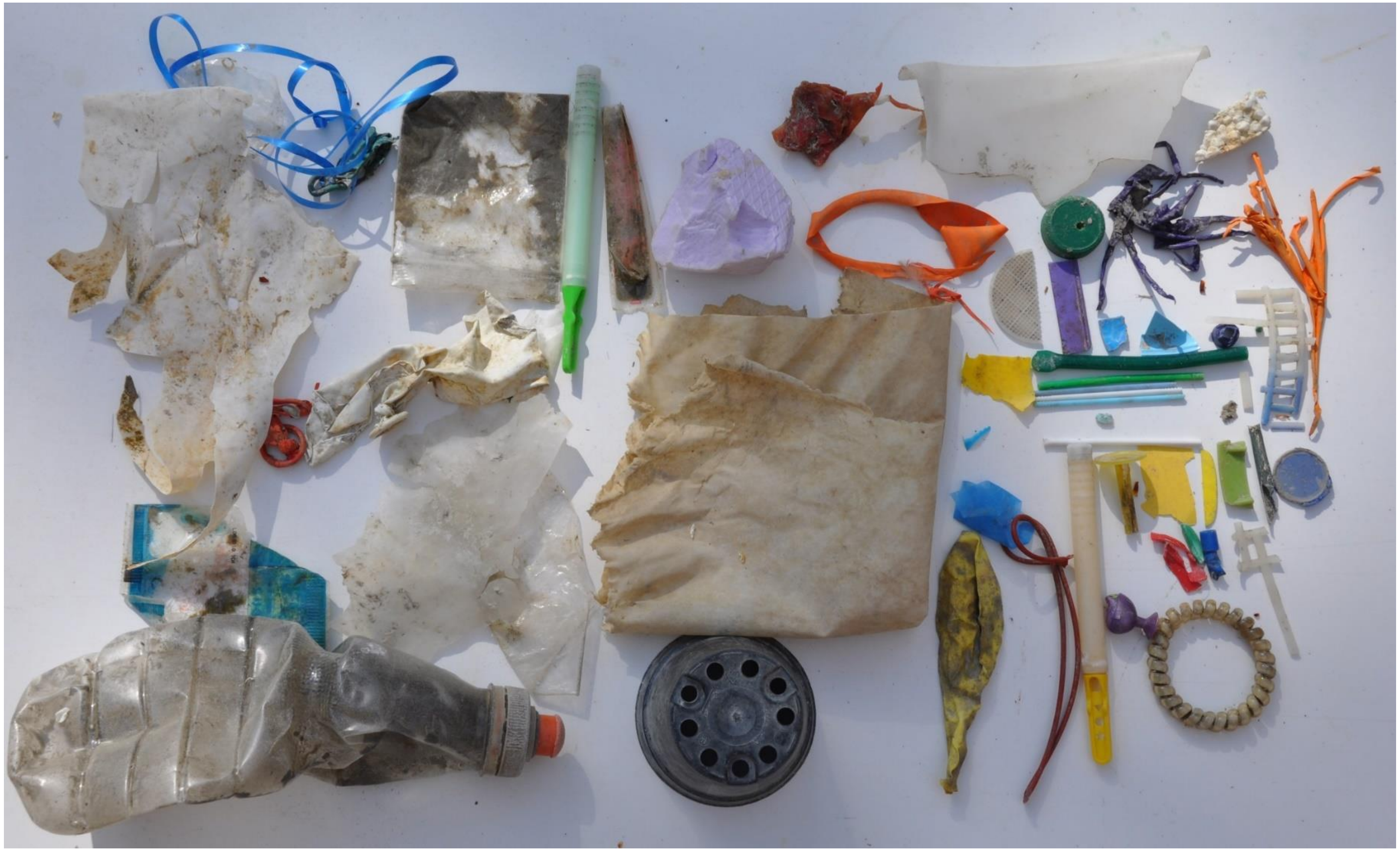

Lake Constance – Bodman (Germany) (beach #8)

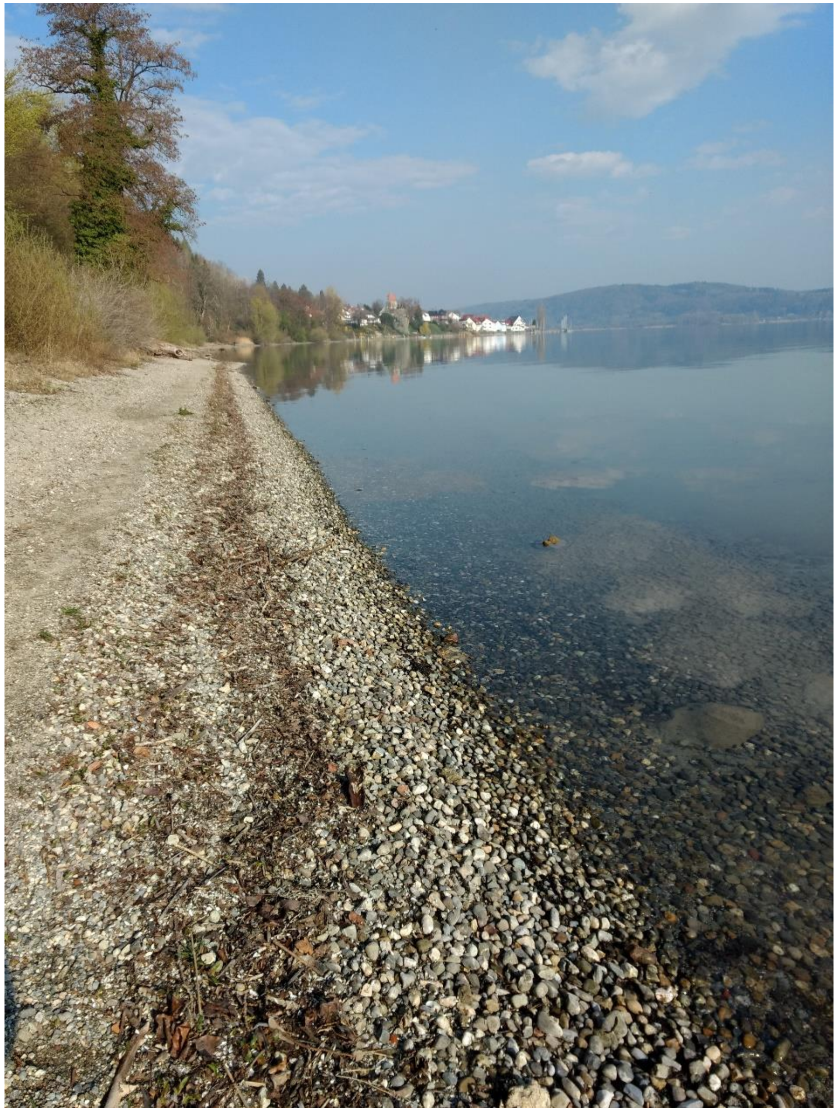

Lake Constance – Bodman (Germany) (beach #8)

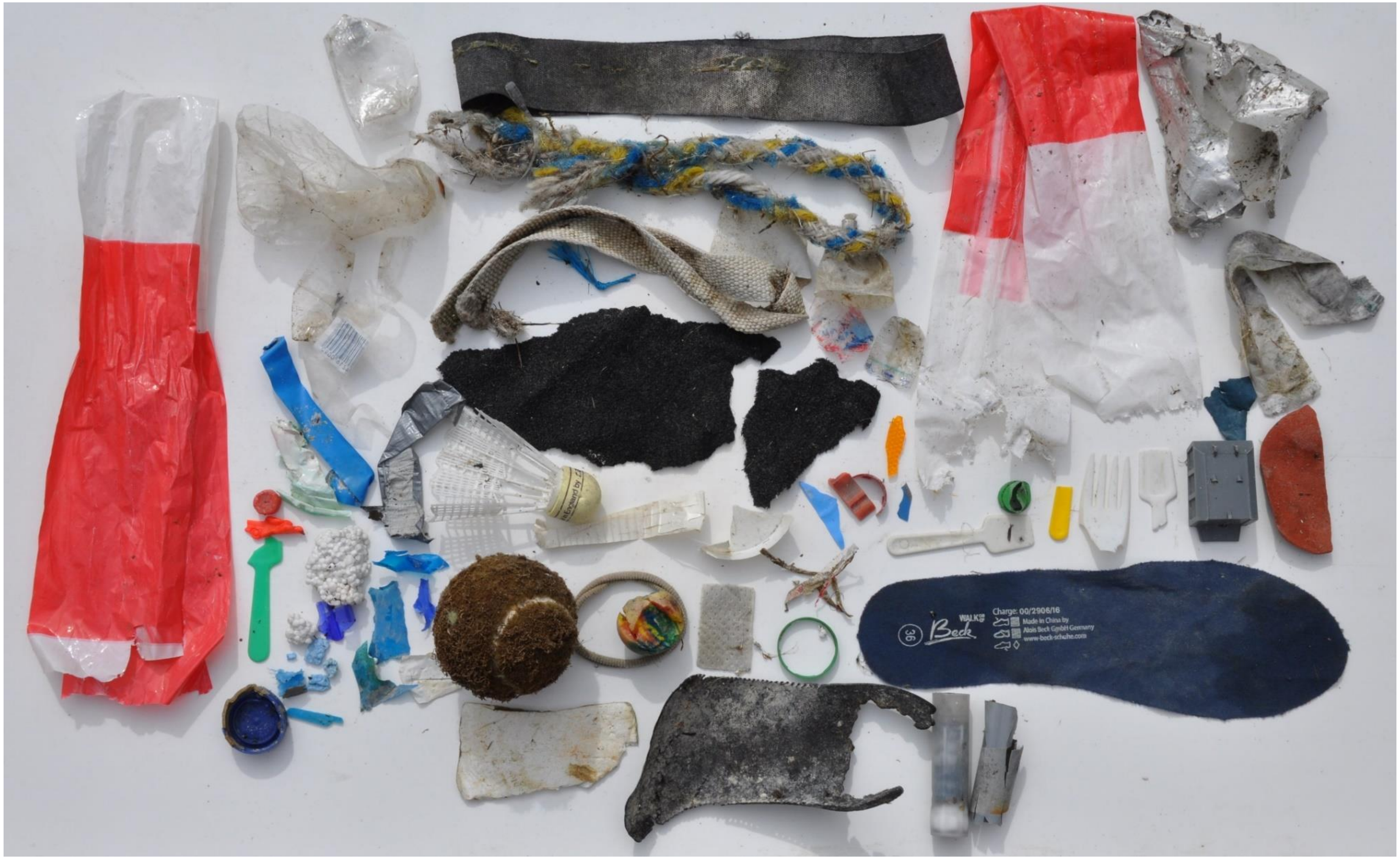

Lake Constance –Bregenz Seecamping (Austria) (beach #9)

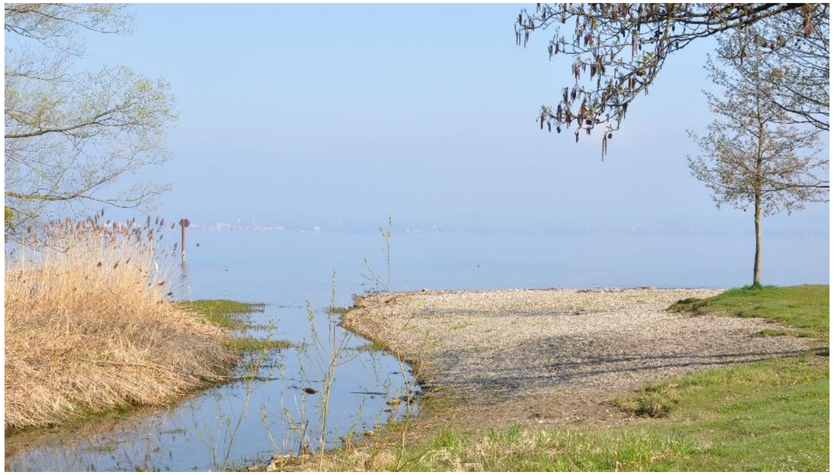

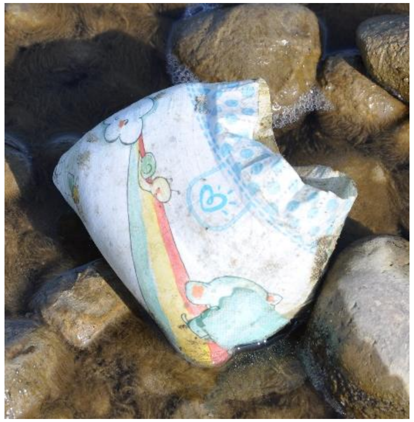

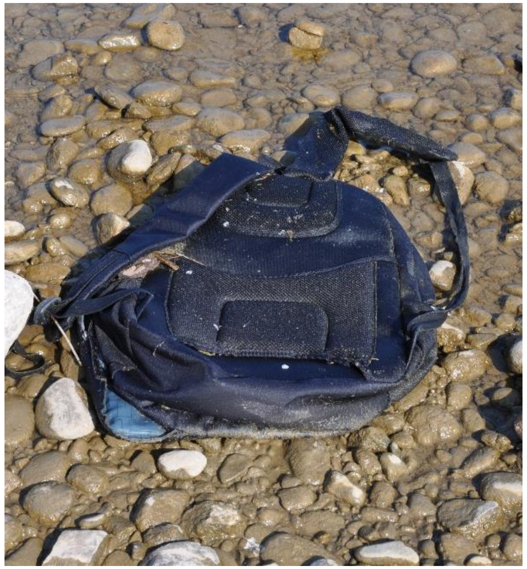

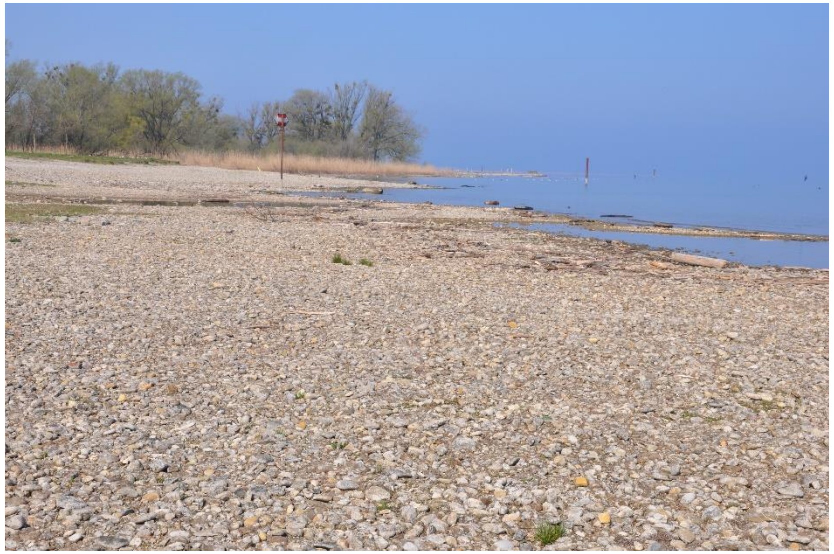

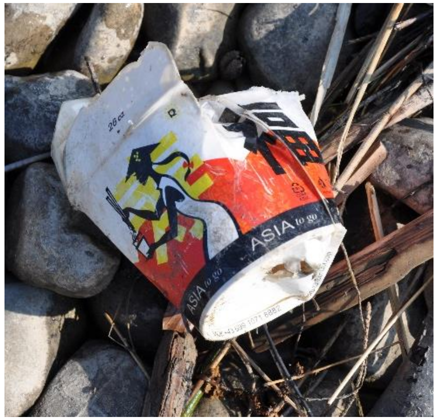

Lake Constance –Bregenz Seecamping (Austria) (beach #9)

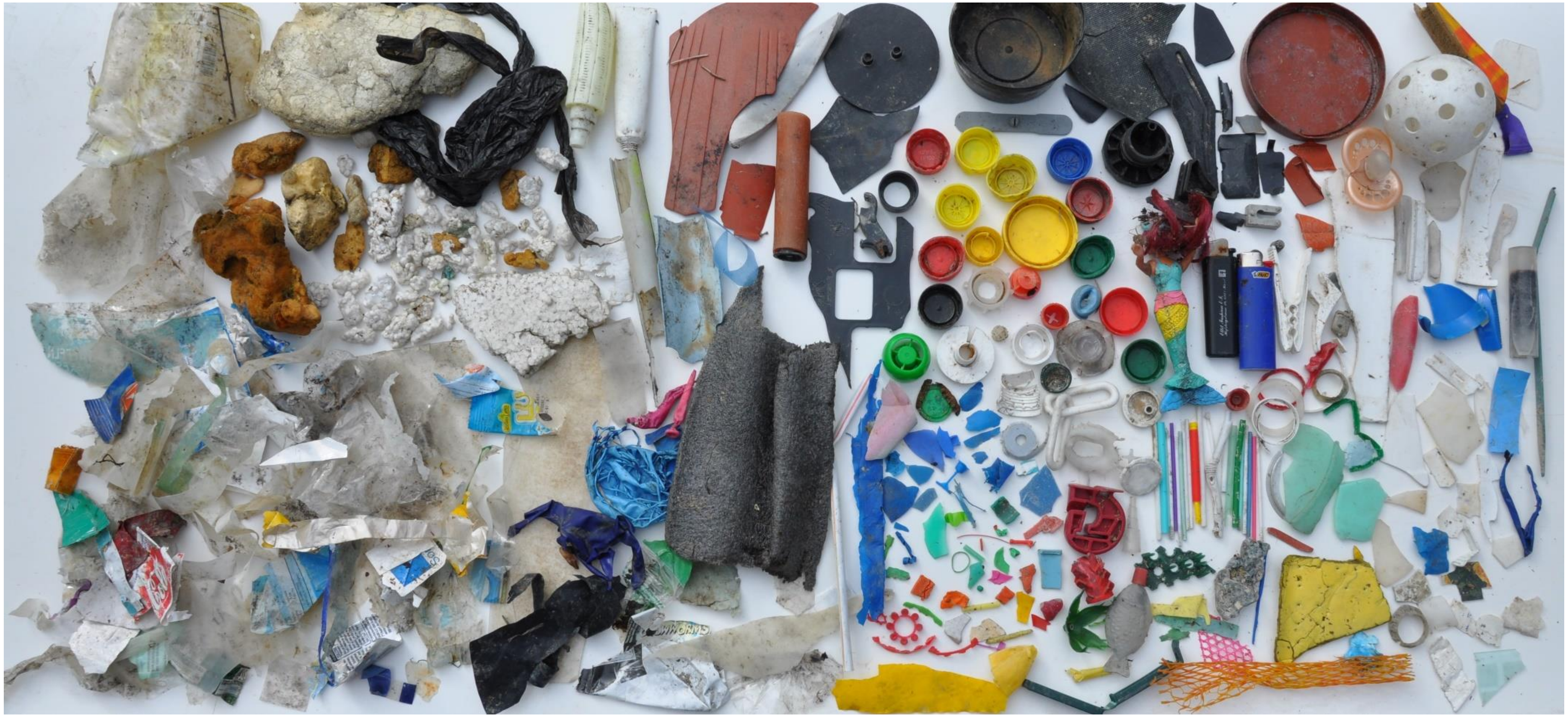

Lake Constance – Kesswill, Badeplatz (beach #10)

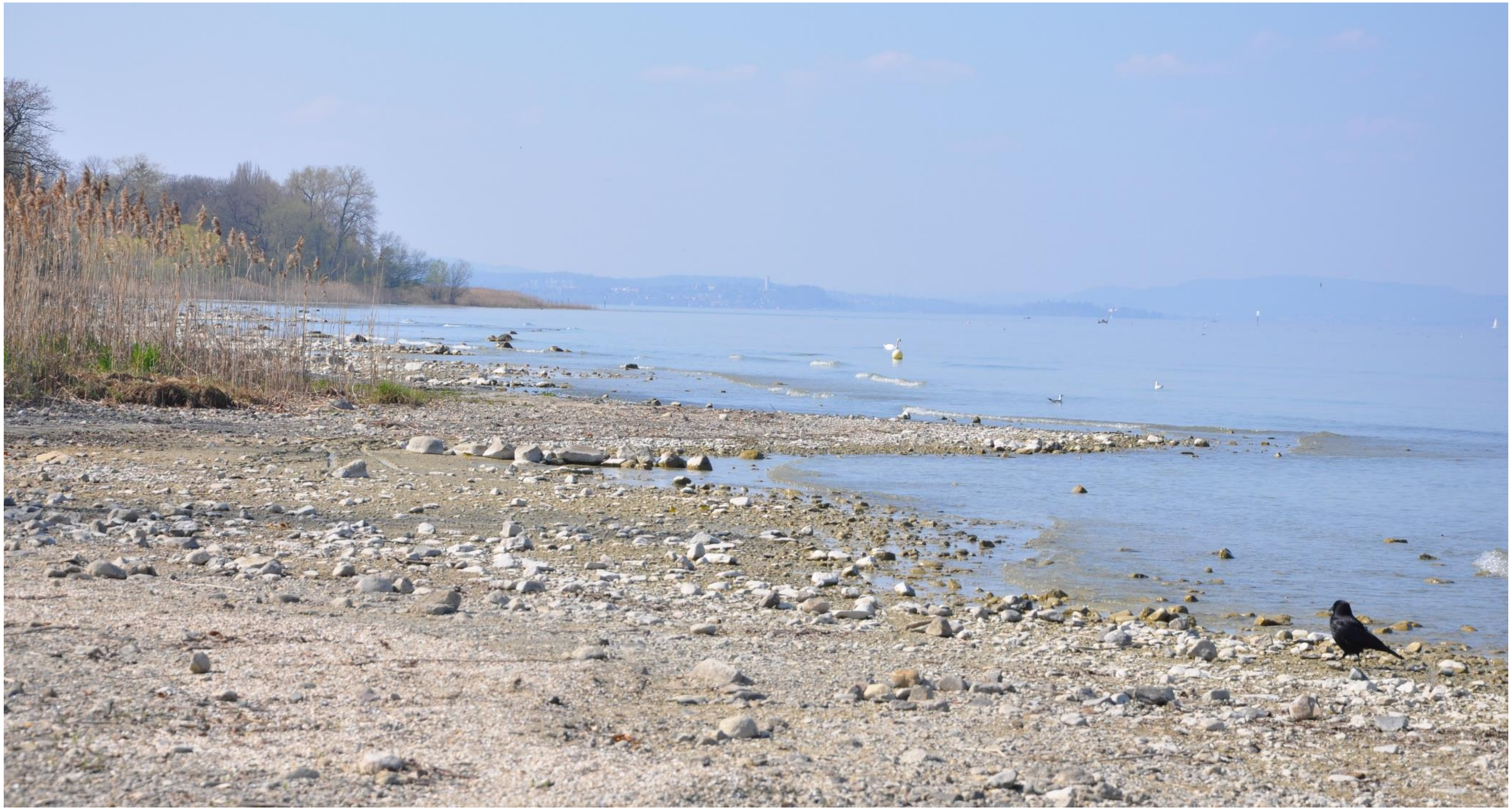

Lake Constance – Kesswill, Badeplatz (beach #10)

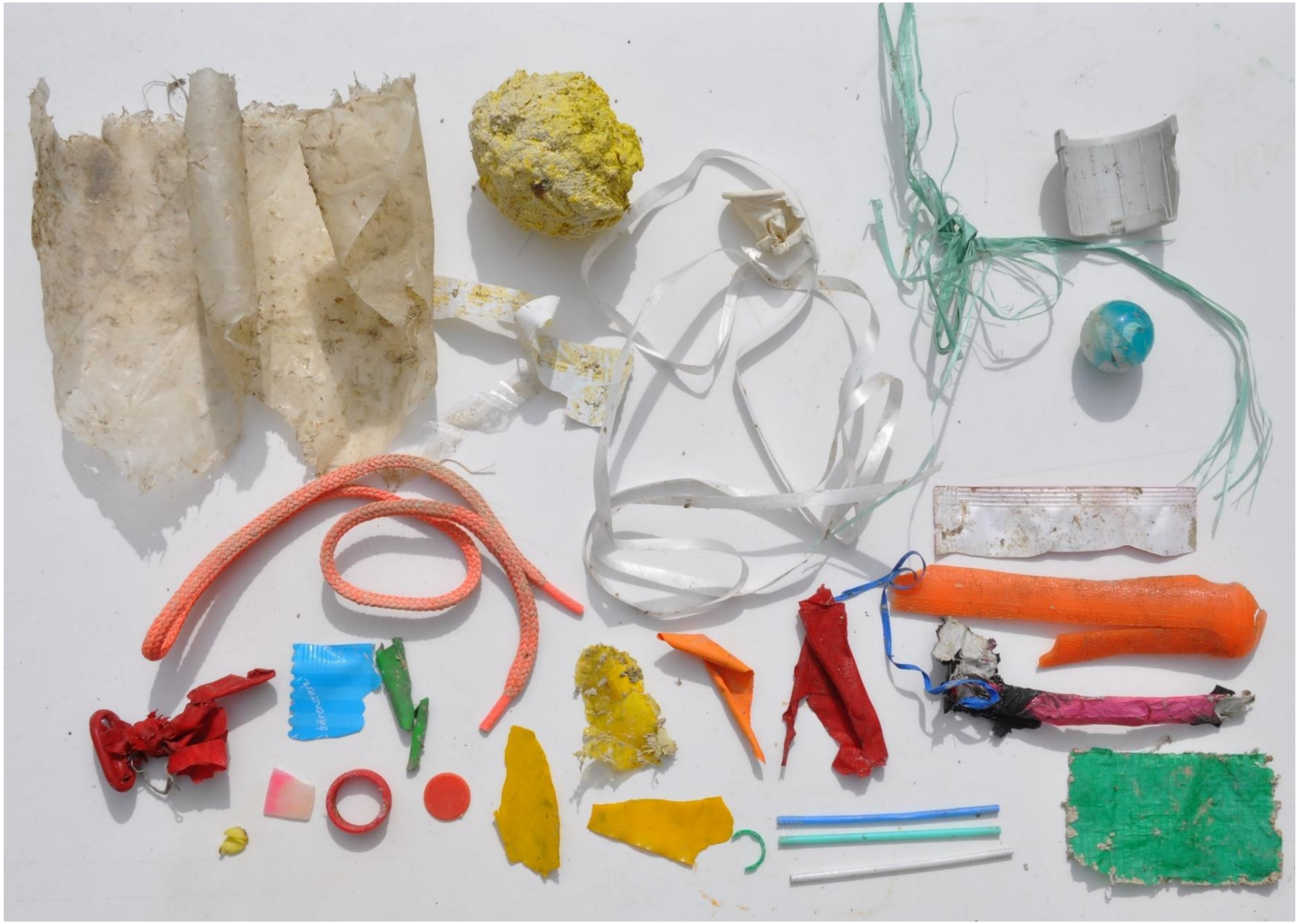

Lake Constance – Kressbronn, Strandbad (Germany) (beach #11)

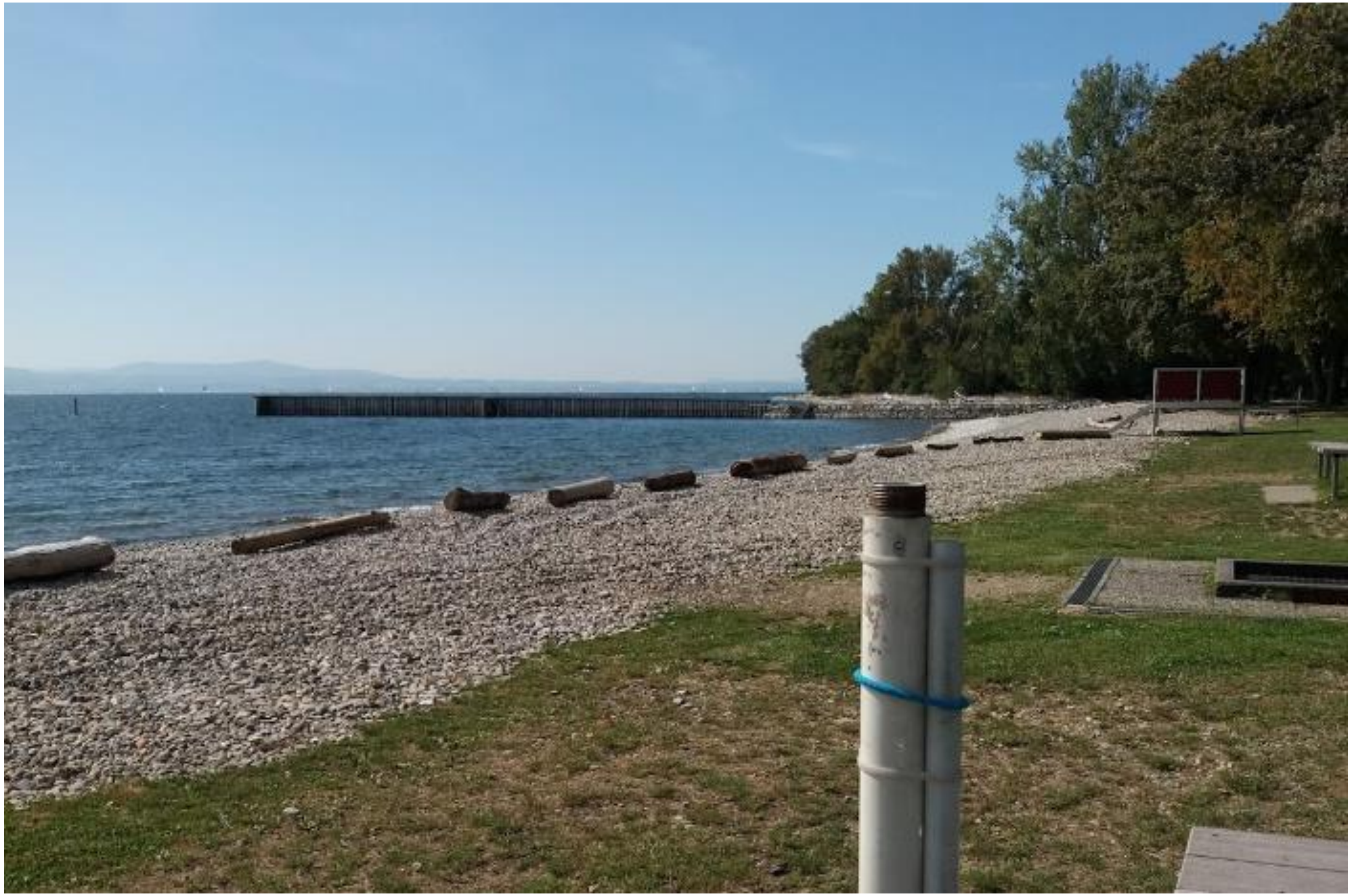

Lake Constance – Kressbronn, Strandbad (Germany) (beach #11). Two samples containing Hg highlighted in red (see text)

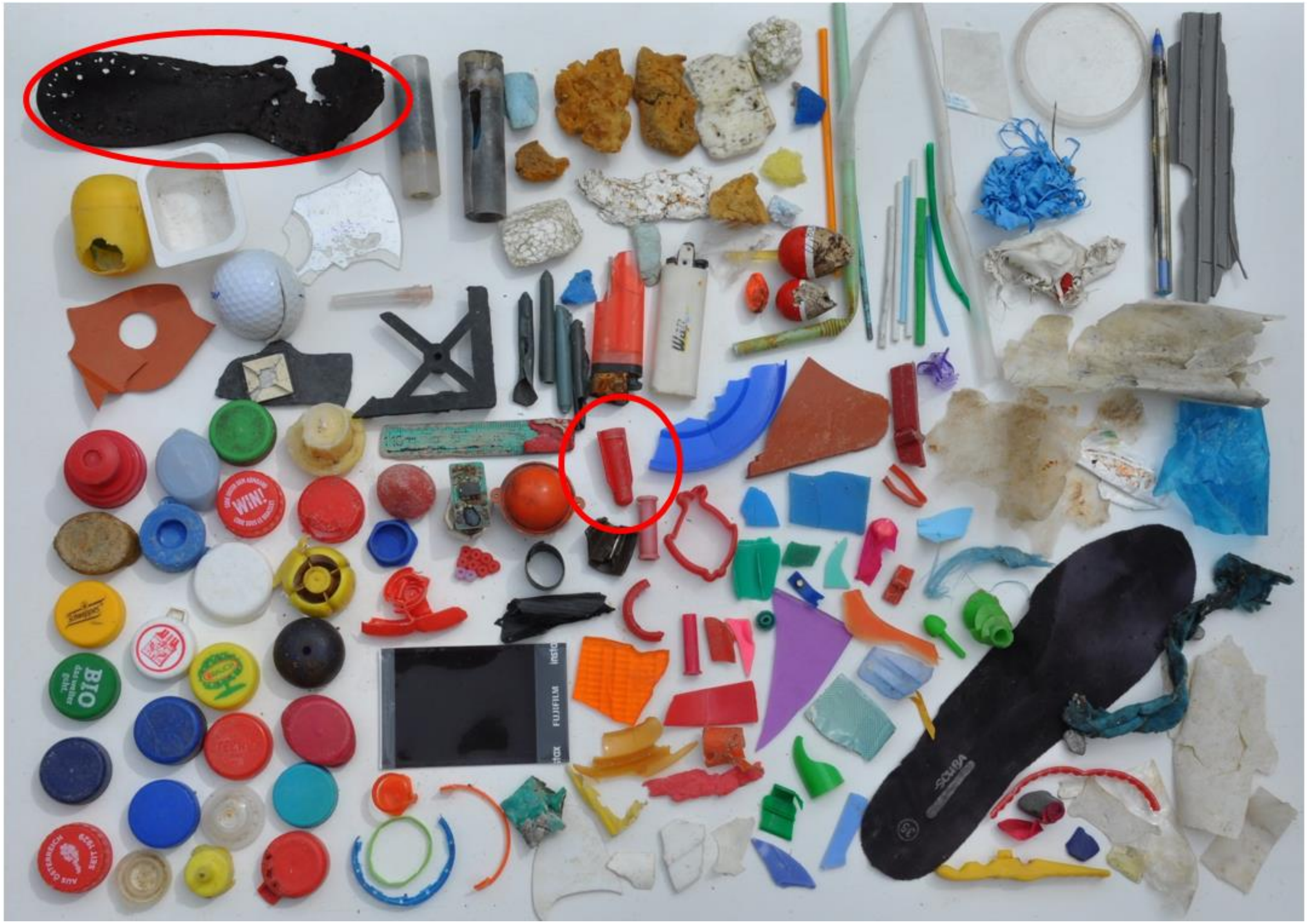

Lake Constance – Kreuzlingen, Seeburgplatz (beach #12)

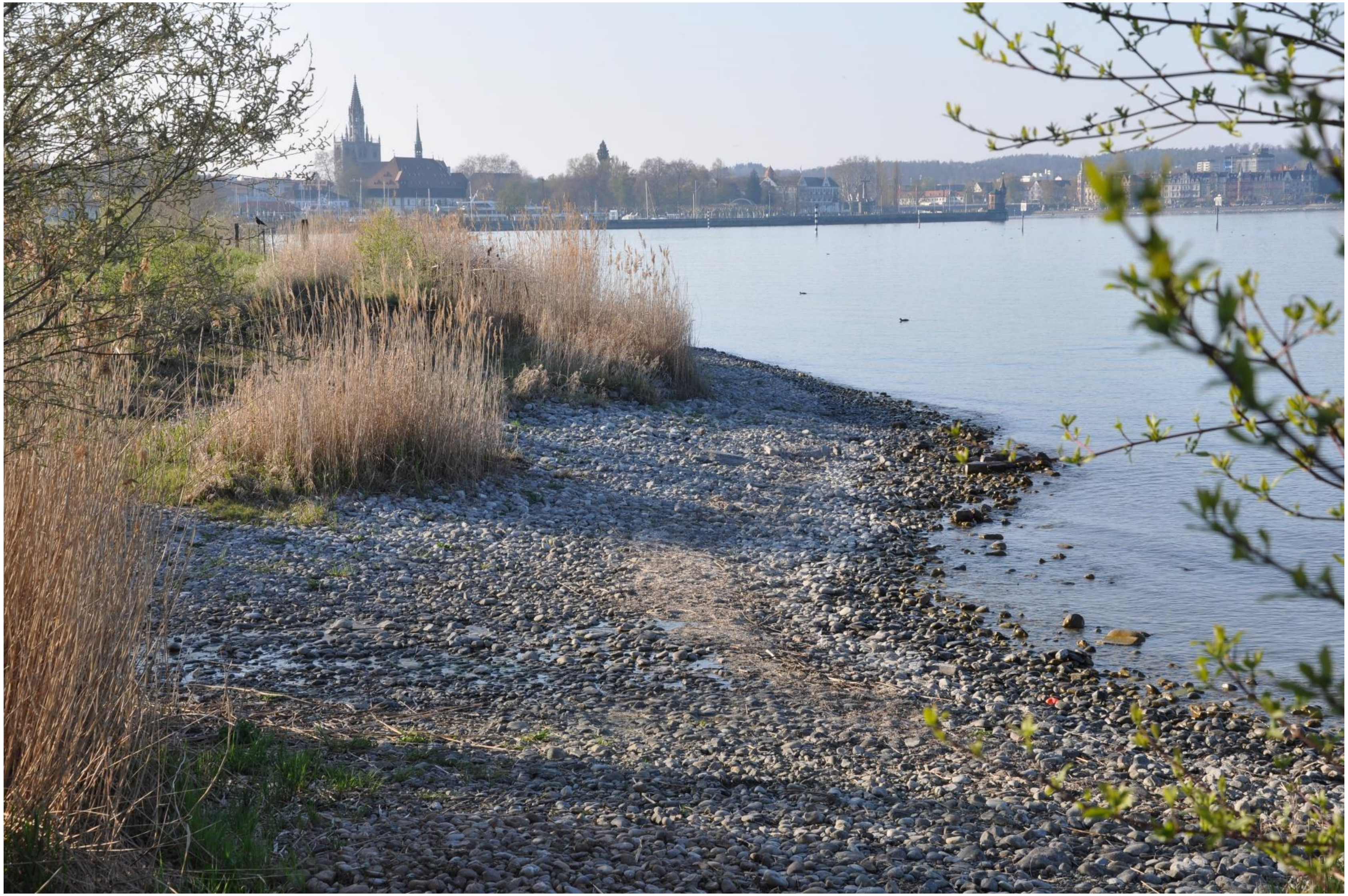

Lake Constance – Kreuzlingen, Seeburgplatz (beach #12)

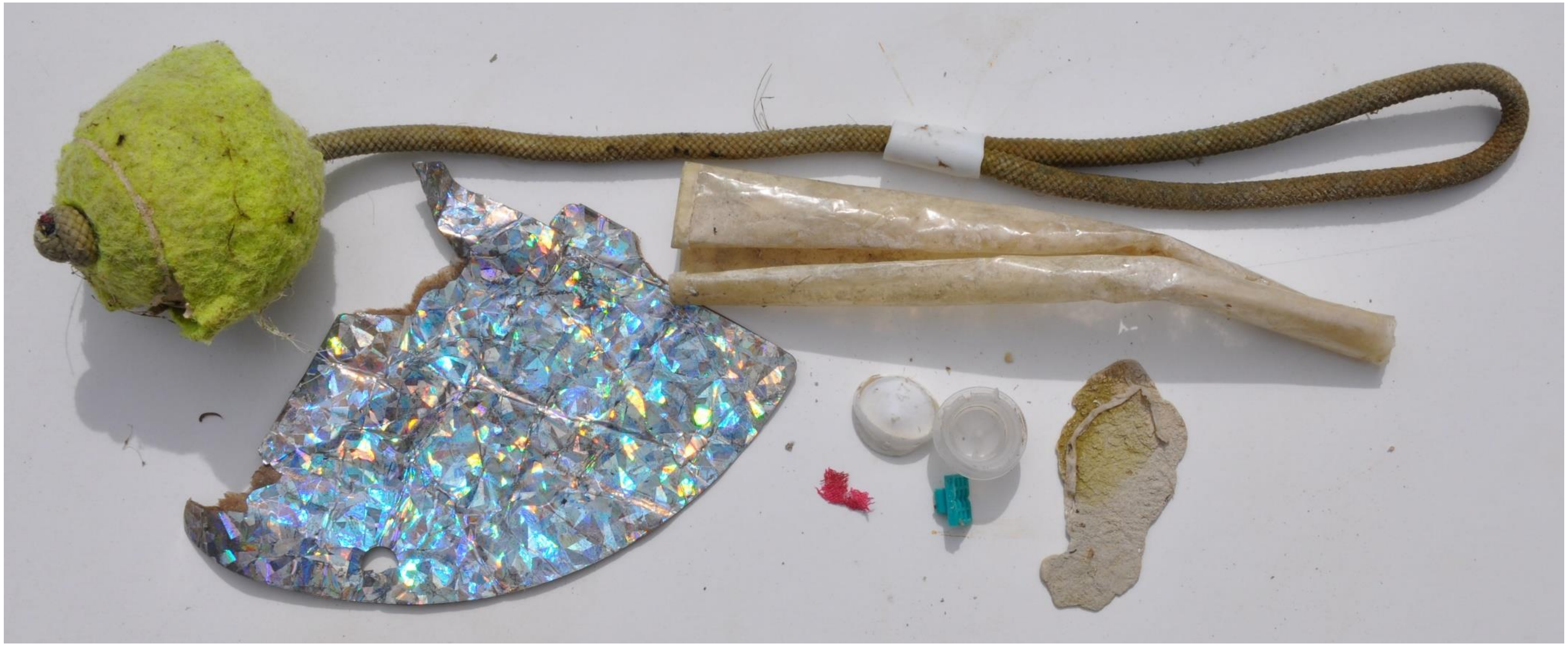

Lake Constance – Langenargen, Uferpark (Germany) (beach #13)

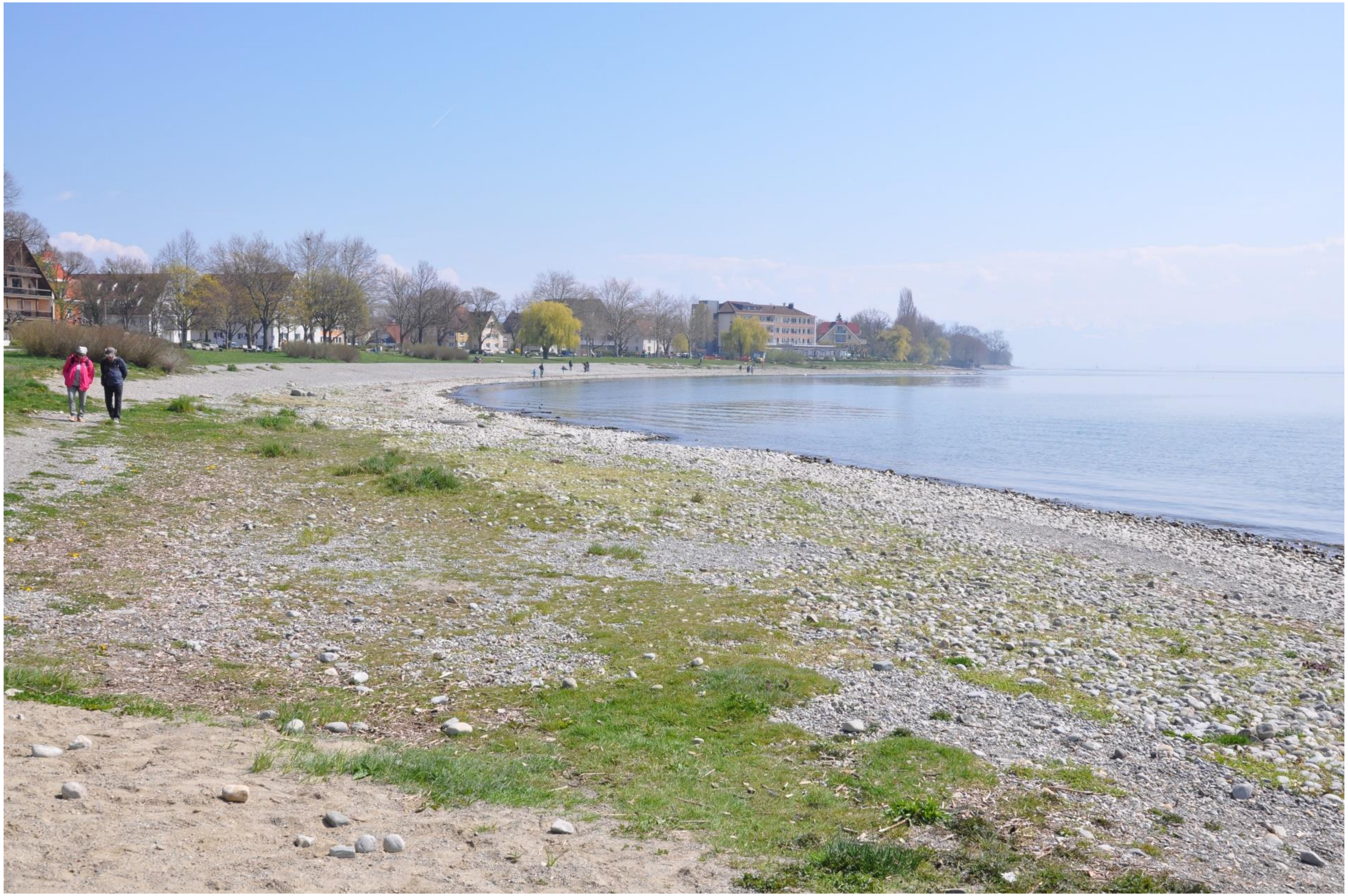

Lake Constance – Langenargen, Uferpark (Germany) (beach #13)

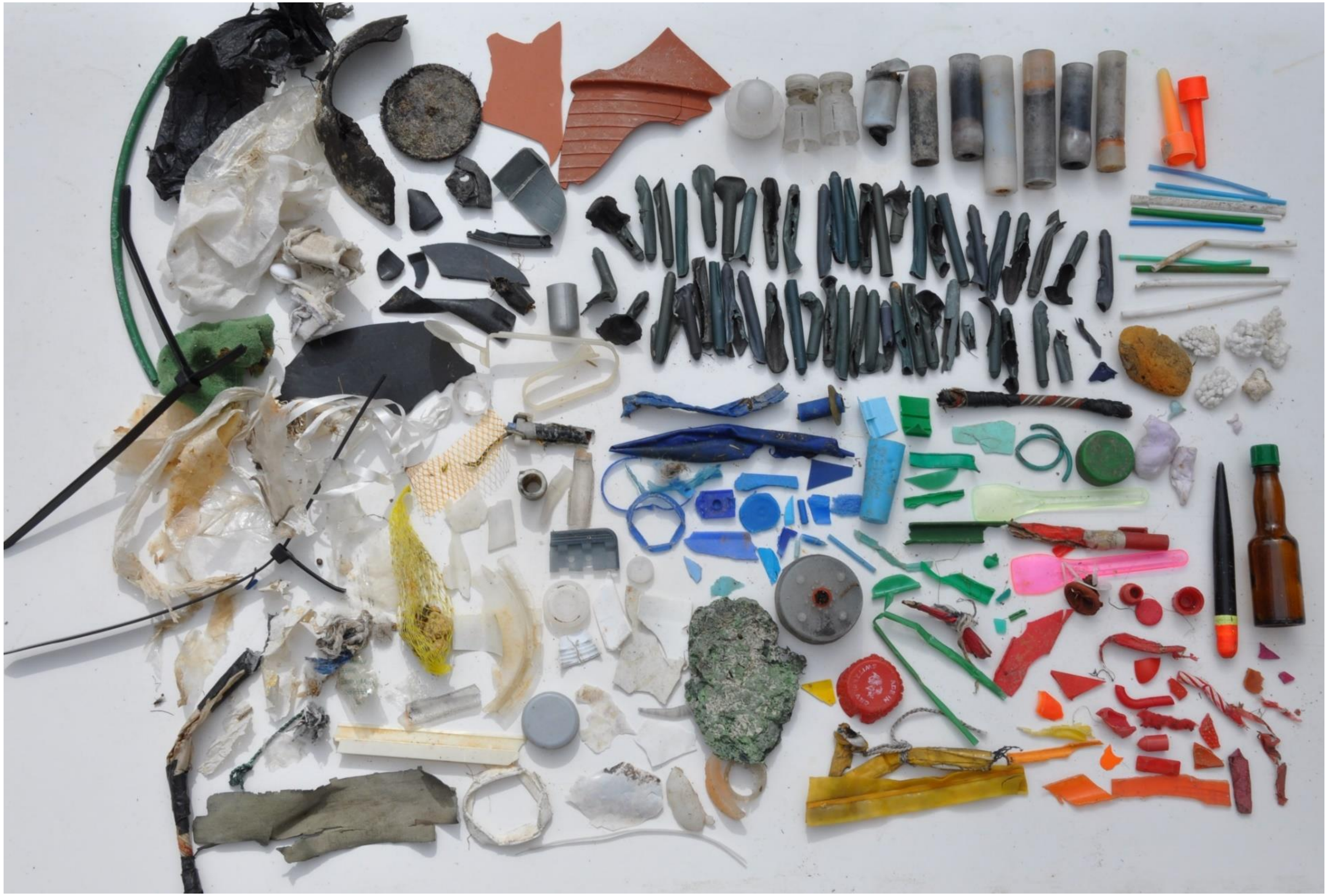

Lake Constance – Lindau, Reutiner Bucht (Germany) (beach #14)

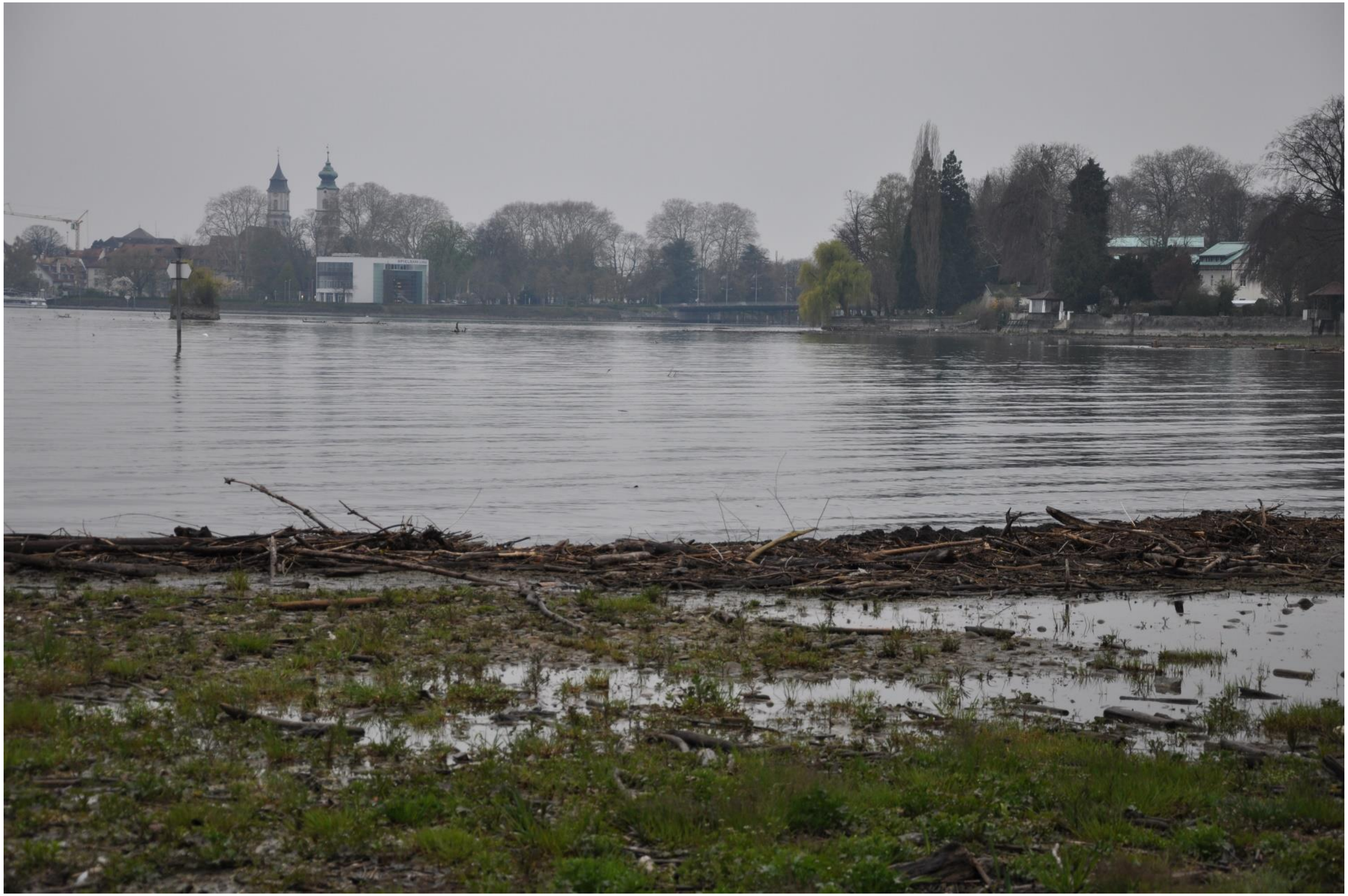

Lake Constance – Lindau, Reutiner Bucht (Germany) (beach #14)

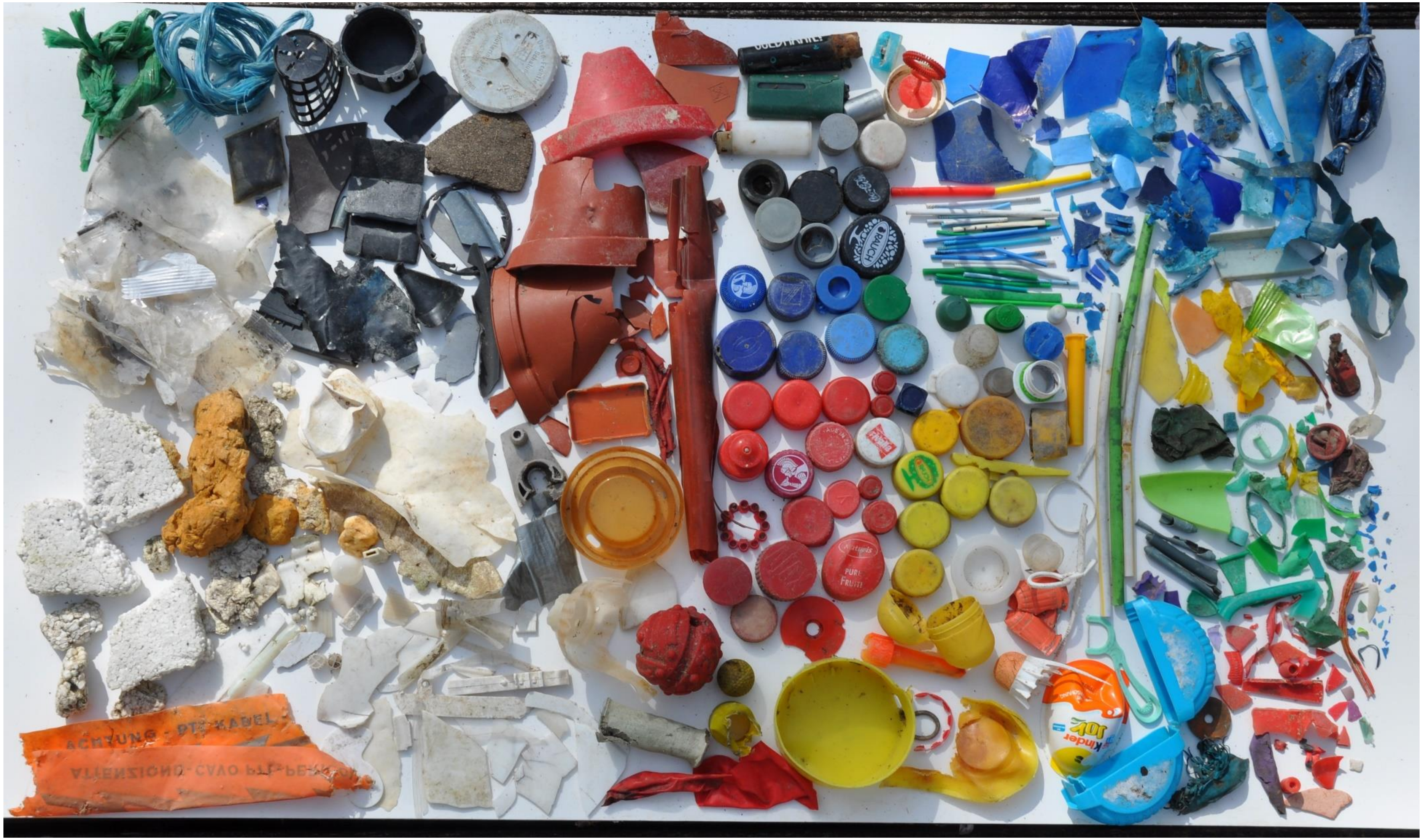

Lake Constance – Lindau, Eichwald Park (Germany) (beach #15)

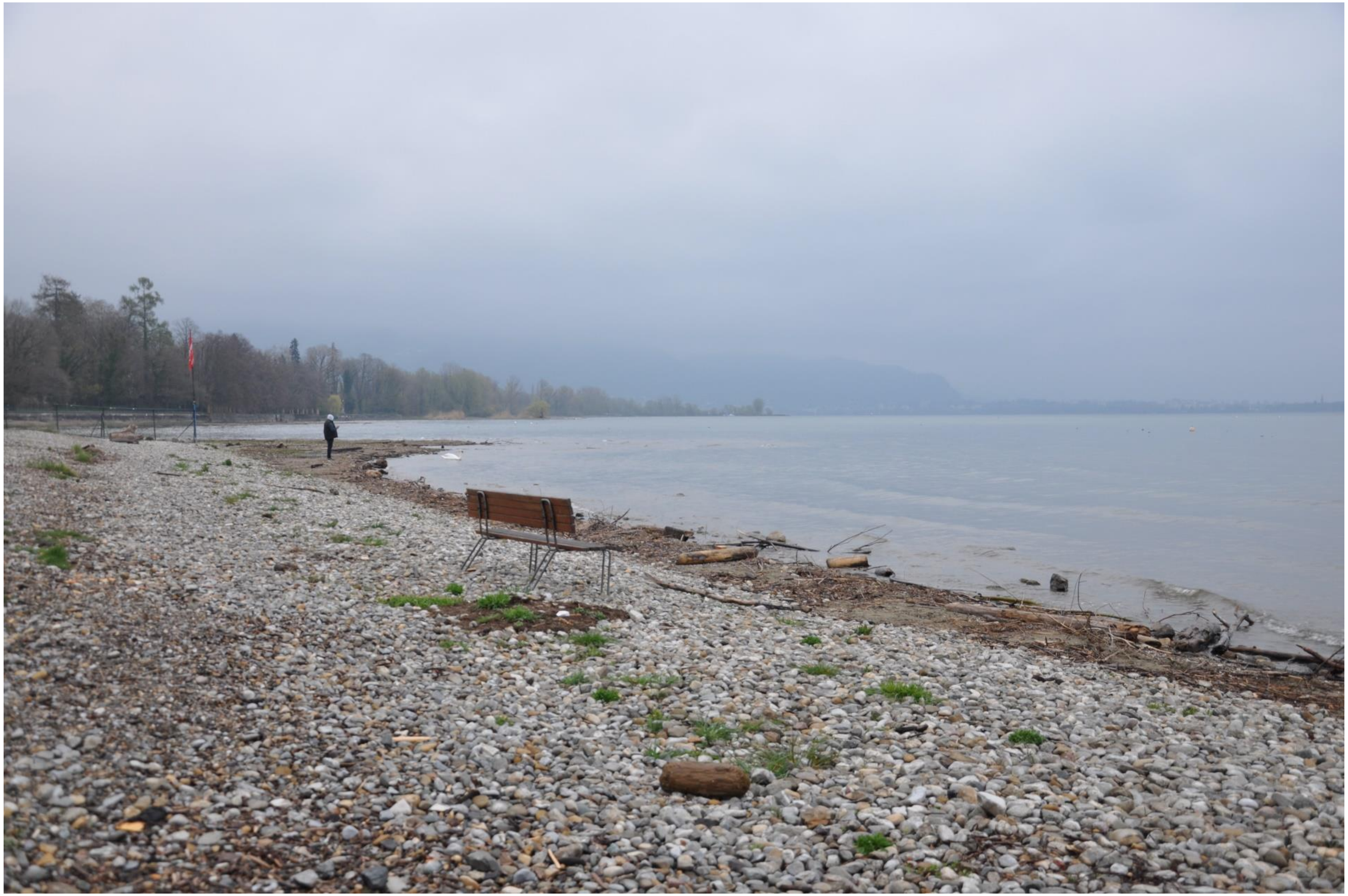

Lake Constance – Lindau, Eichwald Park (Germany) (beach #15)

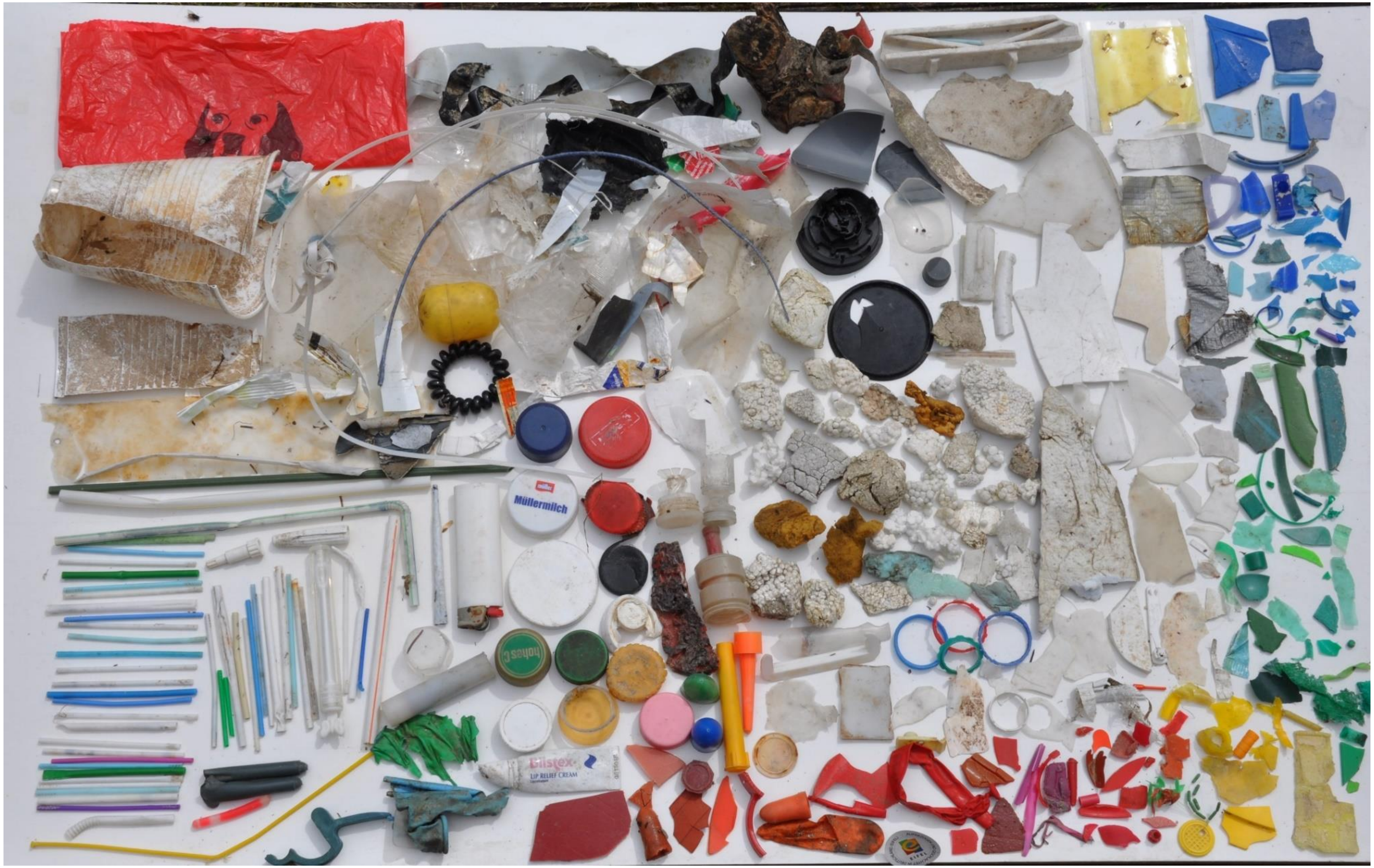

Lake Constance – Litzelstetten (Germany) (beach #16)

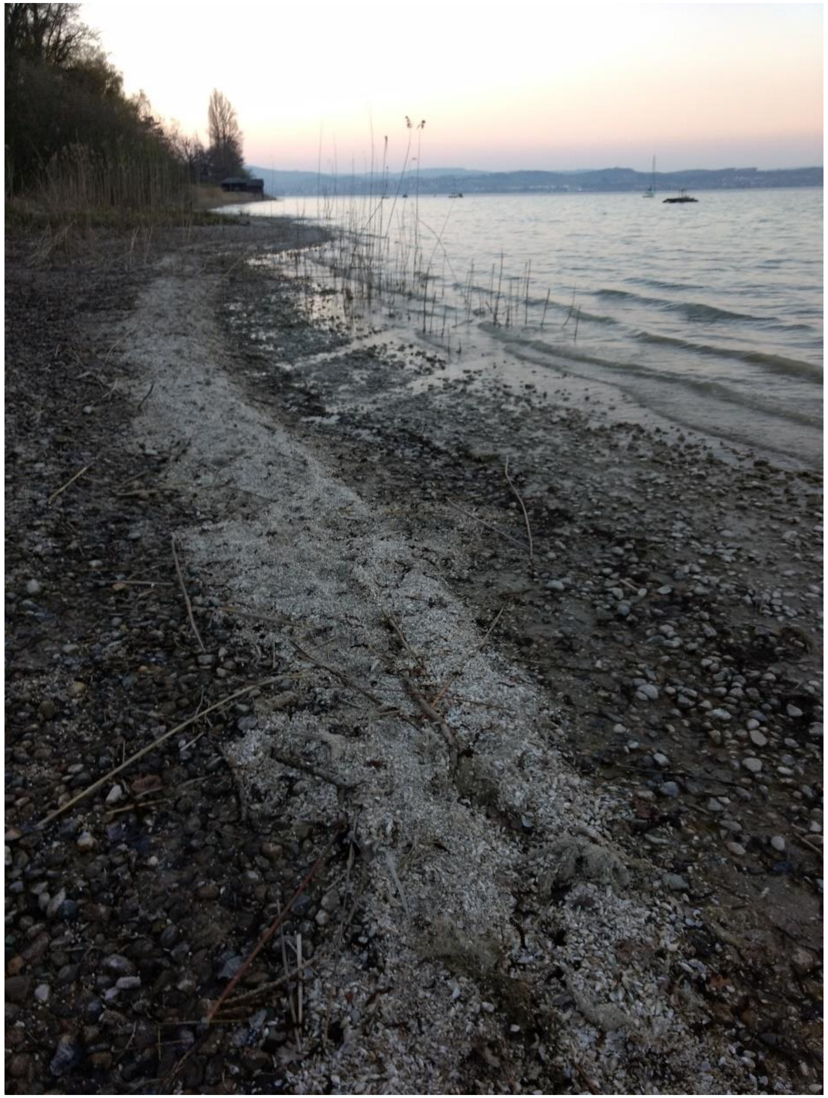

Lake Constance – Litzelstetten (Germany) (beach #16)

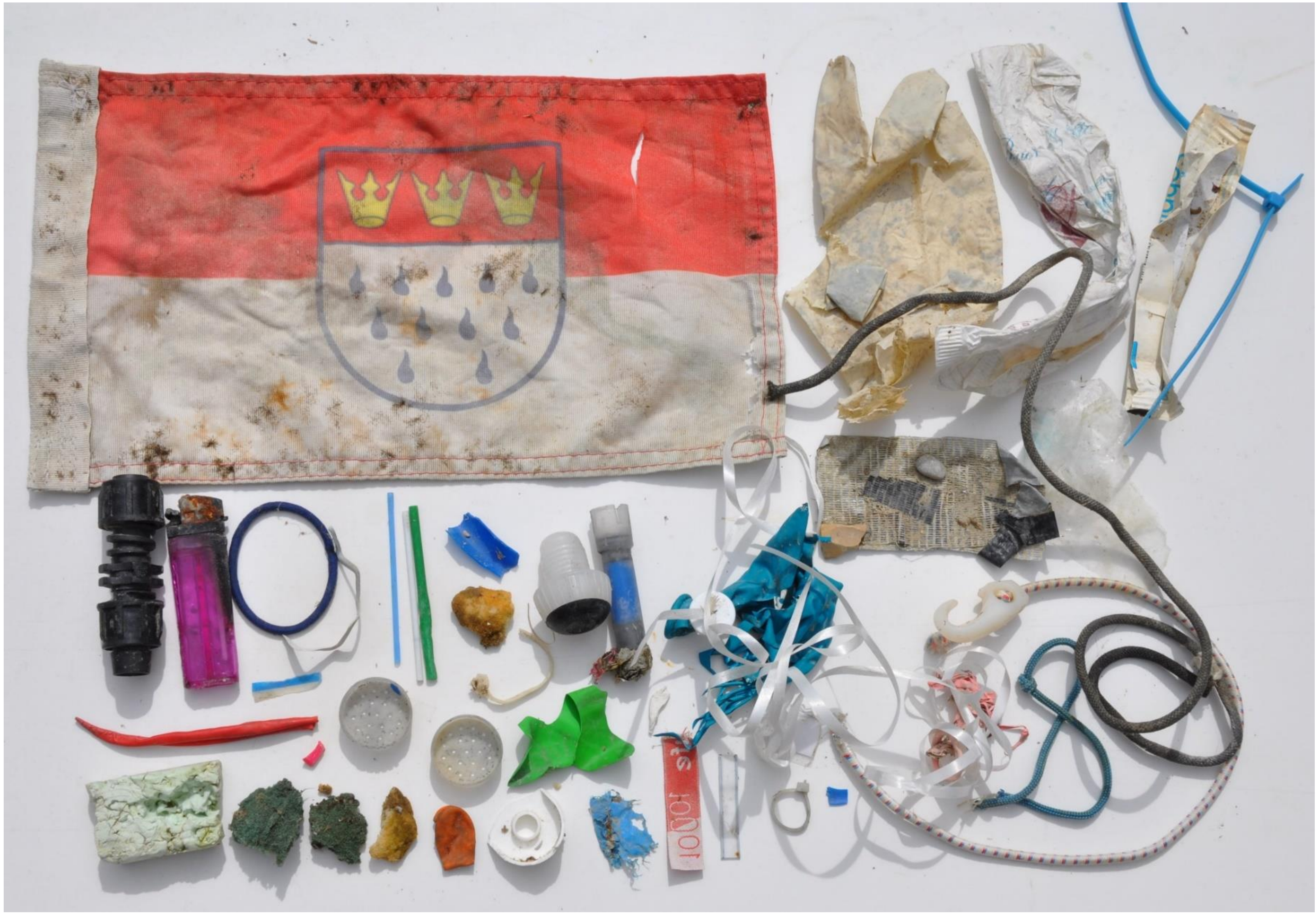

Lake Constance – Meersburg (Germany) (beach #17)

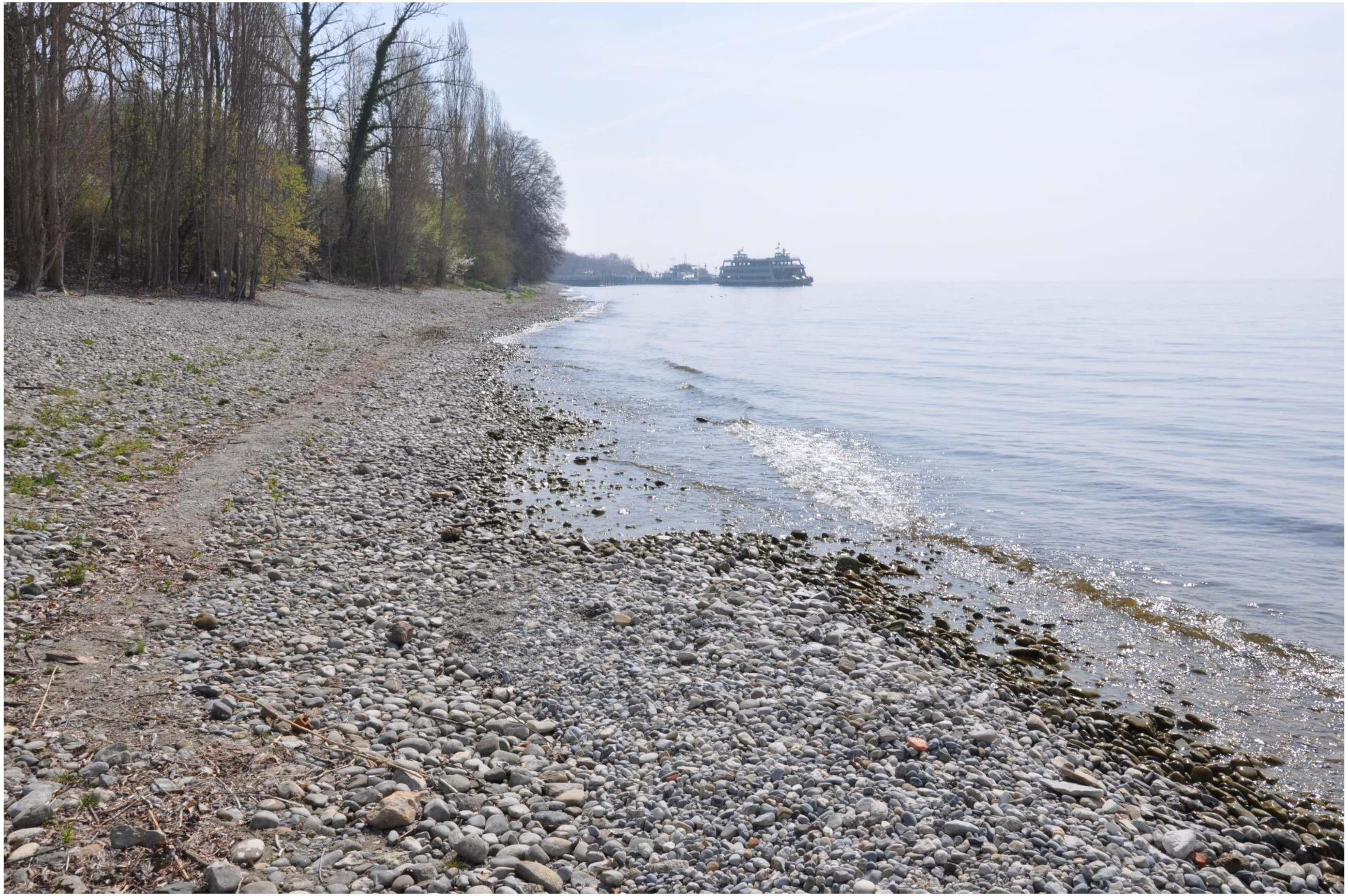

Lake Constance – Meersburg (Germany) (beach #17)

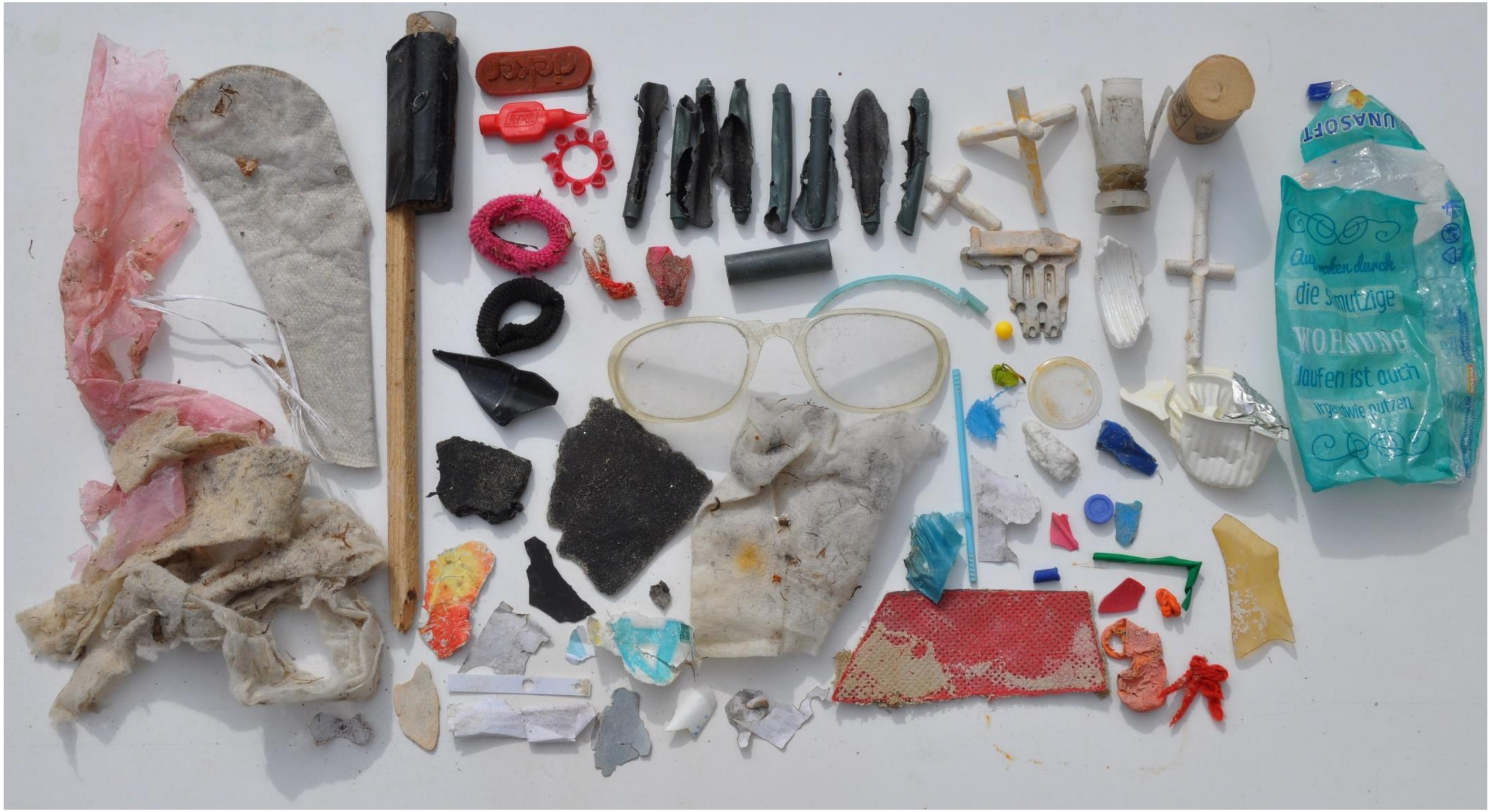

Lake Constance – Münsterlingen (beach #18)

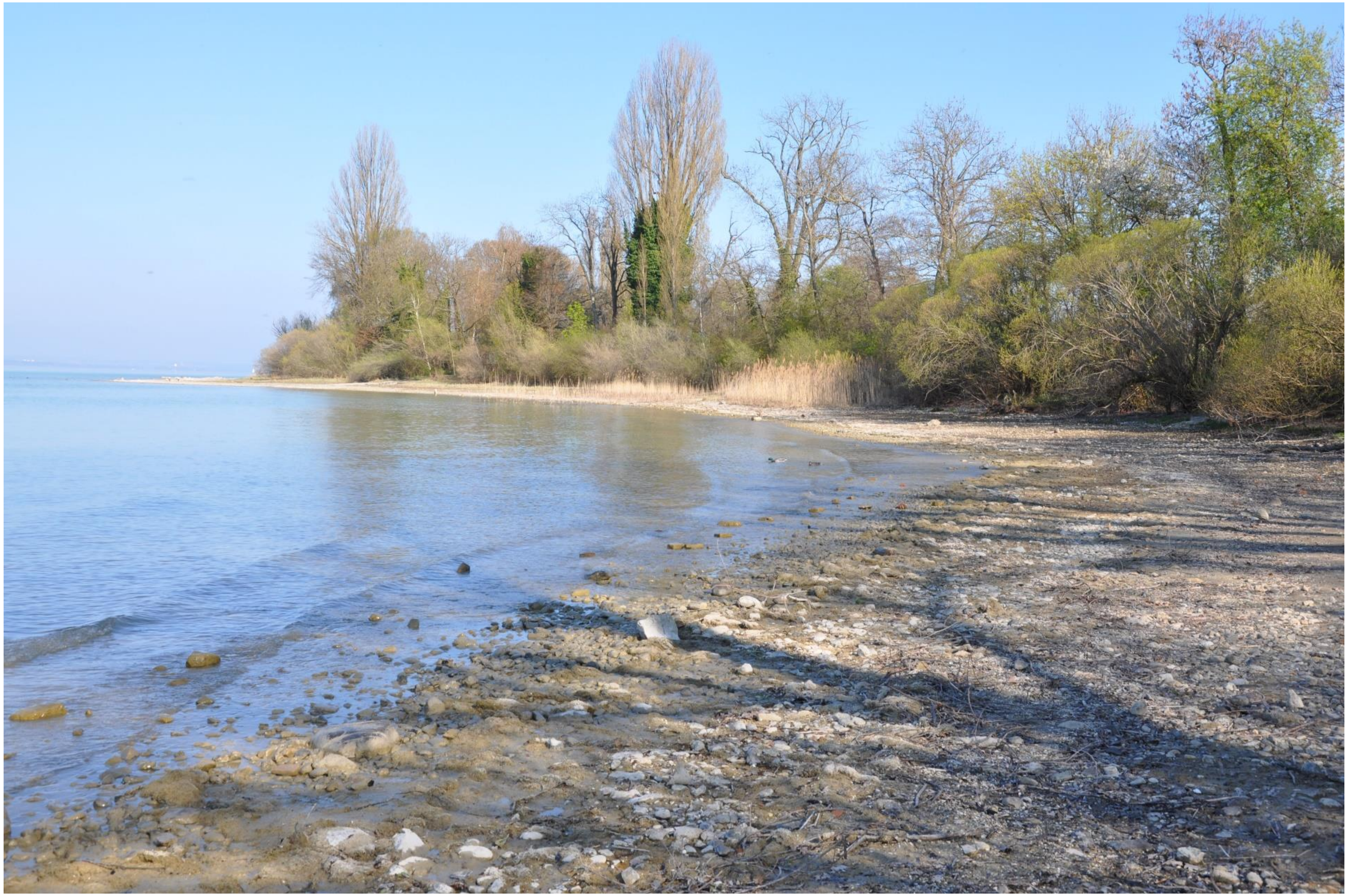

Lake Constance – Münsterlingen (beach #18)

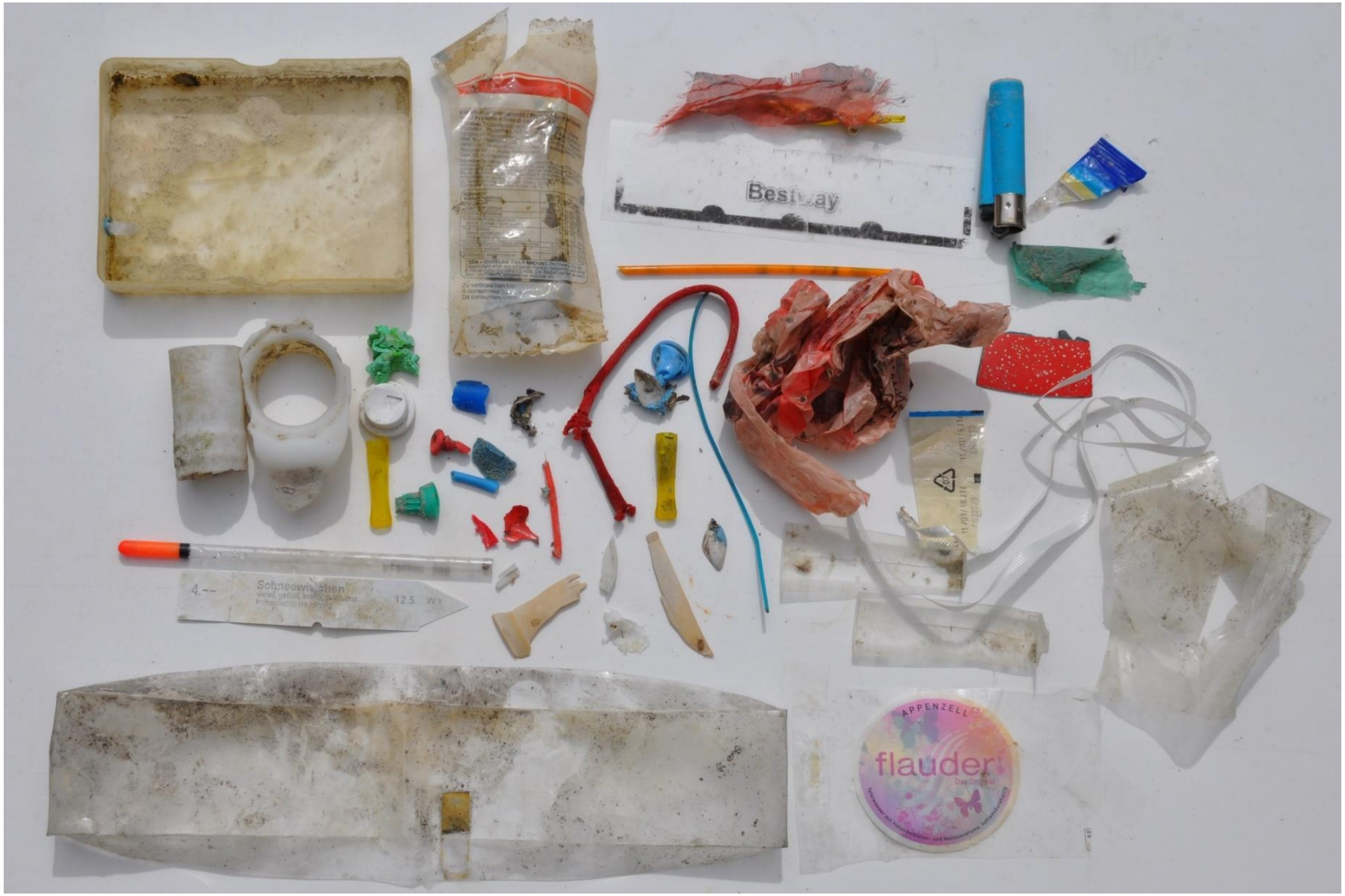

Lake Constance – Staad, Freibad Speck (beach #19)

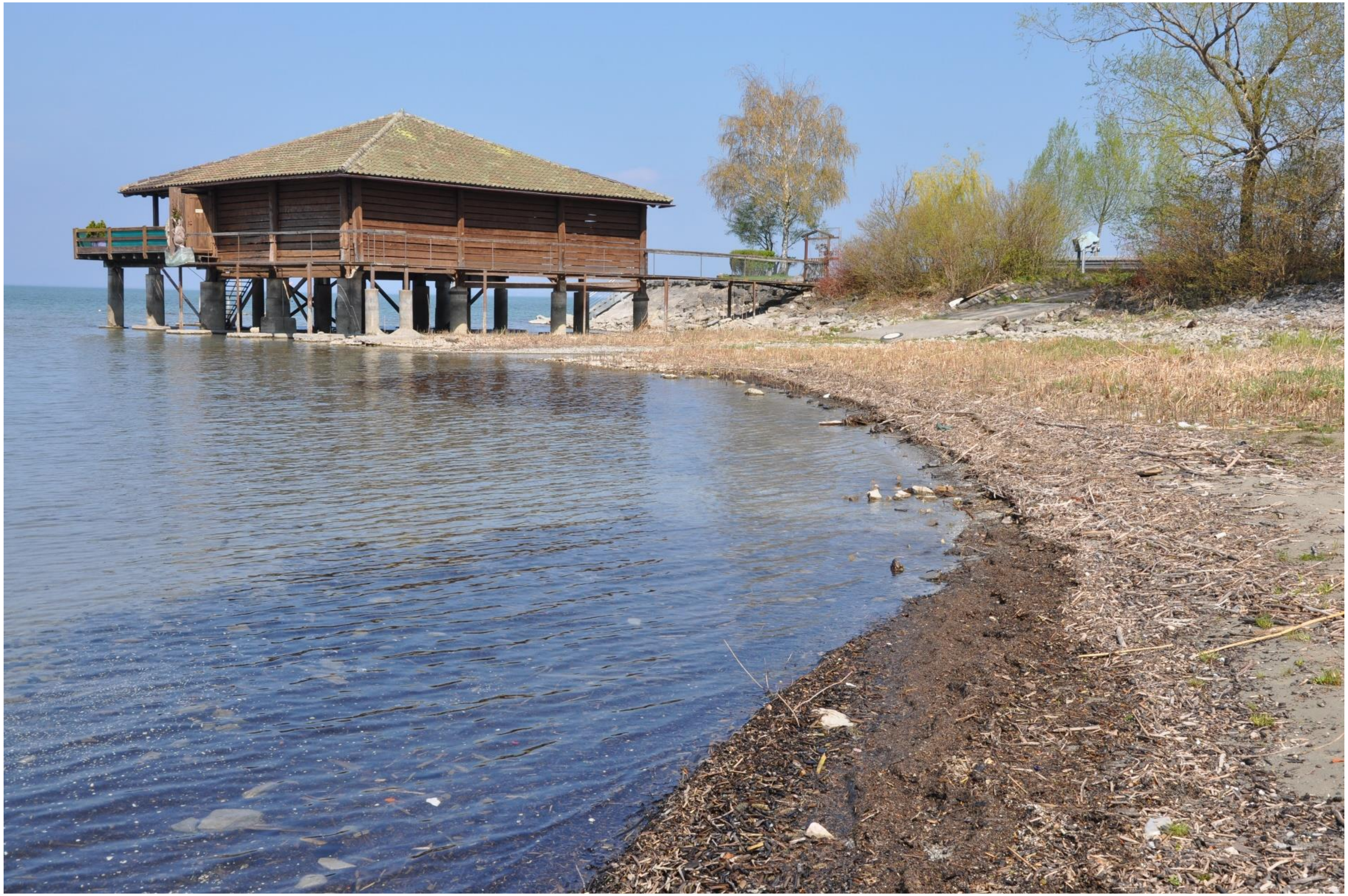

Lake Constance – Staad, Freibad Speck (beach #19)

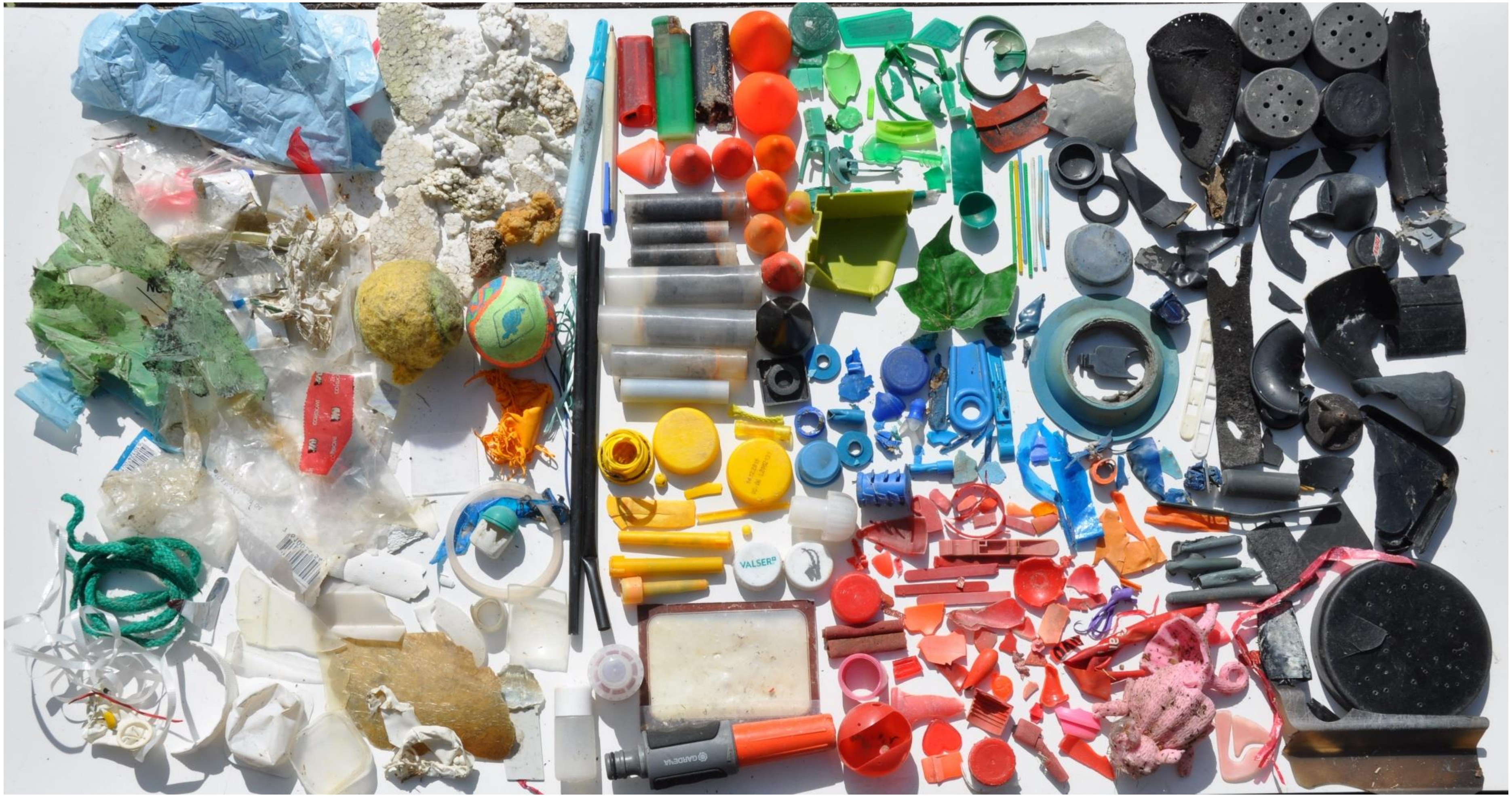

Lake Greifen

No pictures
