# Supplementary material for: What the presence of regulated chemical elements in beached lacustrine plastics can tell us: the case of Swiss lakes
Source: Environ Monit Assess. 2021 Oct 6;193(11):693. doi: 10.1007/s10661-021-09384-5 (PMC8492568; doi:10.1007/s10661-021-09384-5)

Supplementary Information -2

What the presence of regulated chemical elements in beached lacustrine plastics can tell us: the case of Swiss lakes

Montserrat Filella[1], Juan-Carlos Rodríguez-Murillo[2] and Andrew Turner[3]

[1]Department F.-A. Forel, University of Geneva, Boulevard Carl-Vogt 66, CH-1205 Geneva, Switzerland

[2]Museo Nacional de Ciencias Naturales, CSIC, Serrano 115 dpdo., E-28006 Madrid, Spain

[3]School of Geography, Earth and Environmental Sciences, Plymouth University, Drake Circus, Plymouth PL4 8AA, UK

Lake Morat – Vallamand(beach #21)

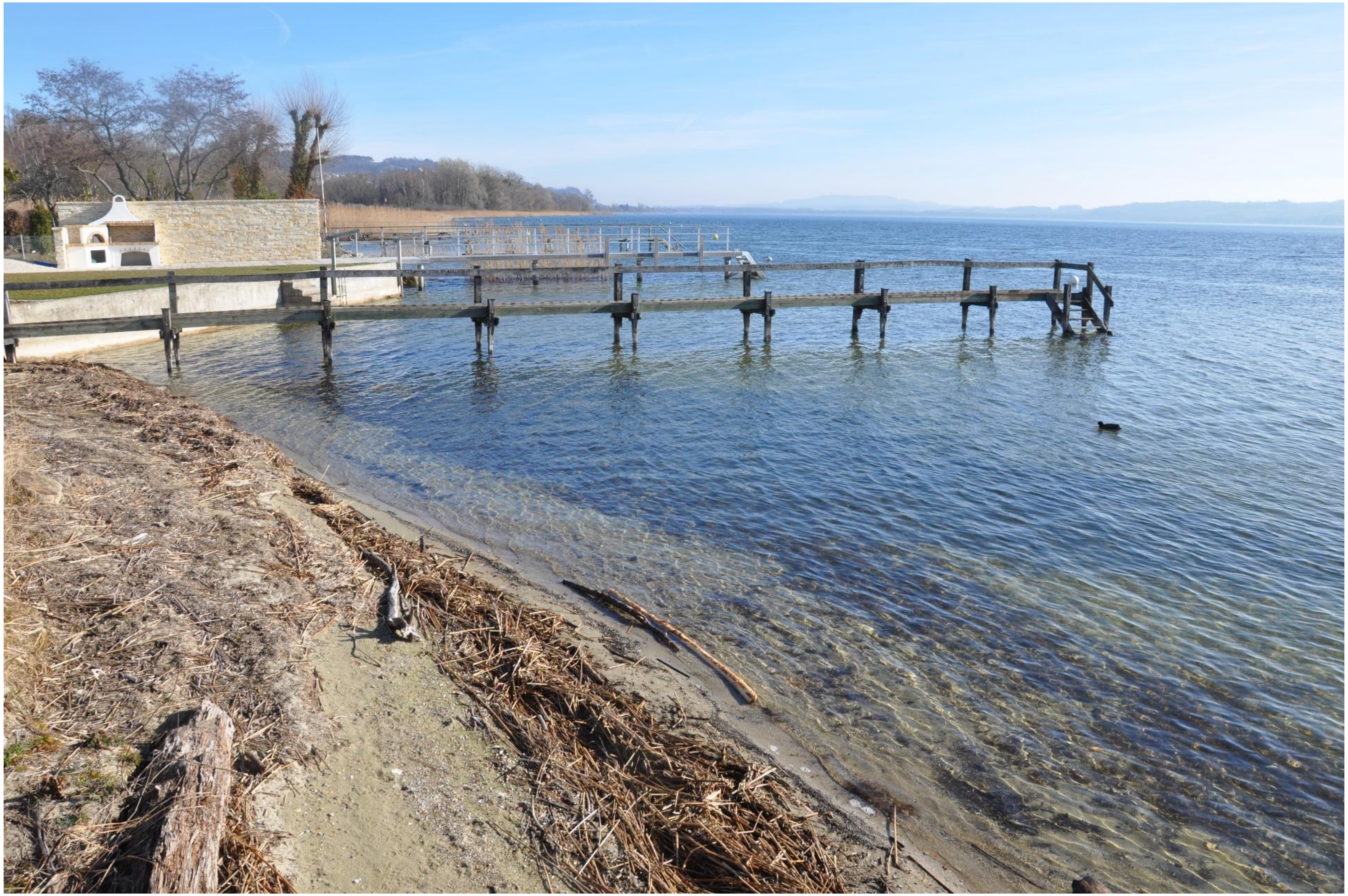

Lake Morat – Vallamand (beach #21)

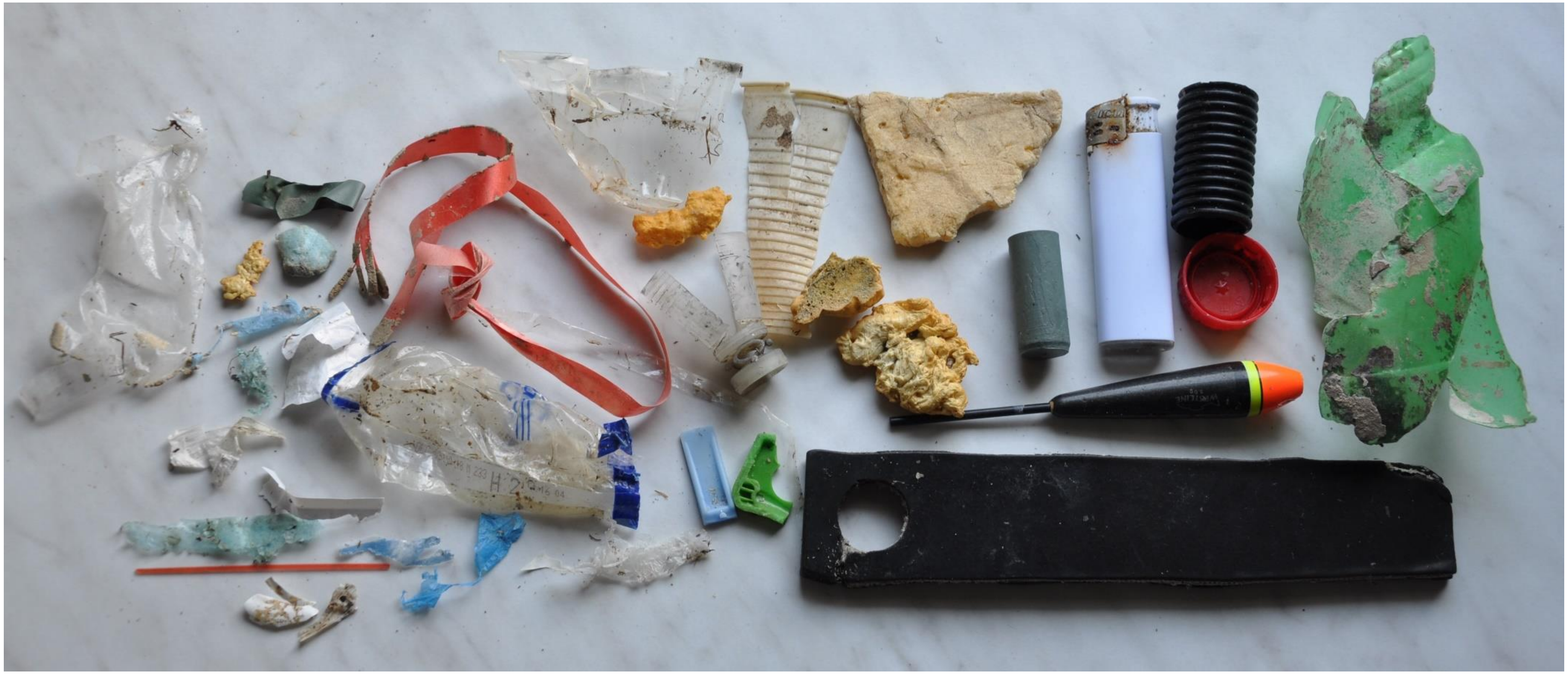

Lake Neuchâtel – Bevaix, Pointe du Grain (beach #22)

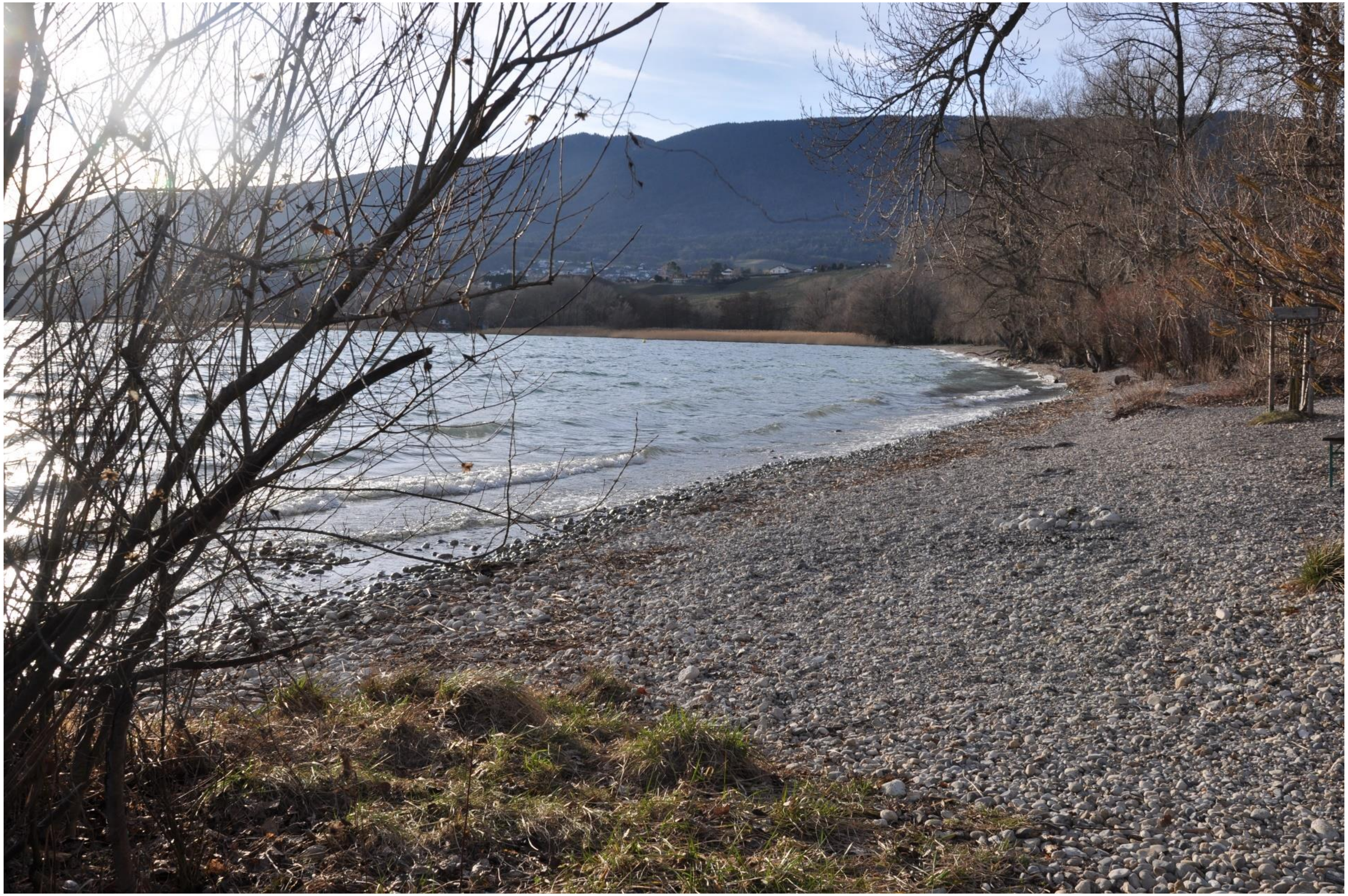

Lake Neuchâtel – Bevaix, Pointe du Grain (beach #22)

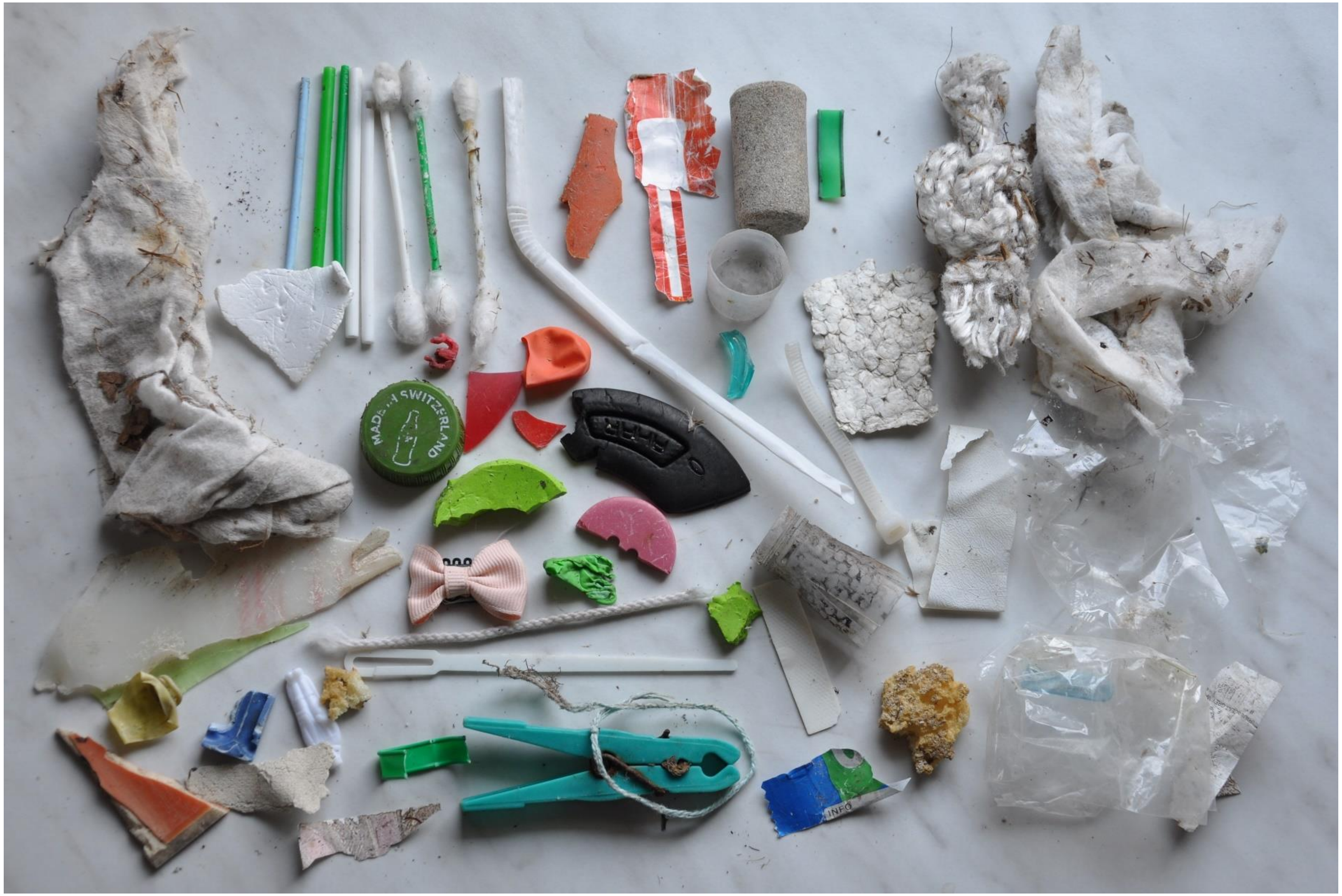

Lake Neuchâtel – Gletterens (beach #23)

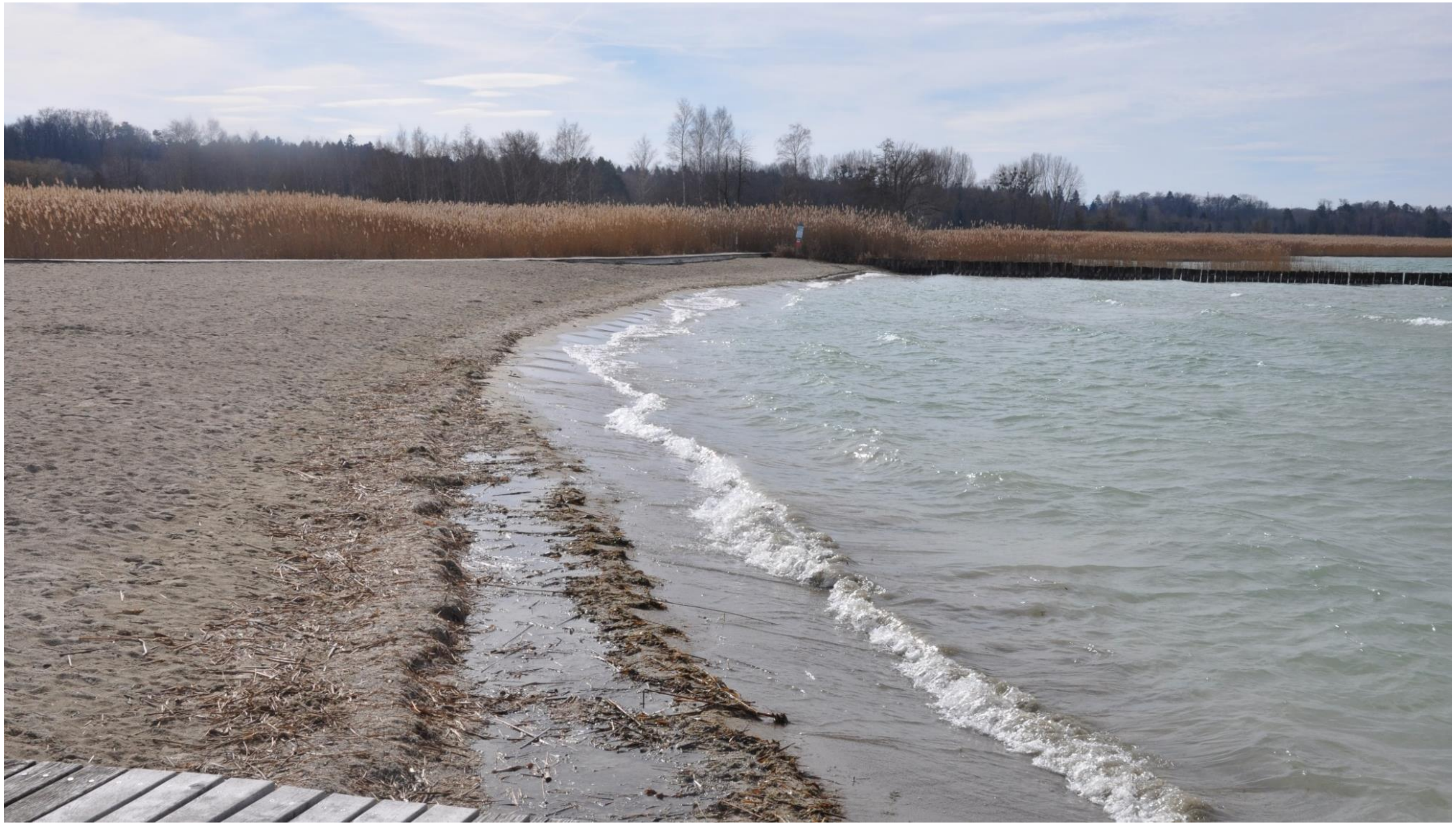

Lake Neuchâtel – Gletterens (beach #23)

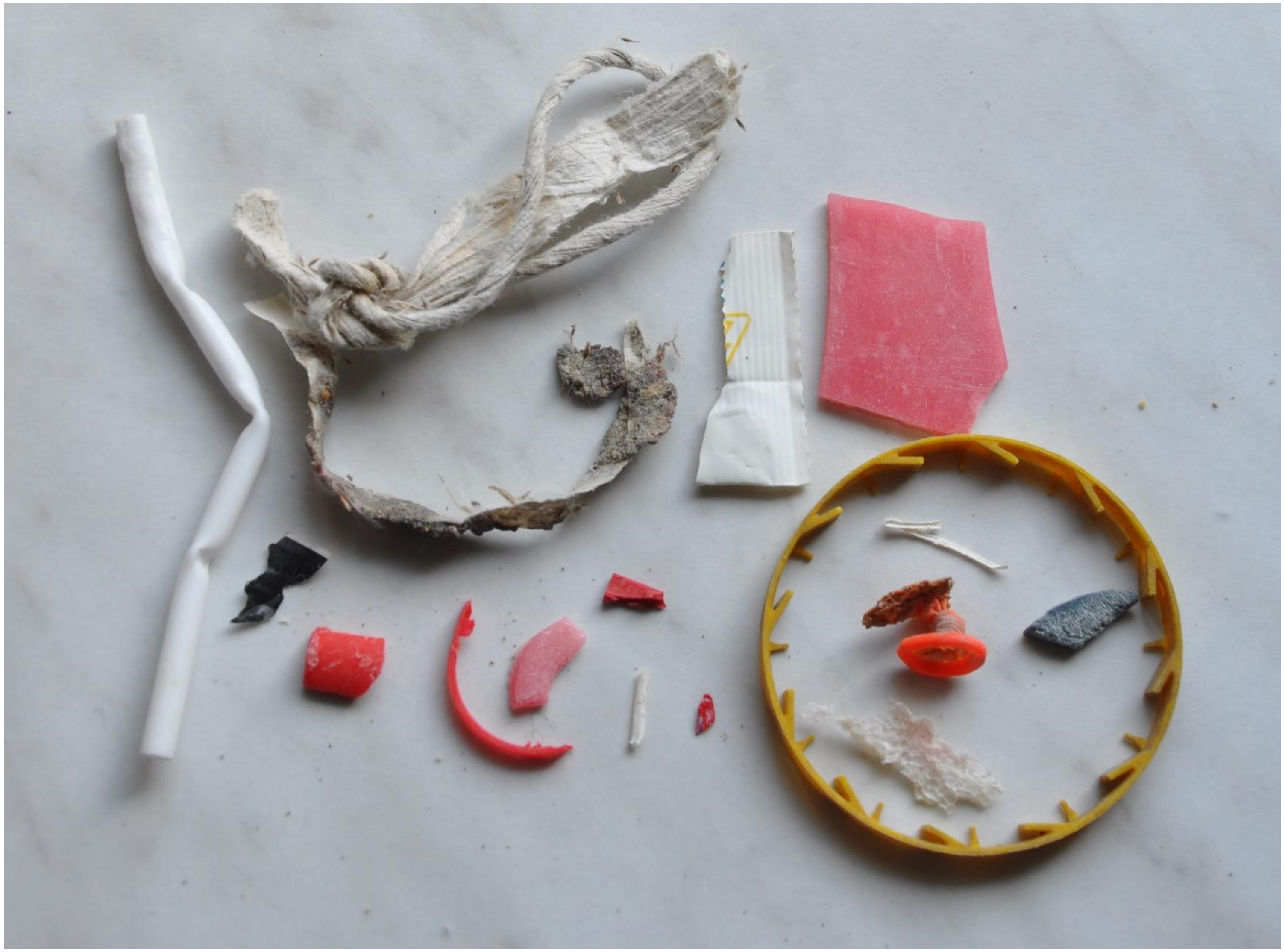

Lake Neuchâtel – Ivonand (beach #24)

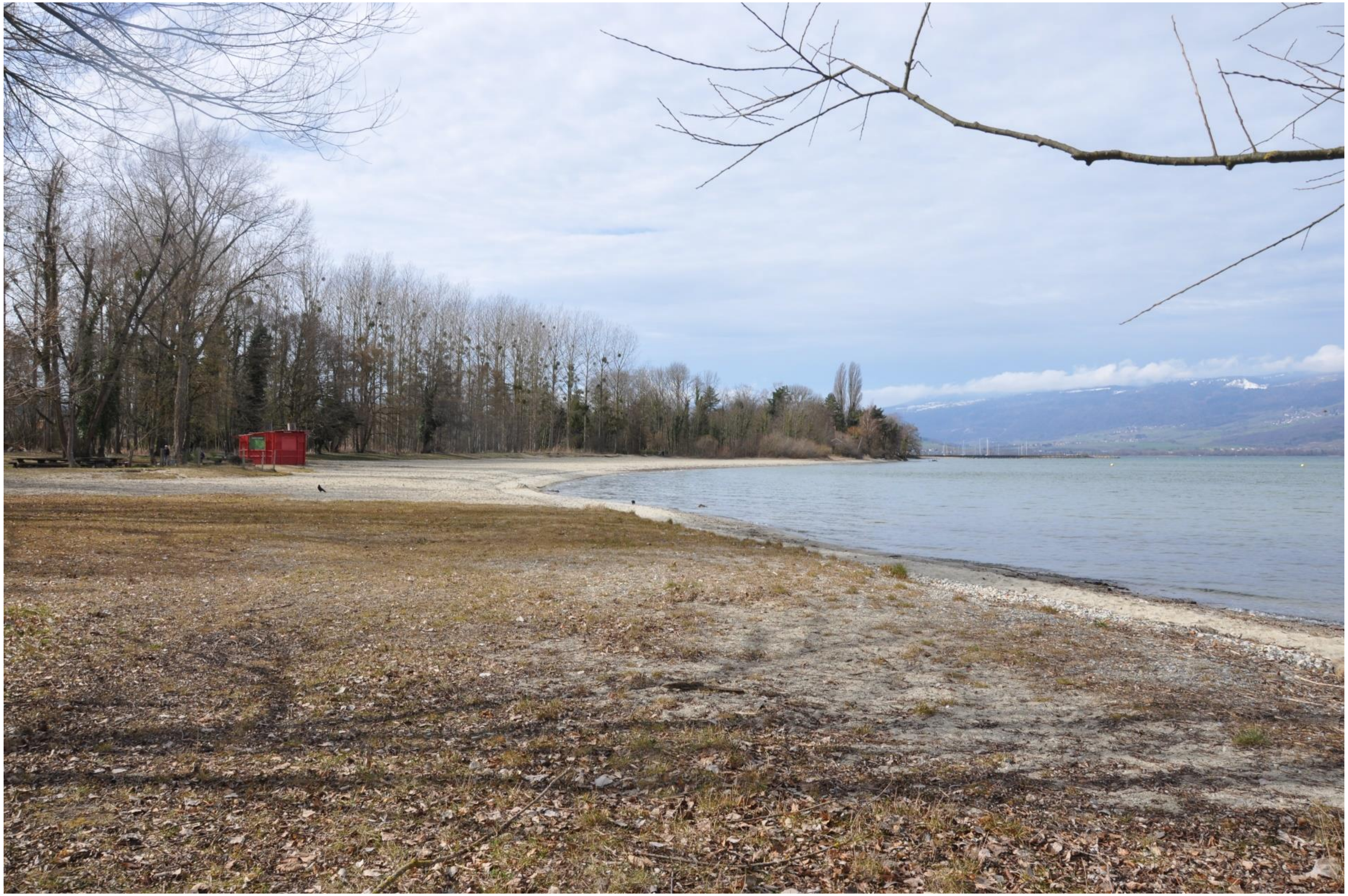

Lake Neuchâtel – Ivonand (beach #24)

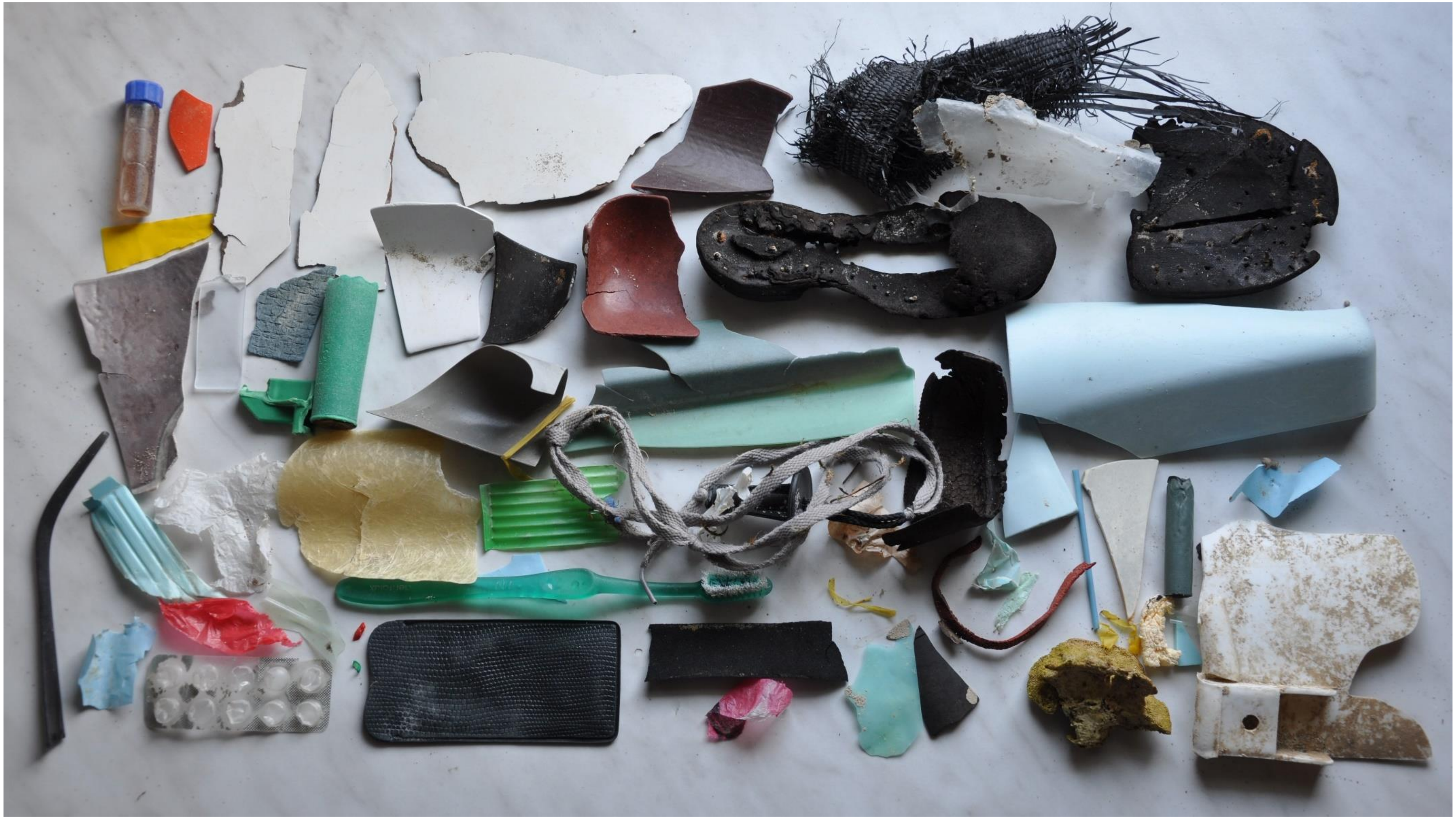

Lake Neuchâtel – Vaumarcus A (beach #25)

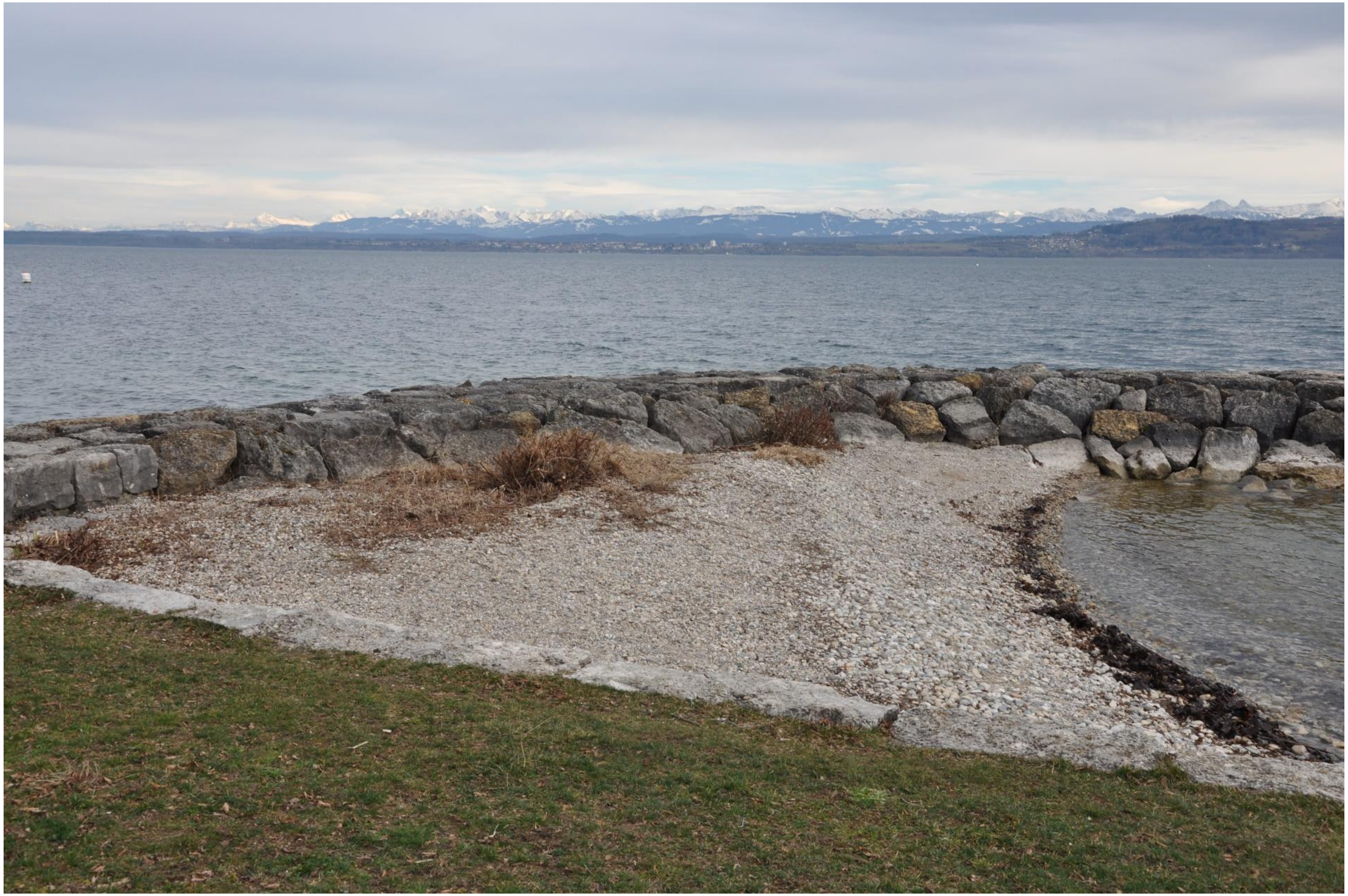

Lake Neuchâtel – Vaumarcus A (beach #25)

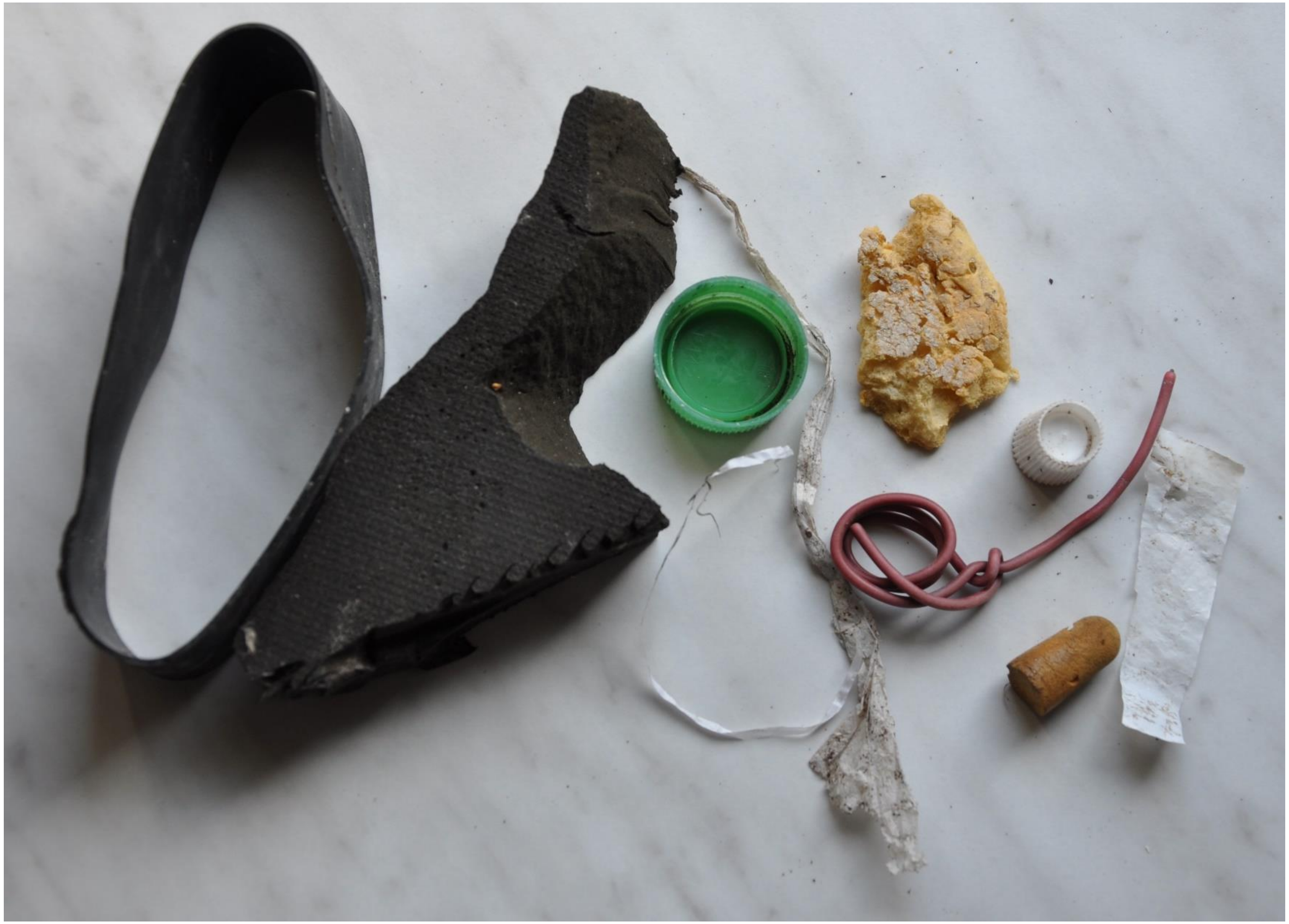

Lake Neuchâtel – Vaumarcus B (beach #26)

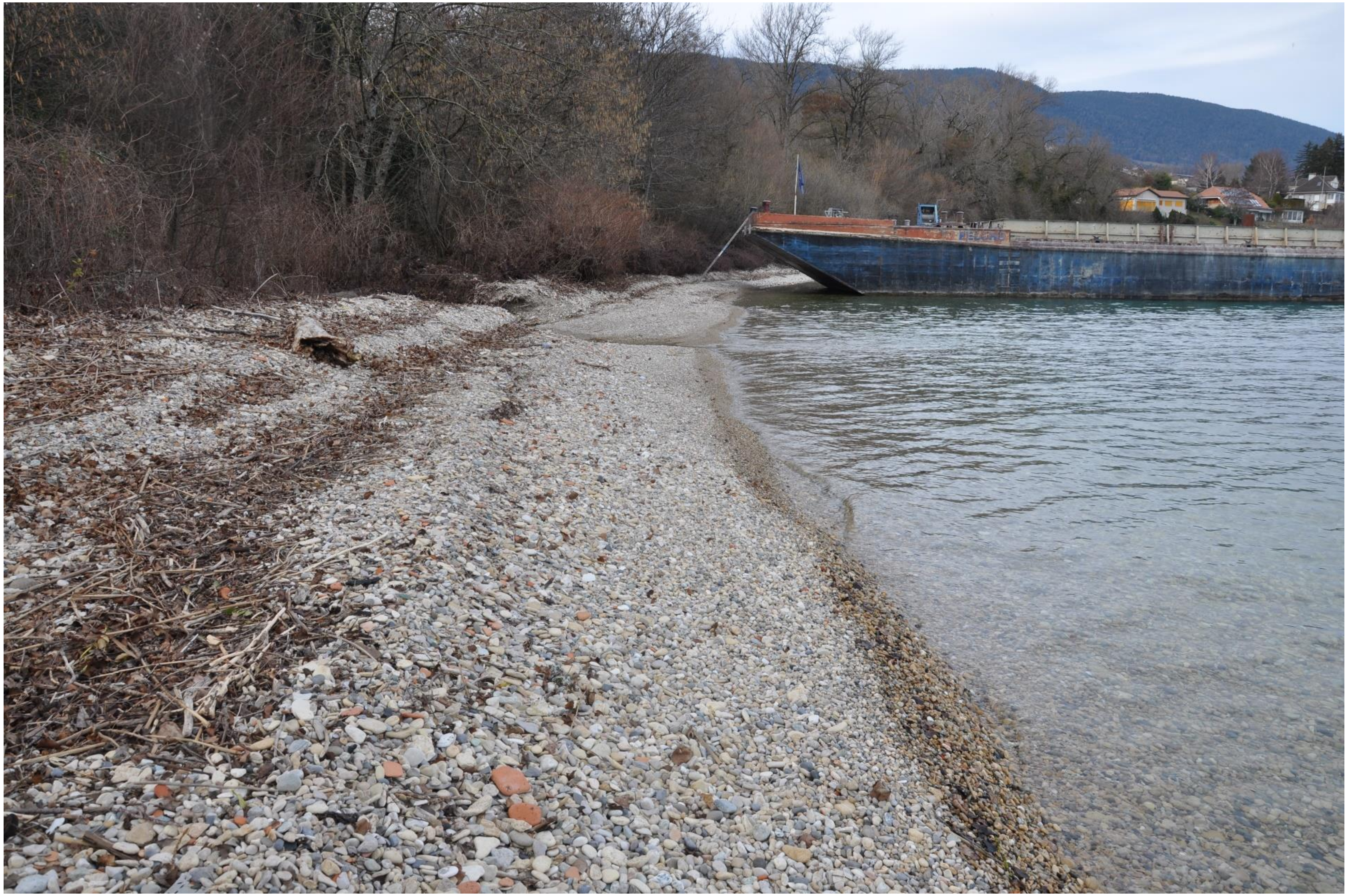

Lake Neuchâtel – Vaumarcus B (beach #26)

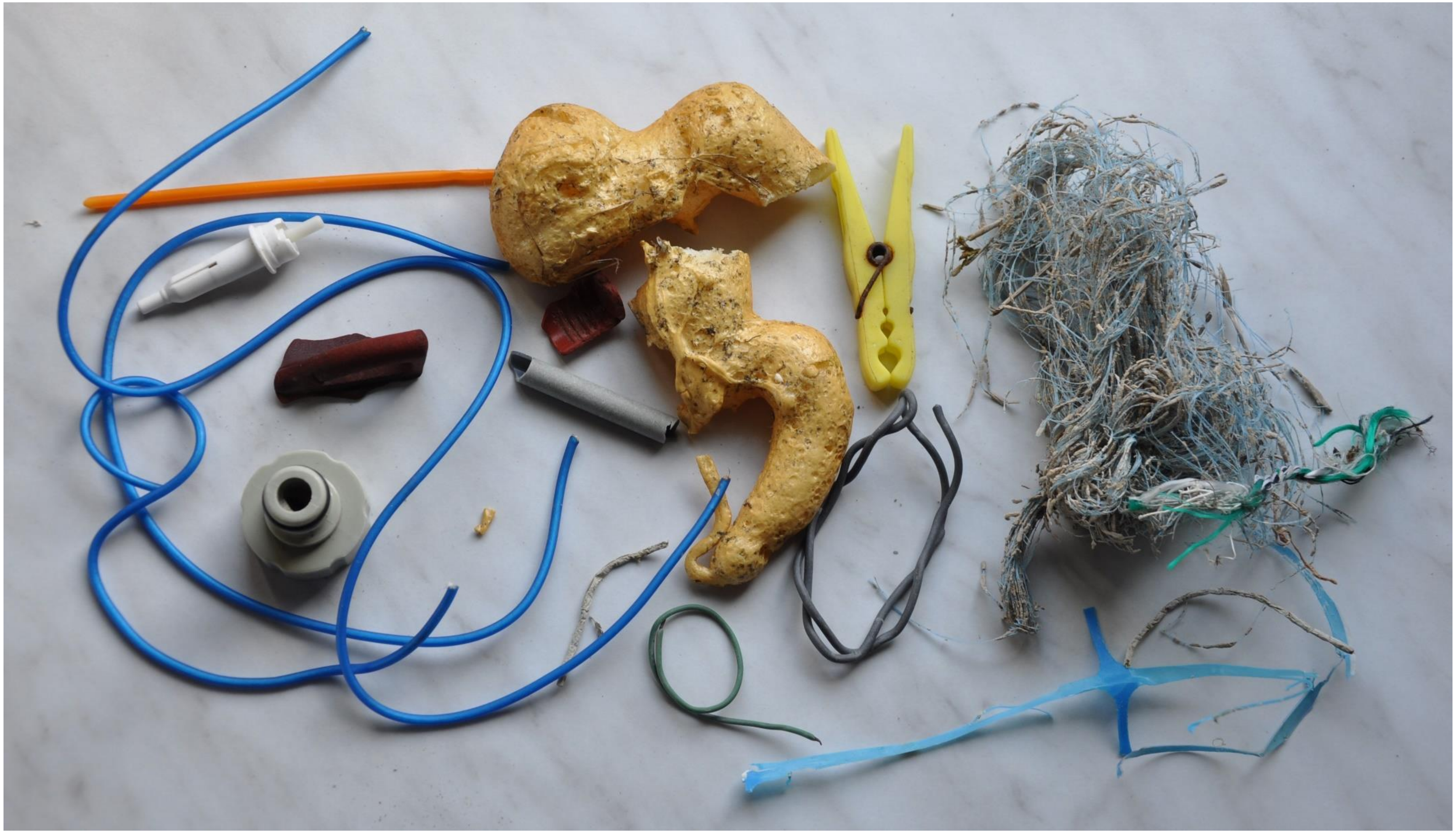

Lake Sempach – Nottwill (beach #27)

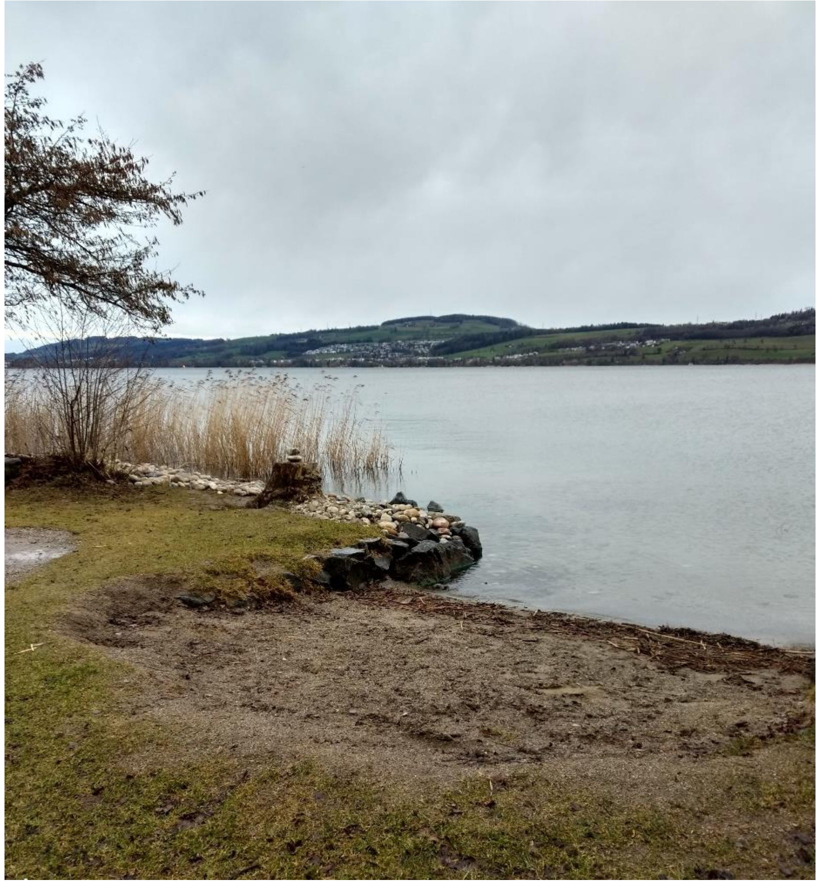

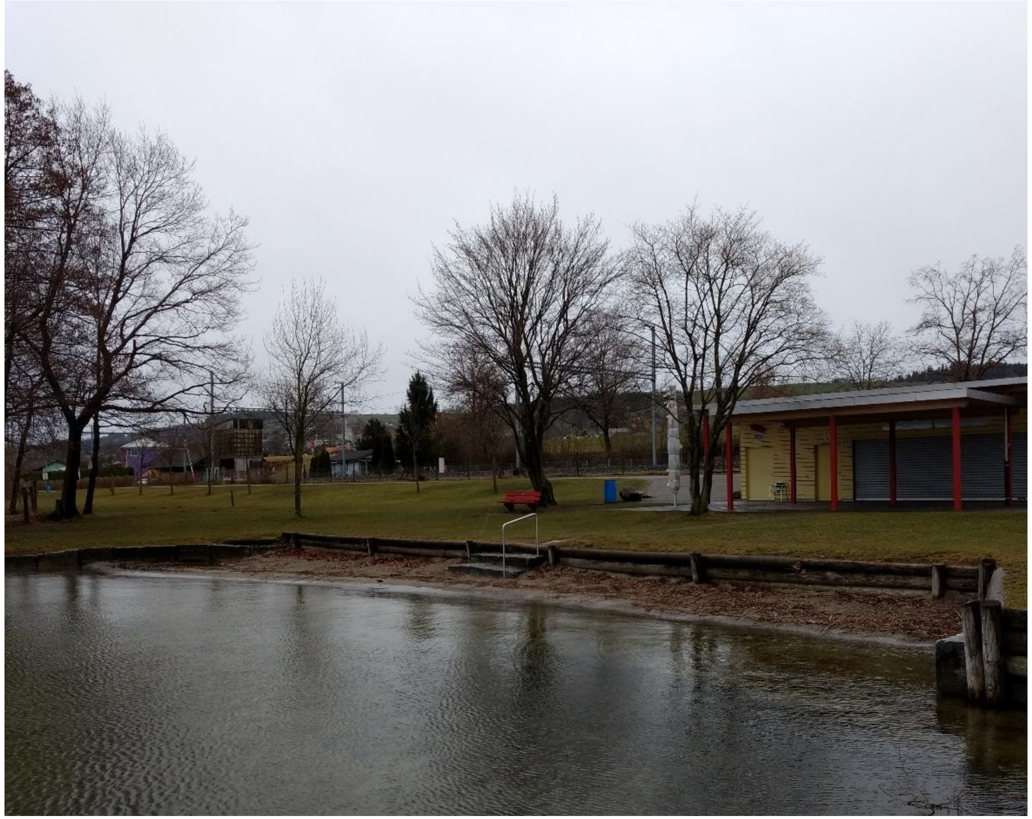

Lake Sempach – Nottwill (beach #27)

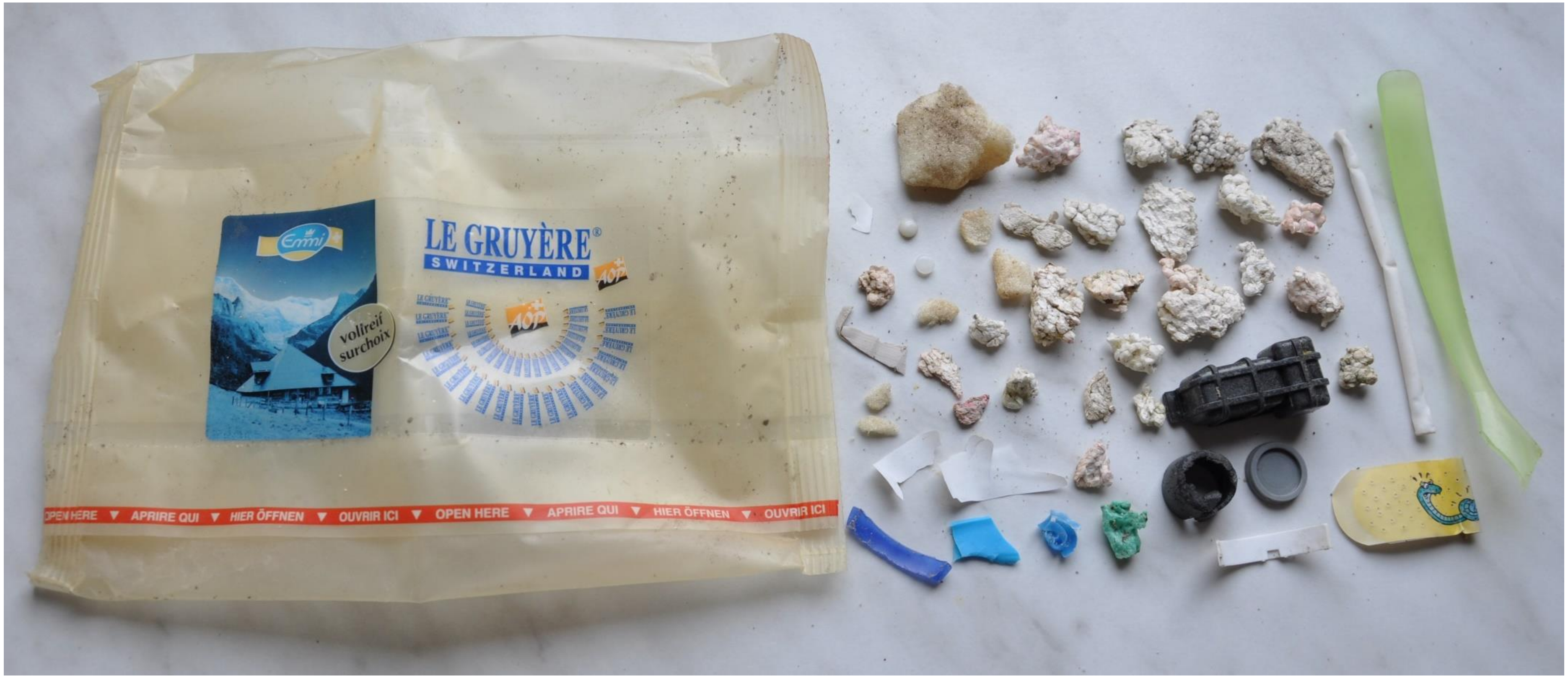

Lake Sempach – Sempach, Restaurant Seeland (beach #28)

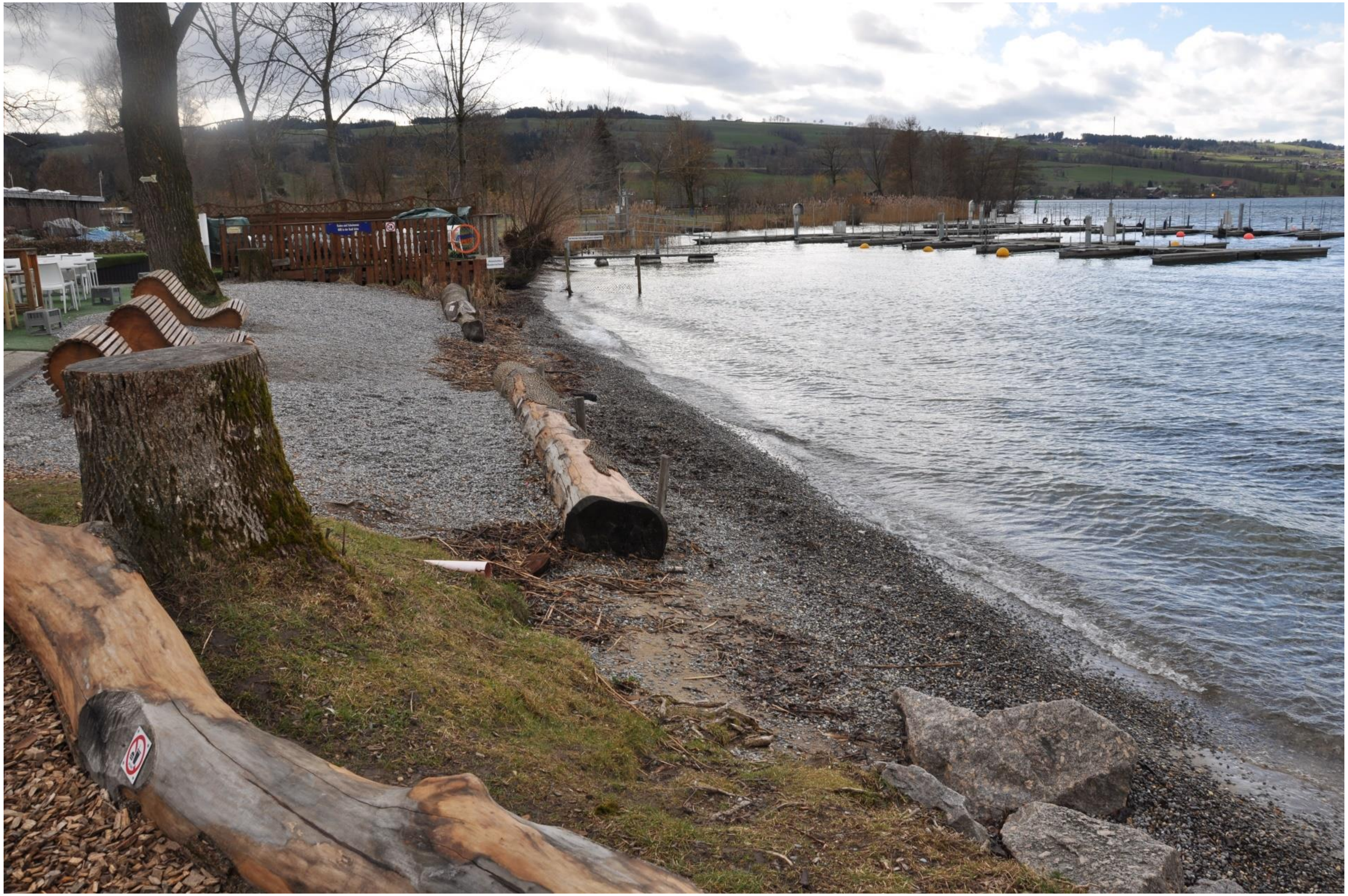

Lake Sempach – Sempach, Restaurant Seeland (beach #28)

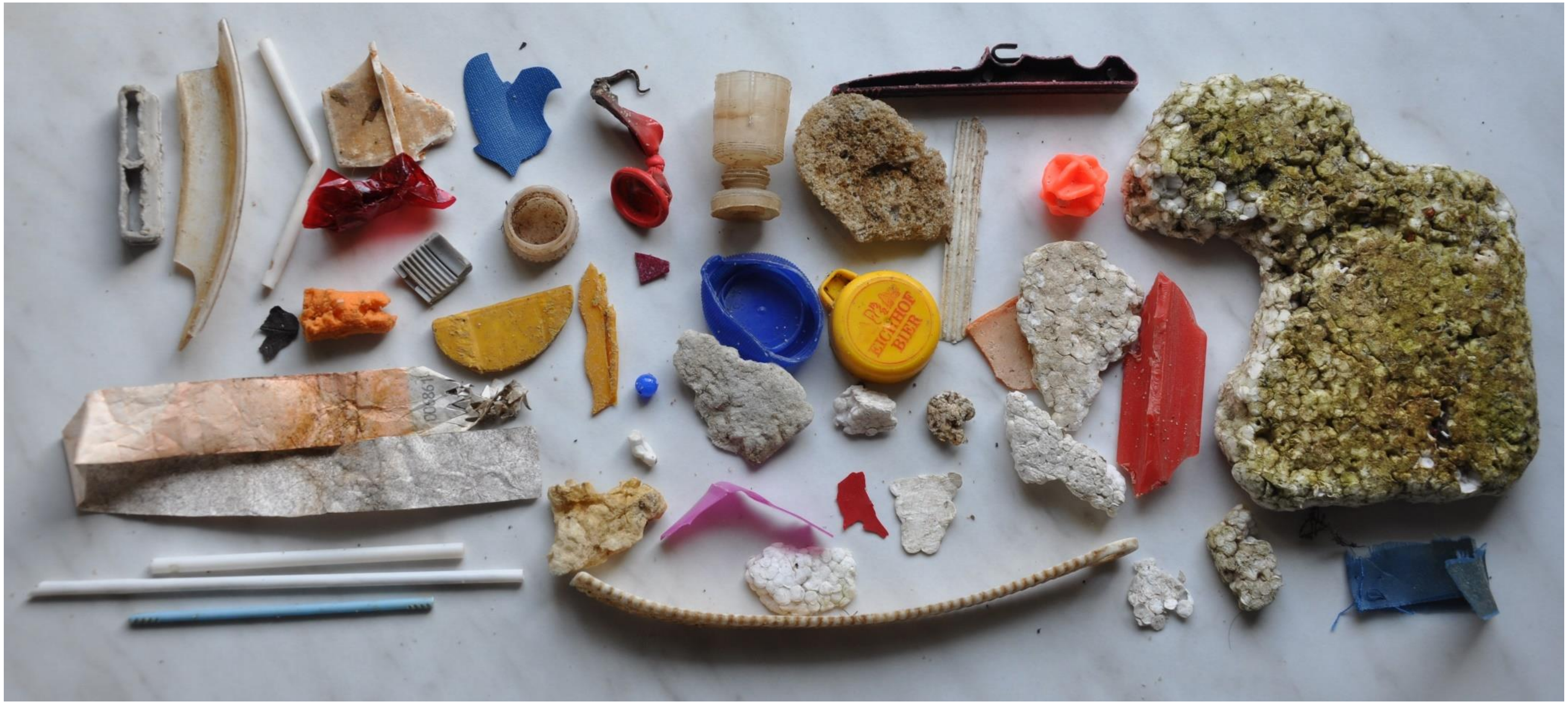

Lake Thun – Gwatt, Kander Delta (beach #29)

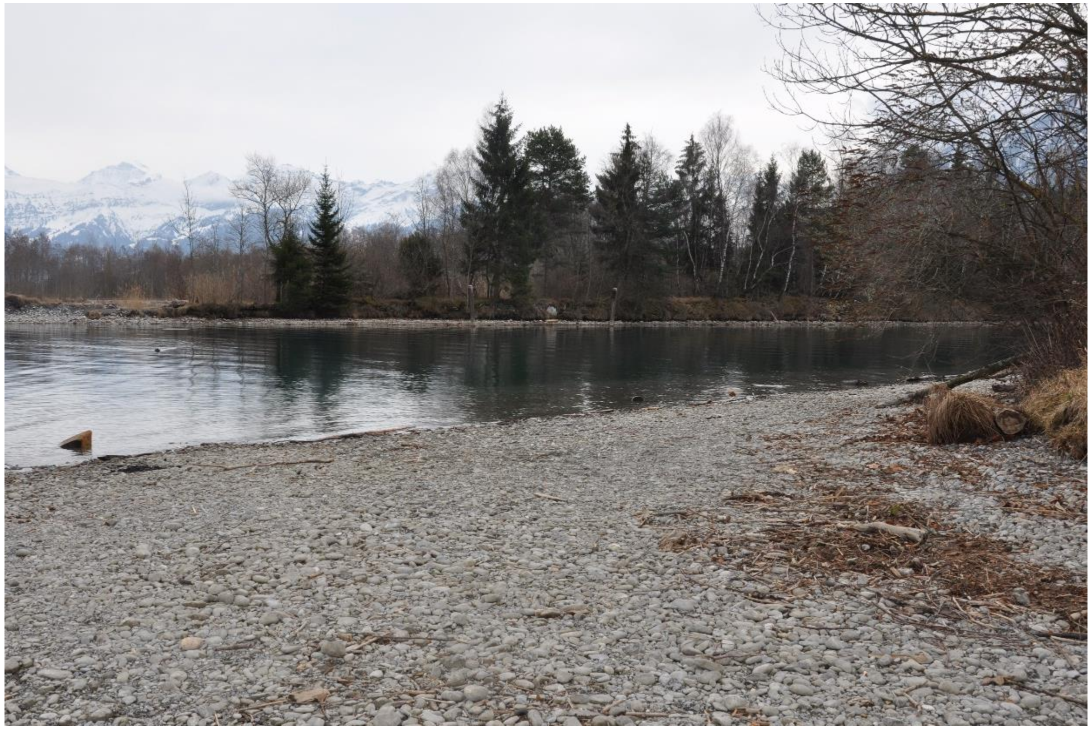

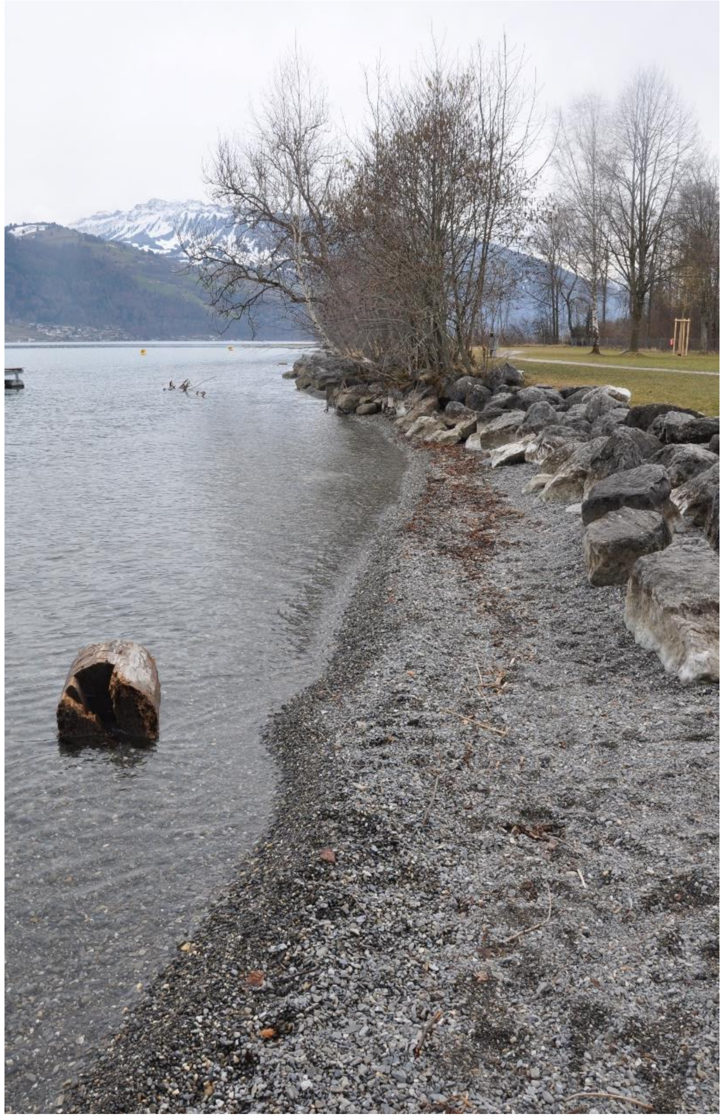

Lake Thun – Gwatt, Kander Delta (beach #29)

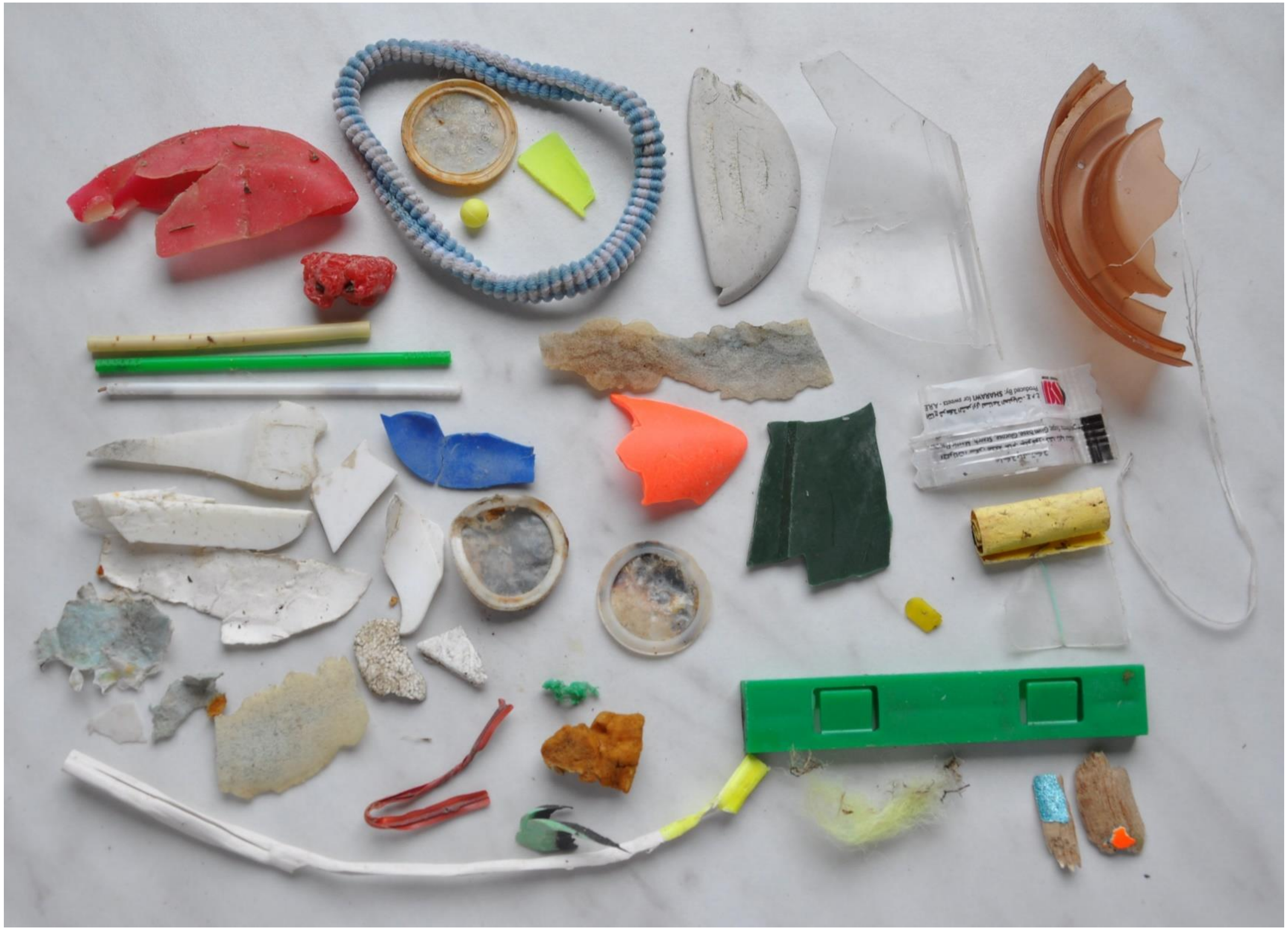

Lake Thun – Interlaken, Lombach mouth (beach #29)

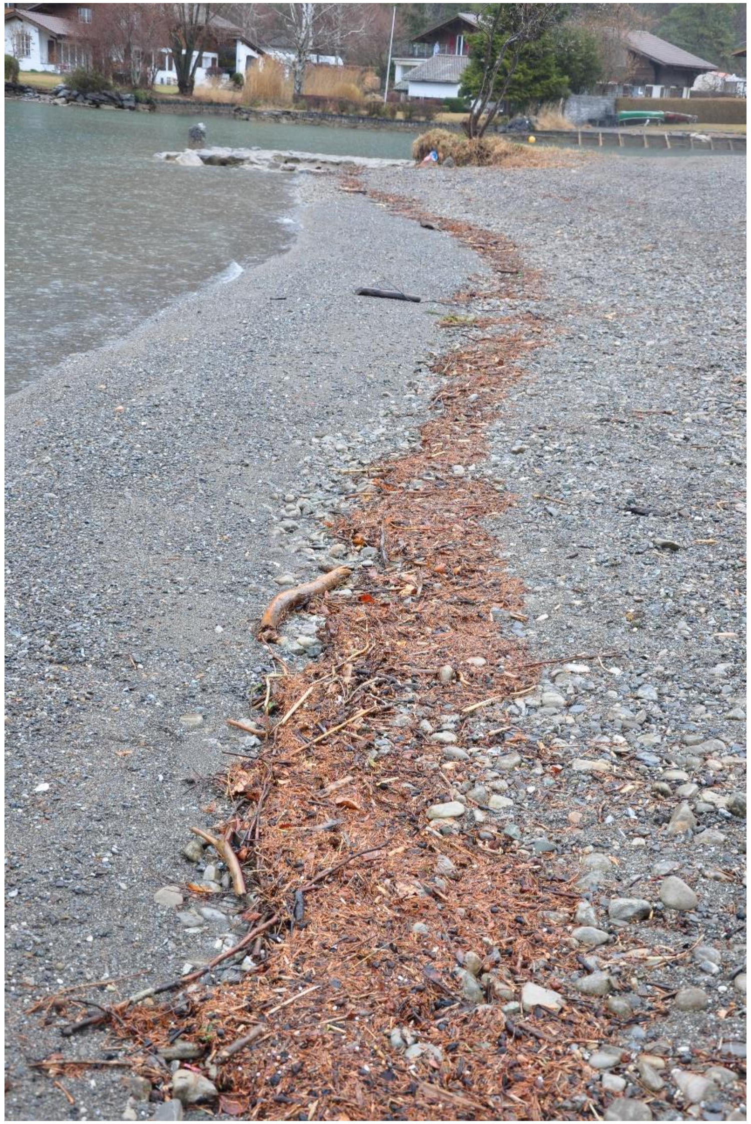

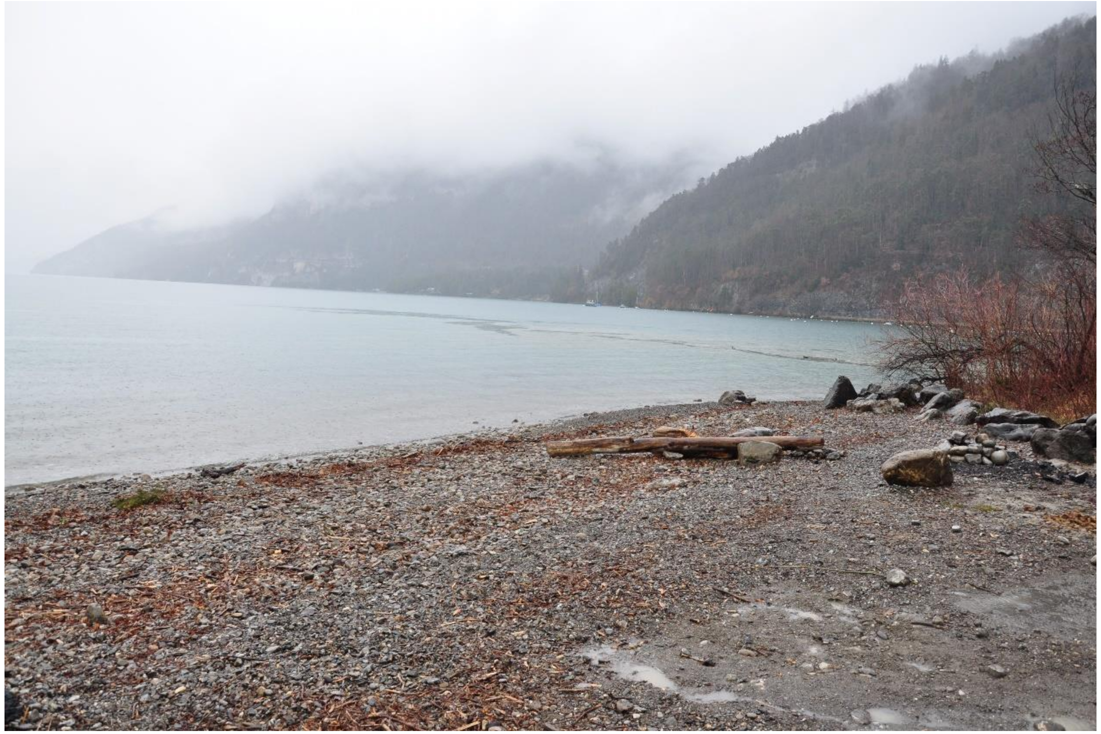

Lake Thun – Interlaken, Lombach mouth (beach #30)

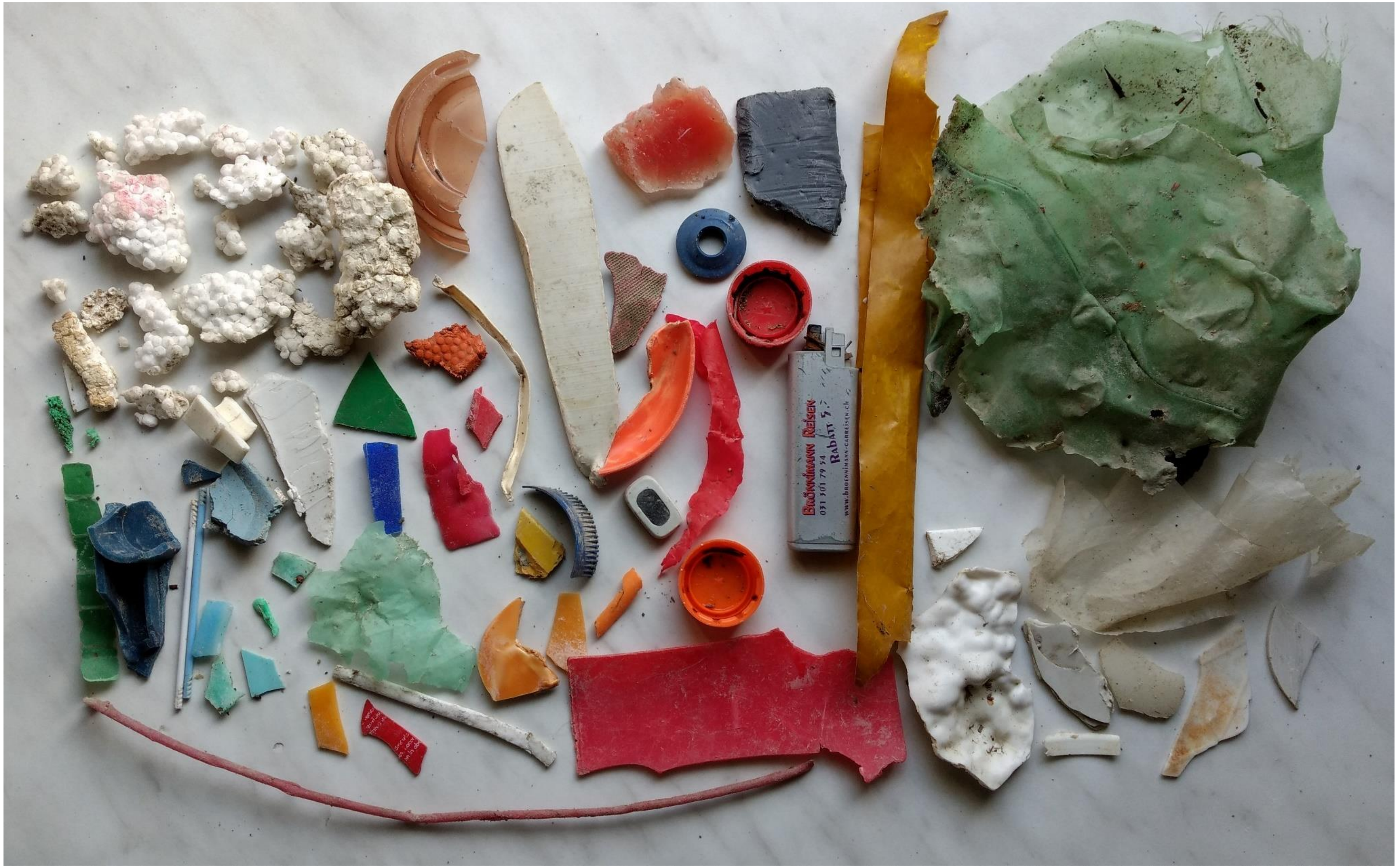

Lake Thun – Leissigen, church (beach #31)

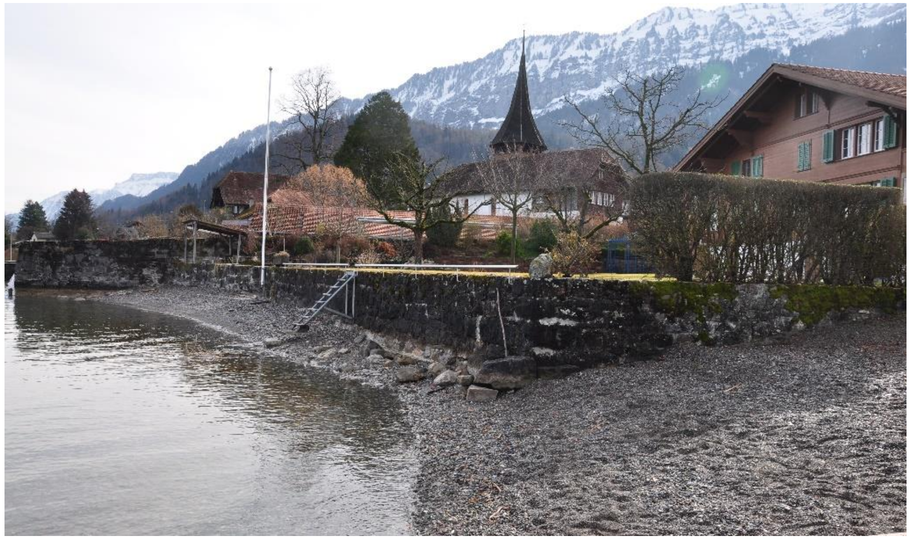

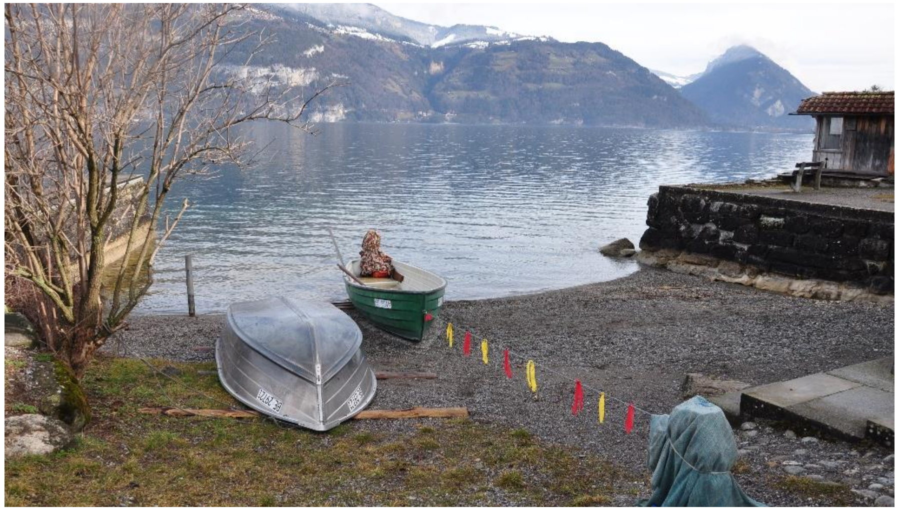

Lake Thun – Leissigen, church (beach #31)

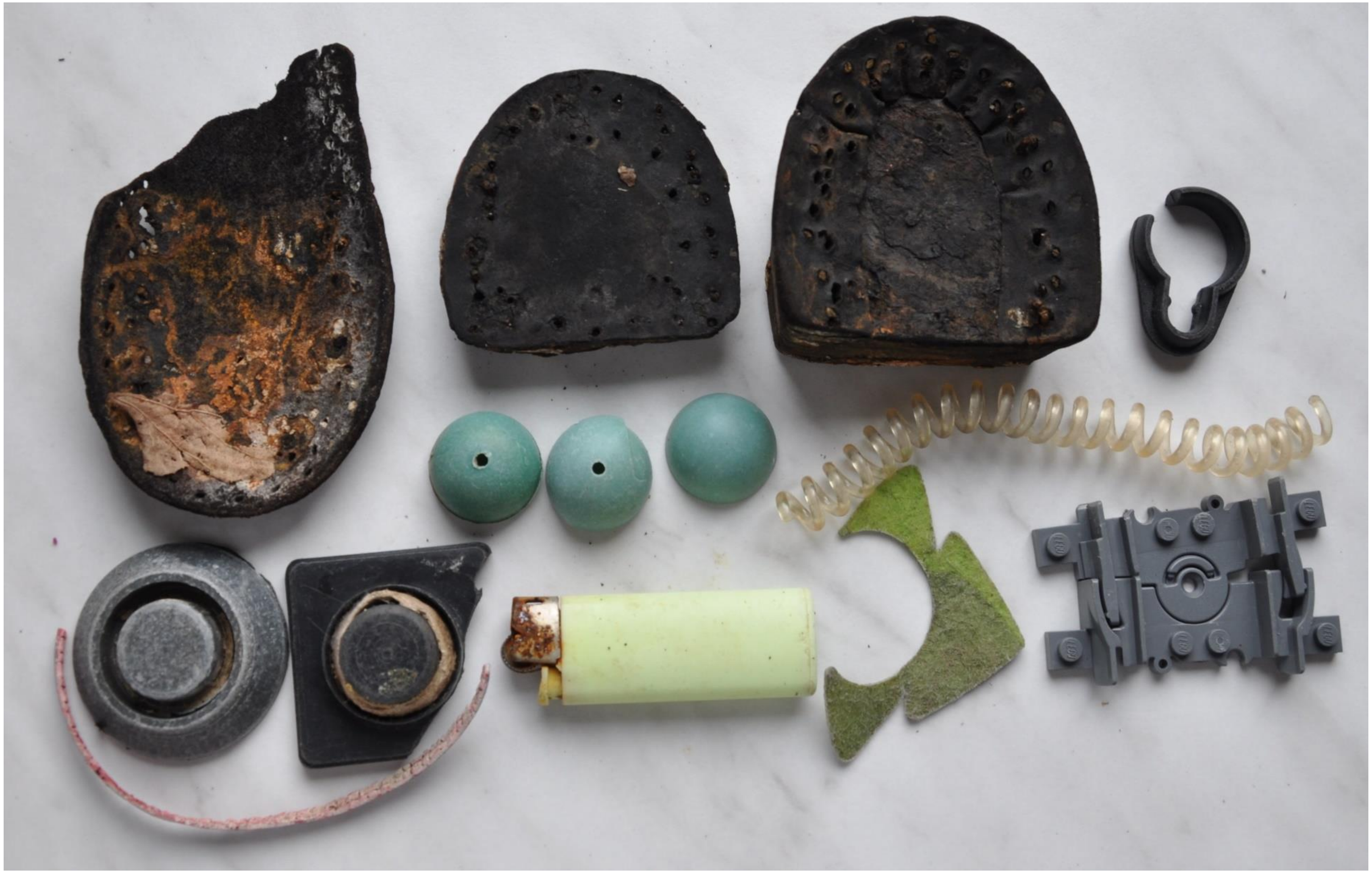

Lake Thun – Leissigen, Schreinerei Heinrich (beach #32)

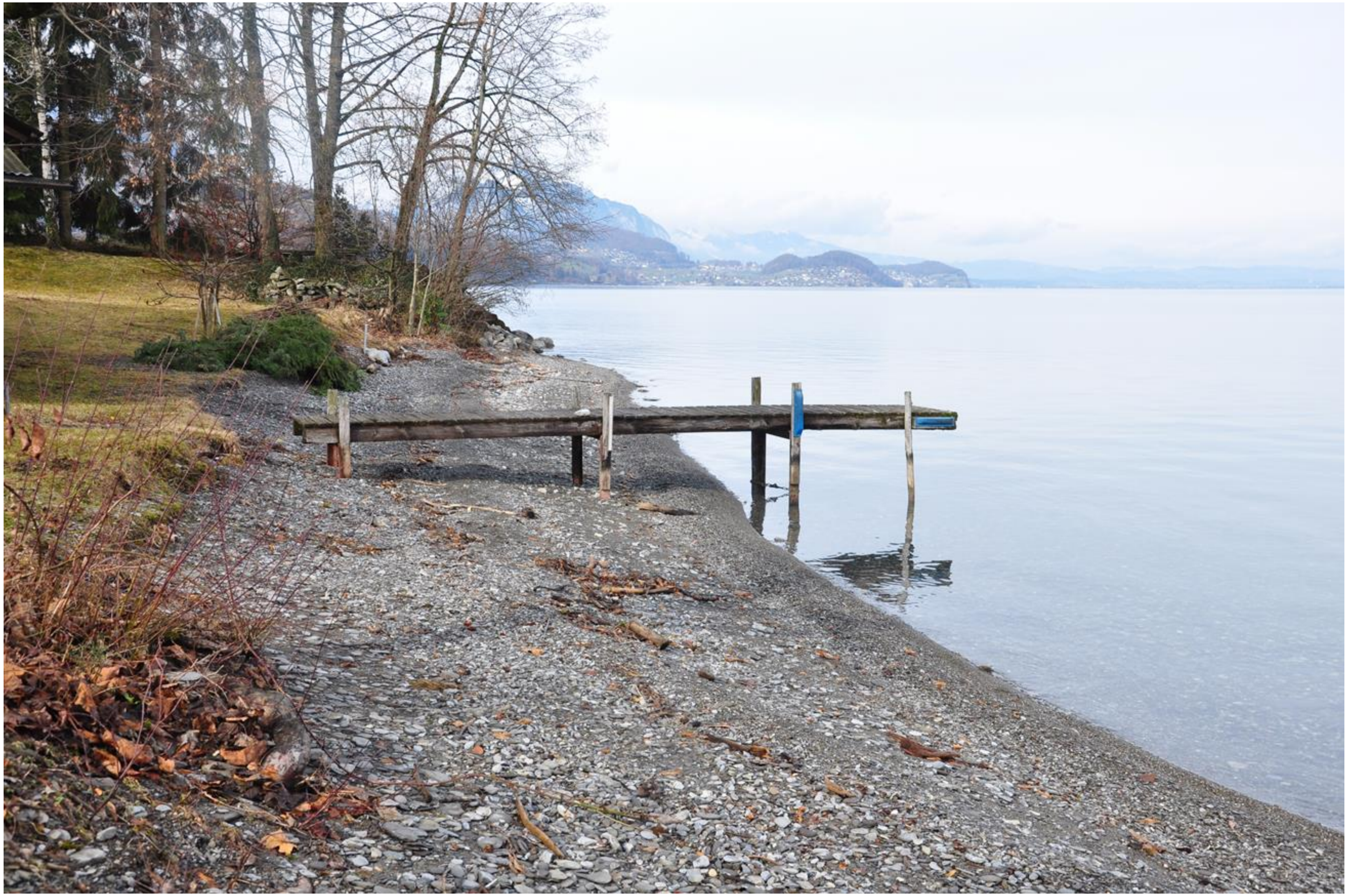

Lake Thun – Leissigen, Schreinerei Heinrich (beach #32)

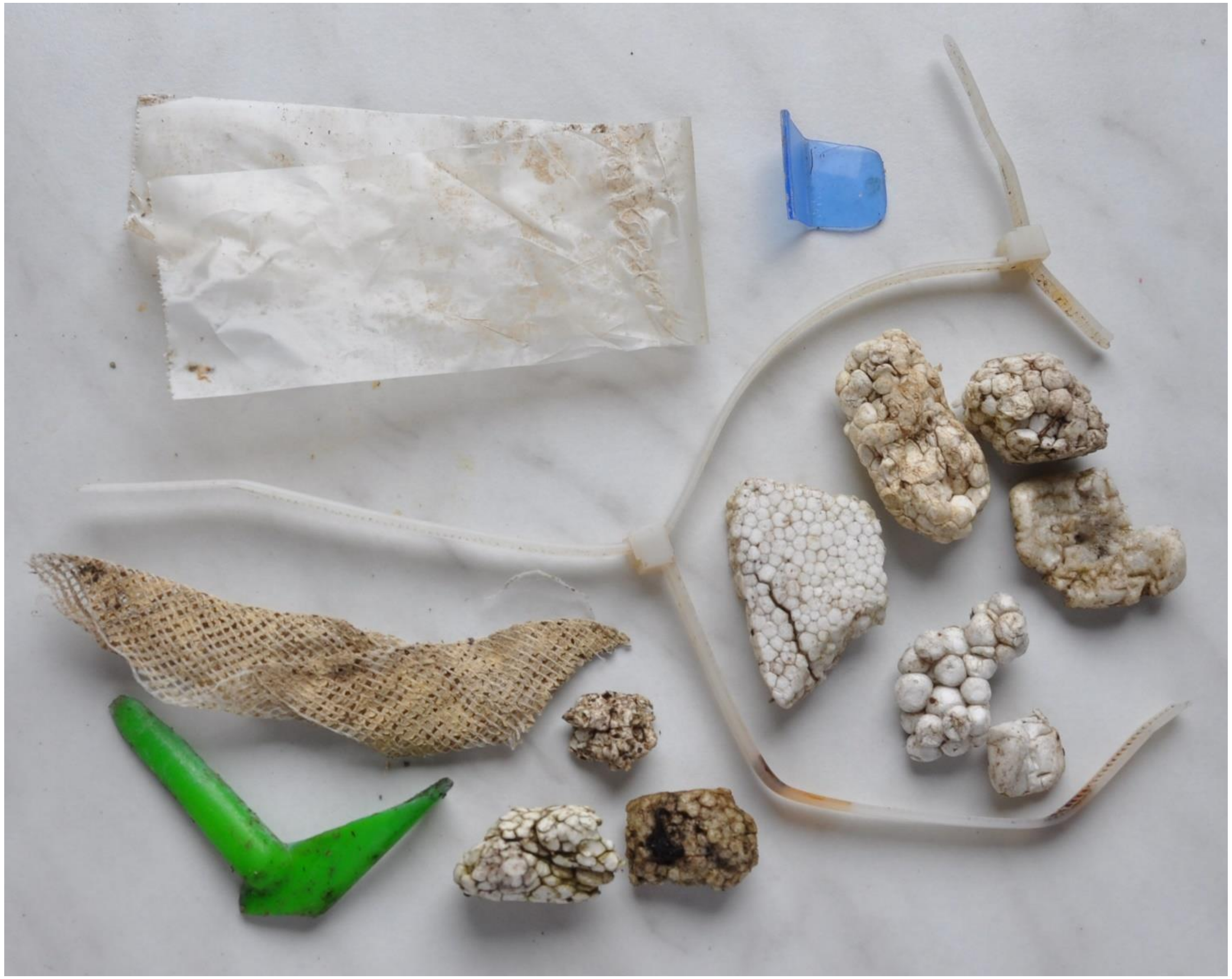

Lake Zug – Hünenberg (beach #33)

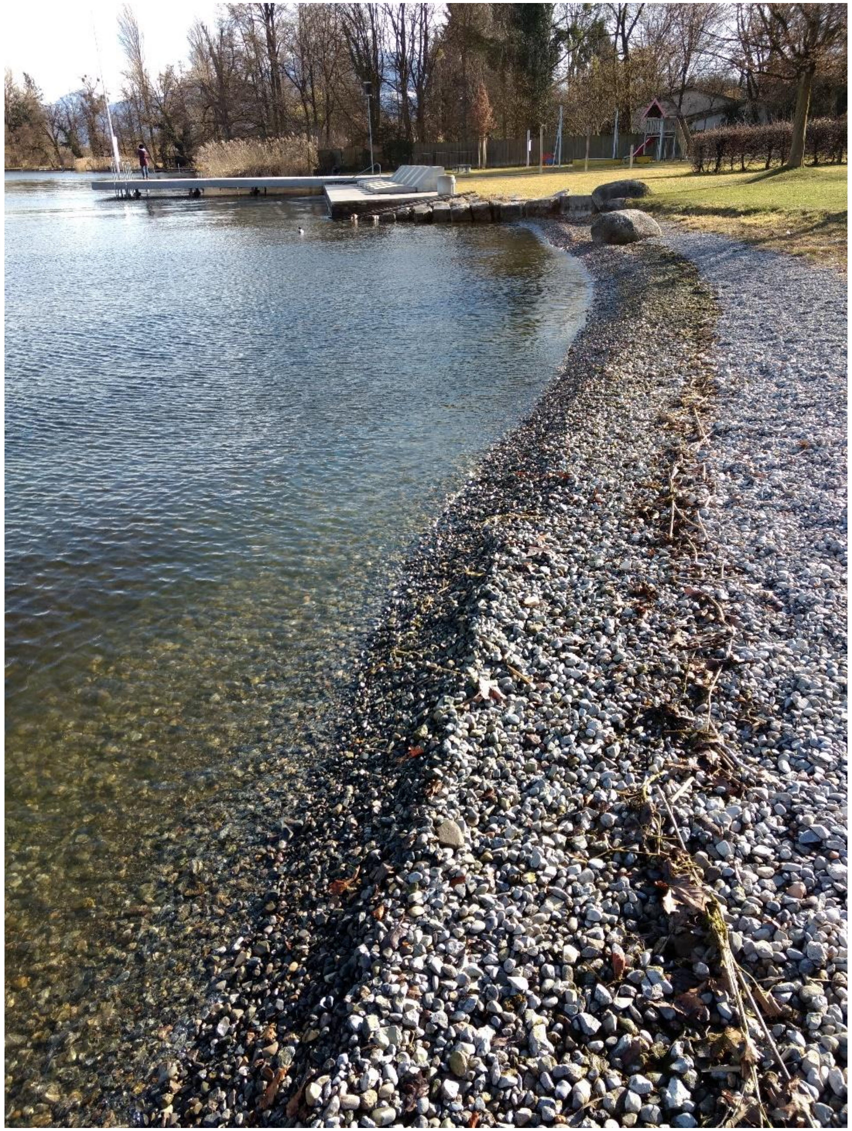

Lake Zug – Hünenberg (beach #33)

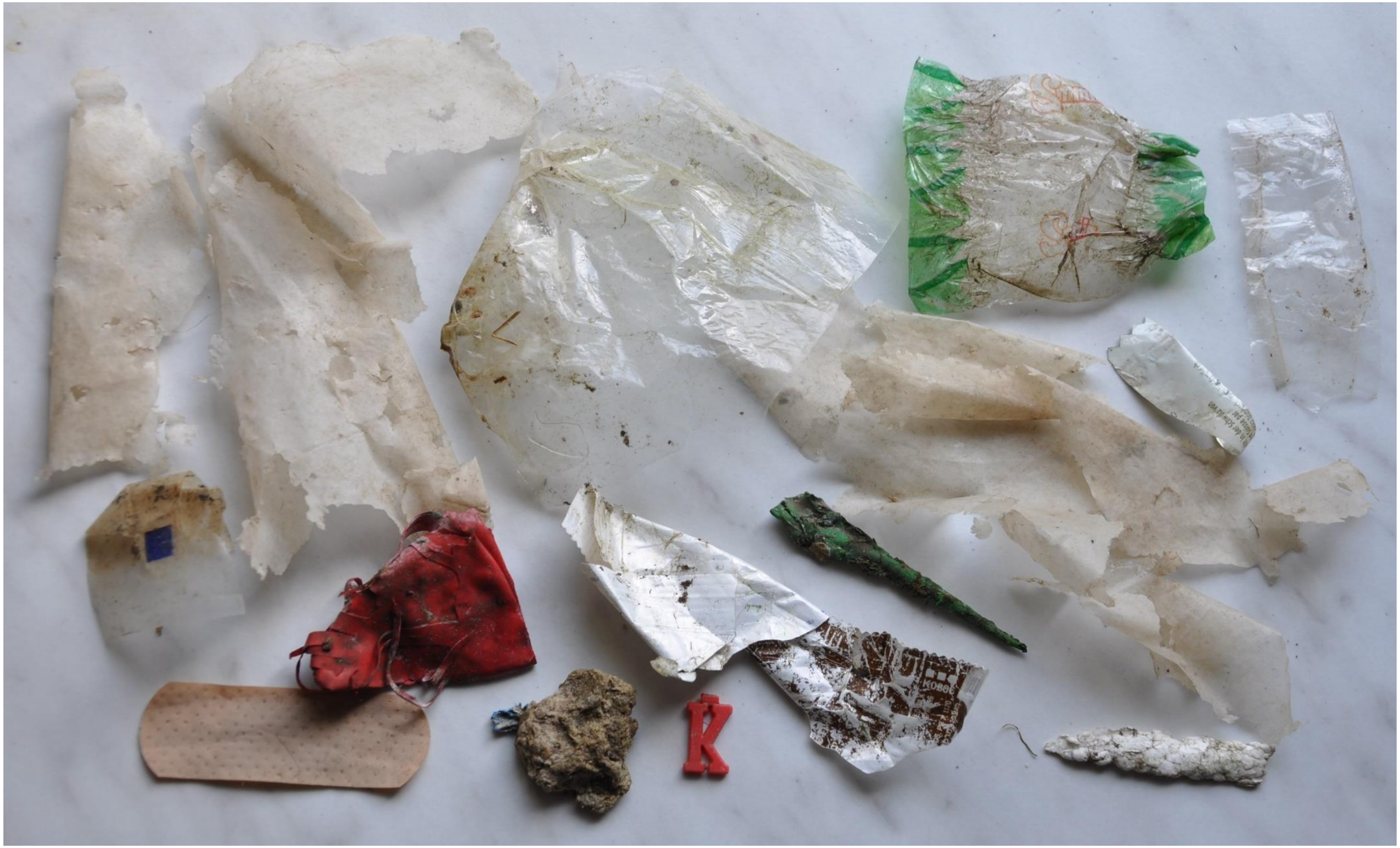

Lake Zurich – Au (beach #34)

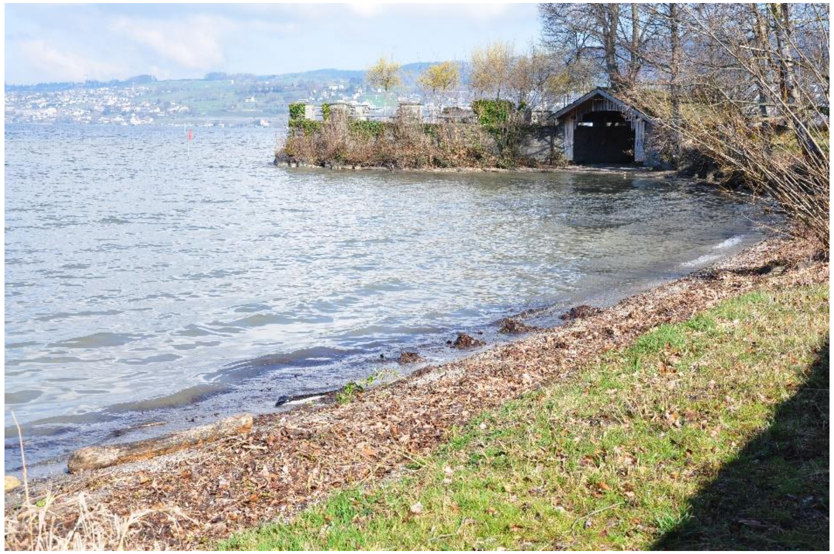

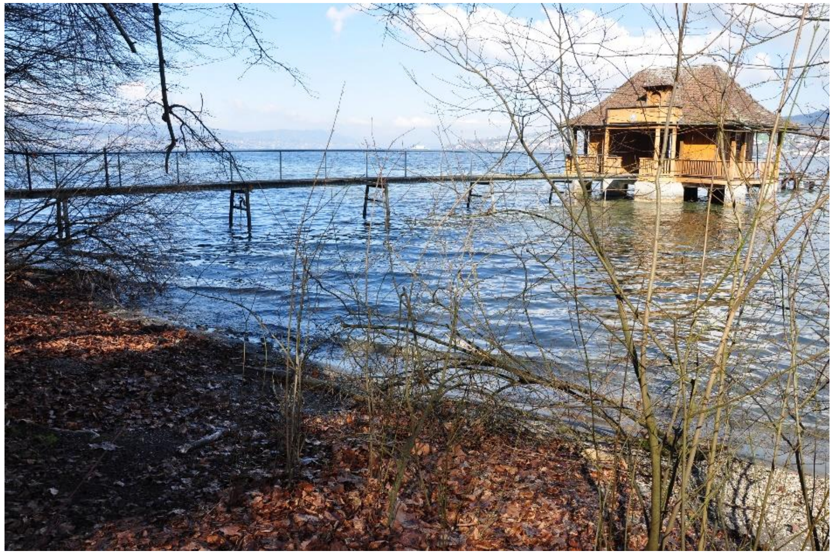

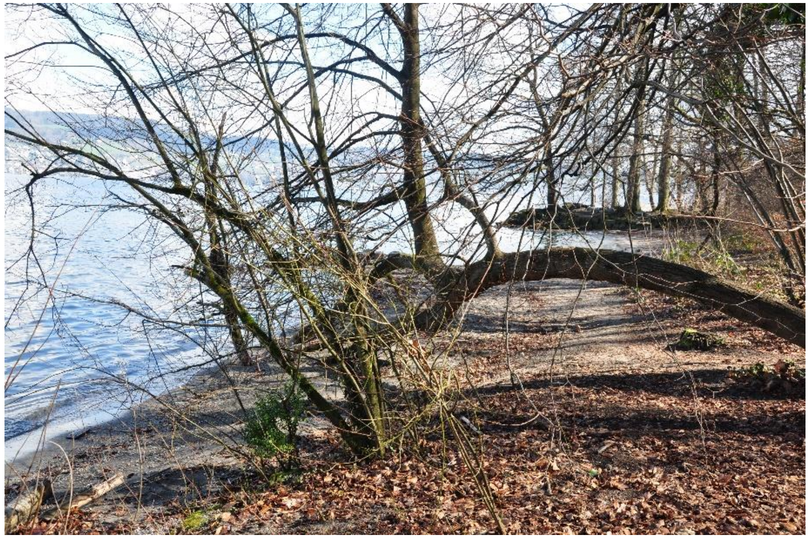

Lake Zurich – Au (beach #34)

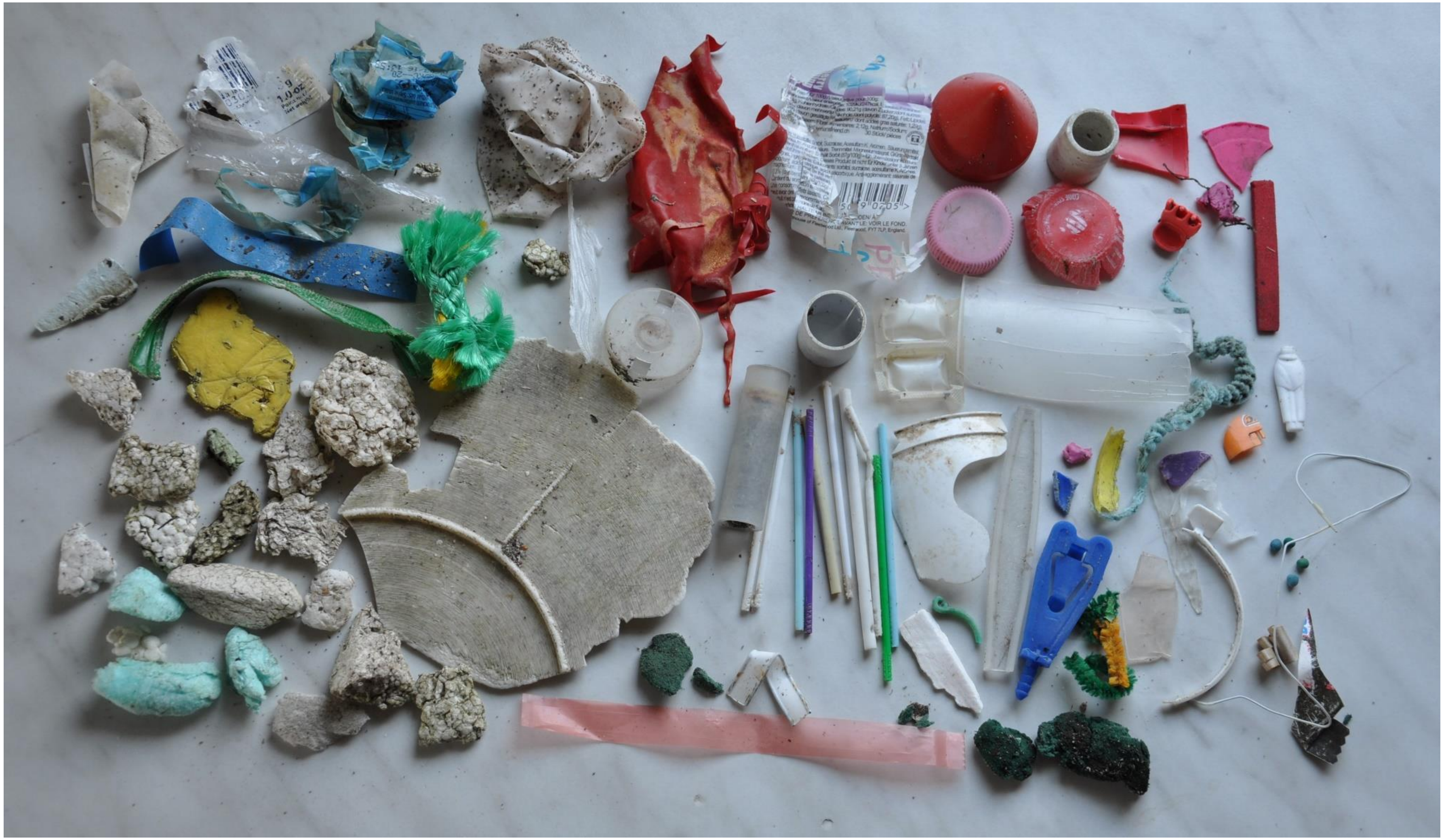

Lake Zurich – Küsnacht (beach #35)

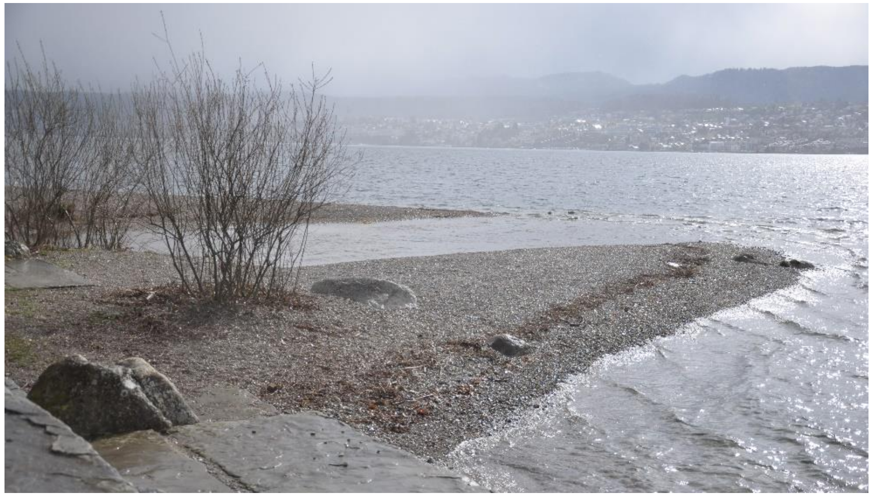

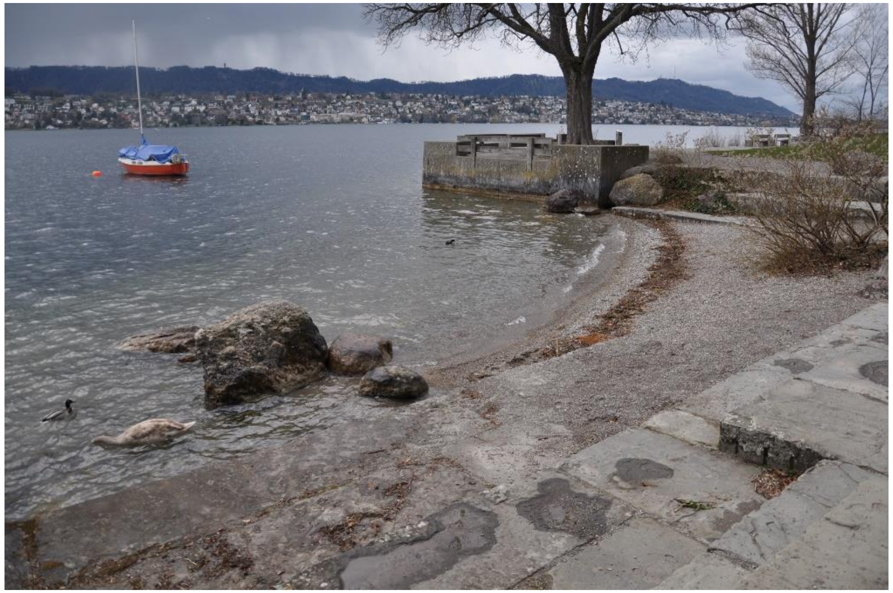

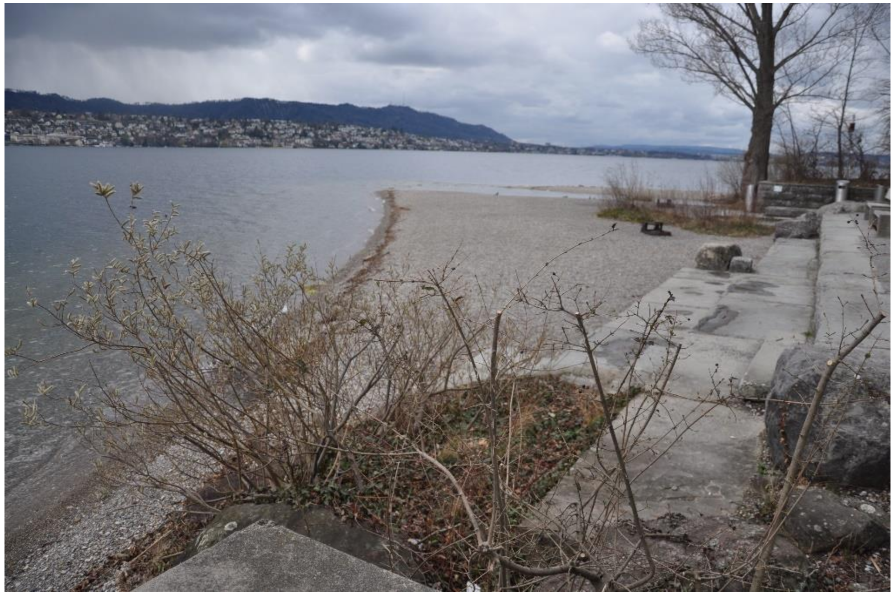

Lake Zurich – Küsnacht (beach #35)

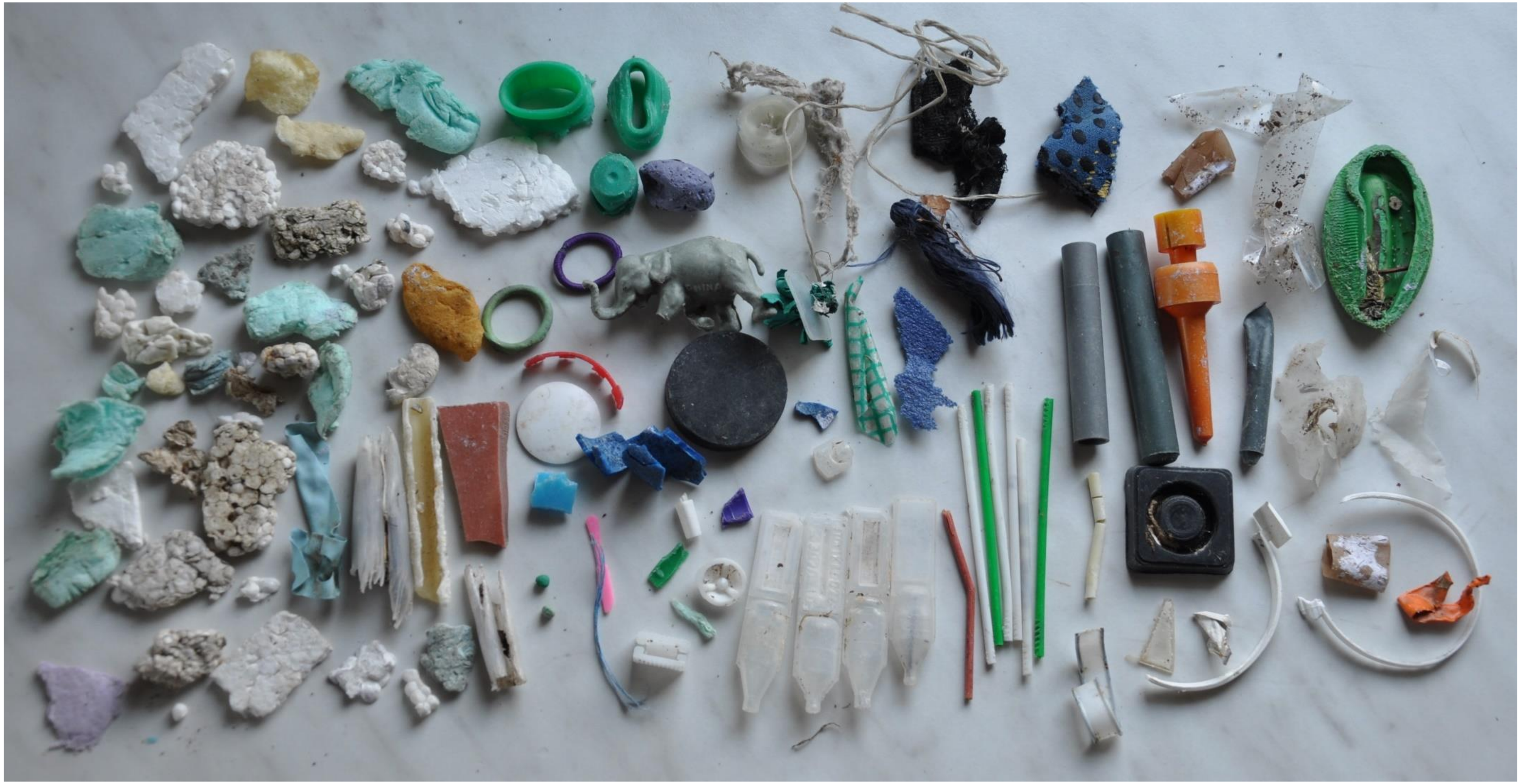

Lake Zurich – Meilen, Strandbad Ländeli (beach #36)

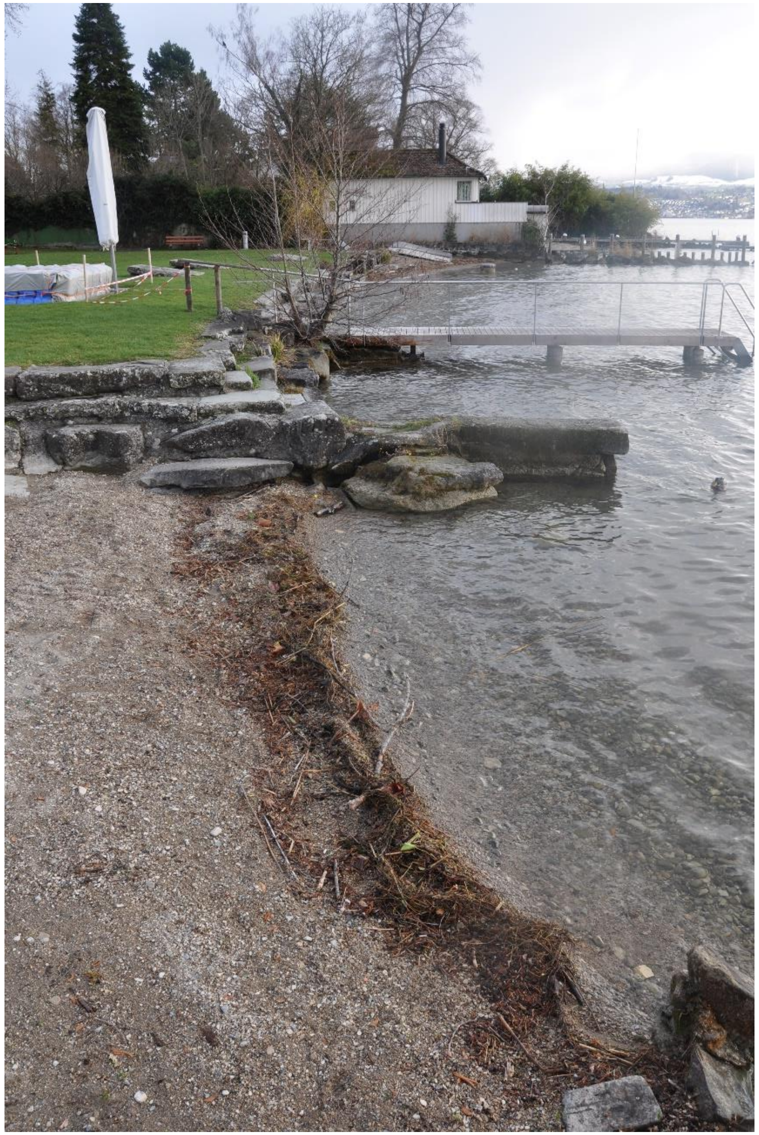

Lake Zurich – Meilen, Strandbad Ländeli (beach #36)

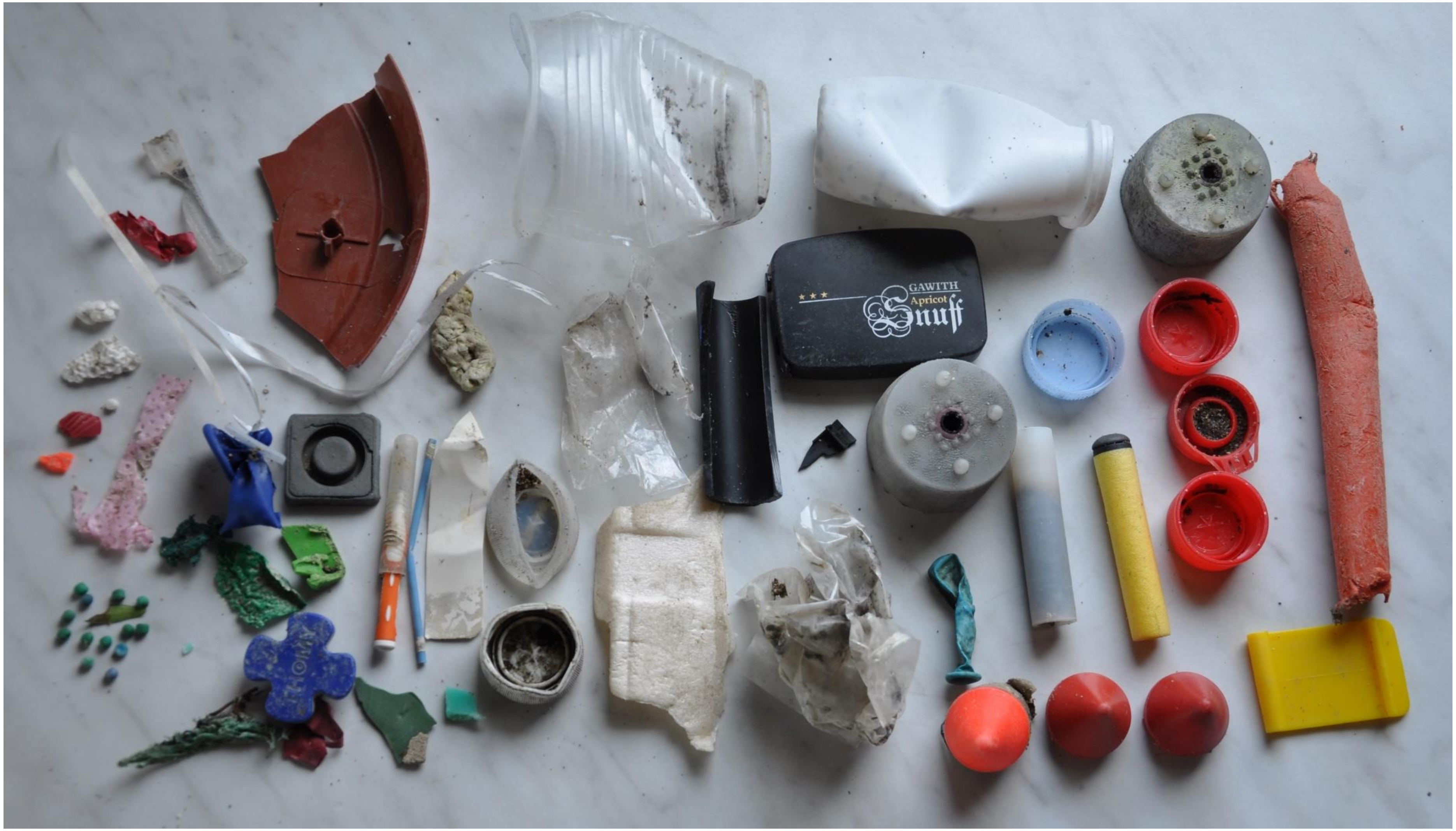

Lake Zurich – Schmerikon (beach #37)

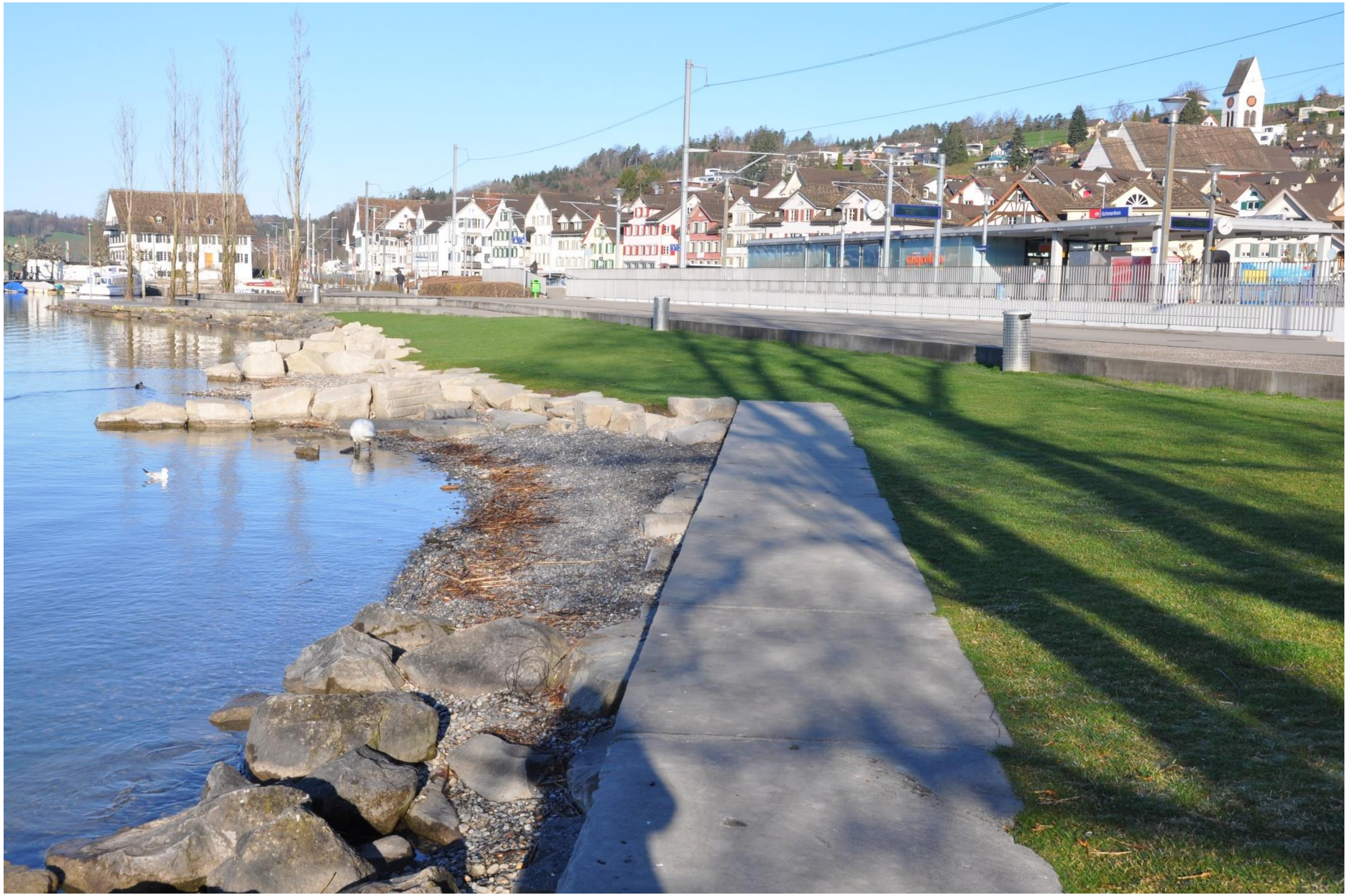

Lake Zurich – Schmerikon (beach #37)

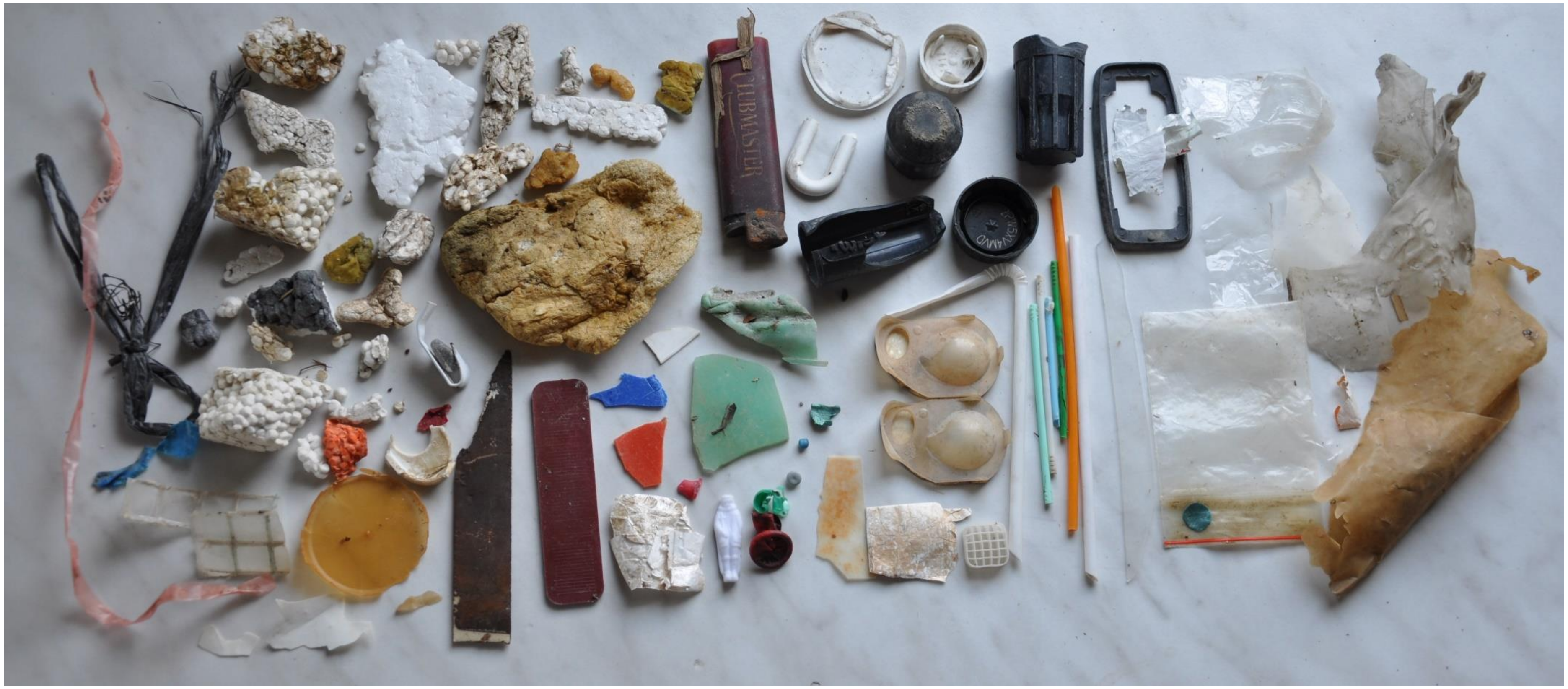

Lake Zurich – Schmerikon, Asbach delta (beach #38)

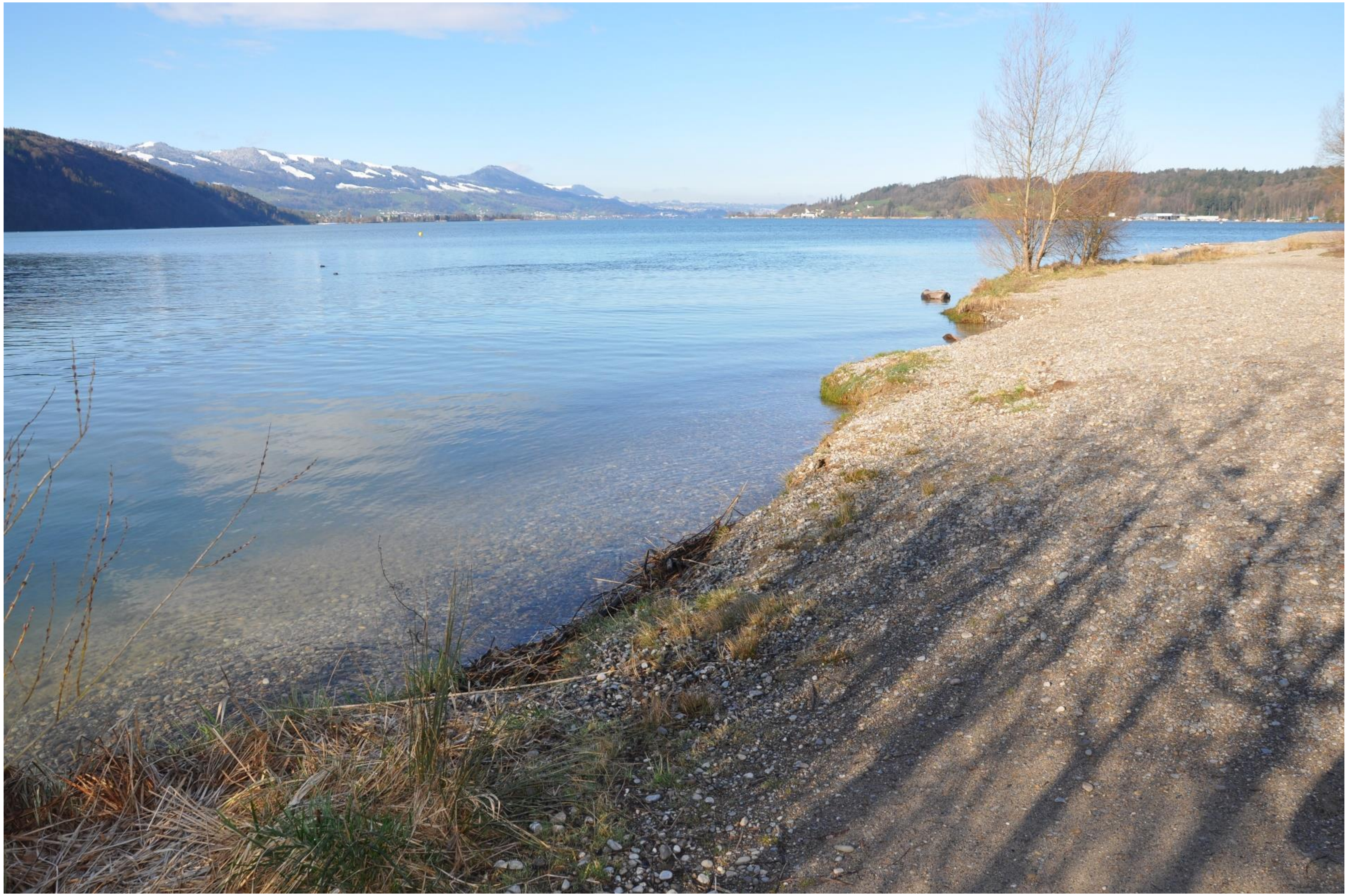

Lake Zurich – Schmerikon, Asbach delta (beach #38)

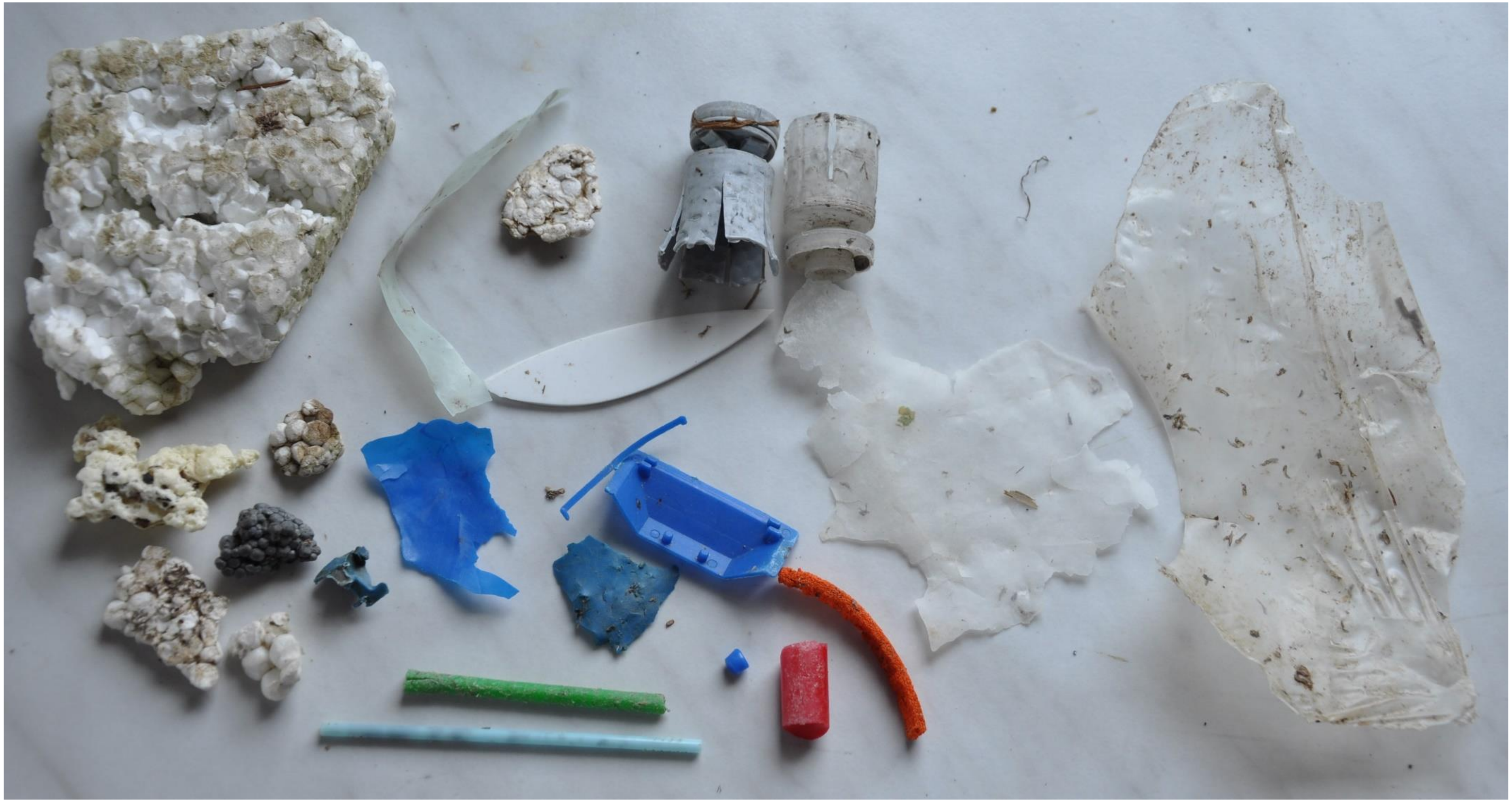

Lake Zurich – Uerikon, Seebad Risi (beach #39)

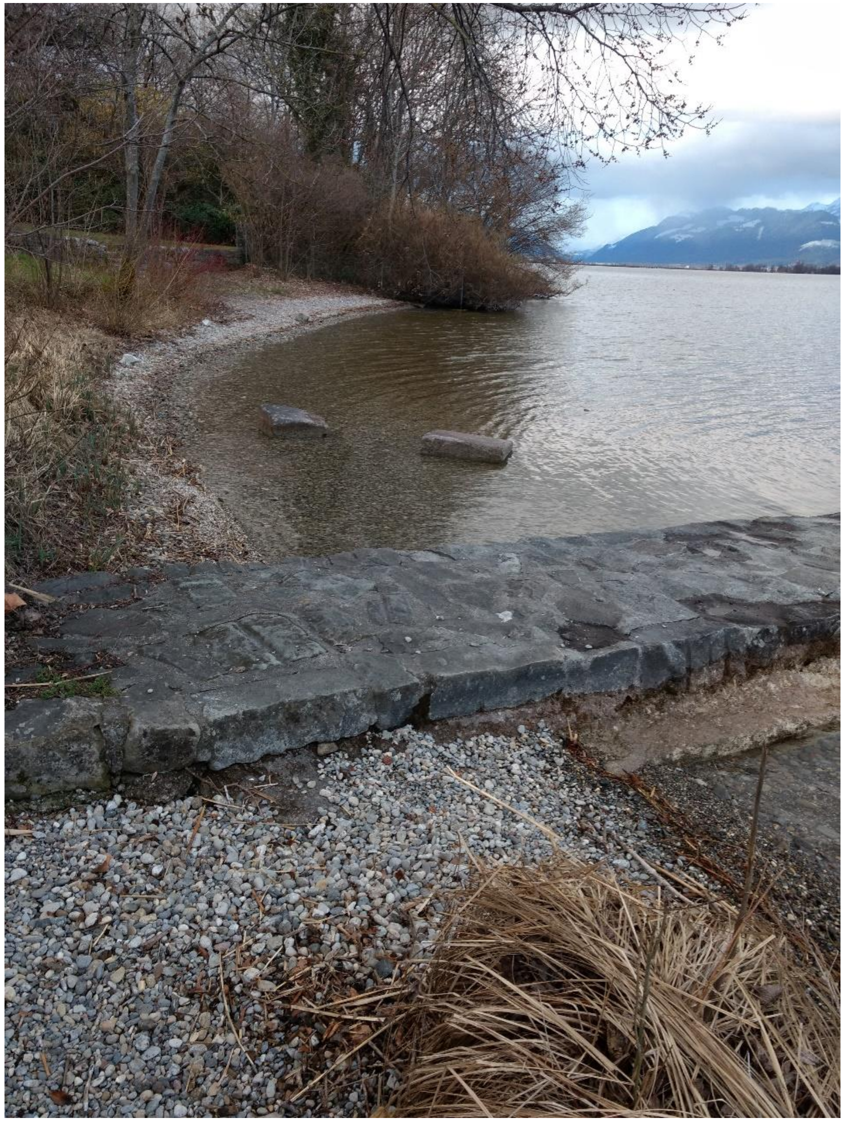

Lake Zurich – Uerikon, Seebad Risi (beach #39)

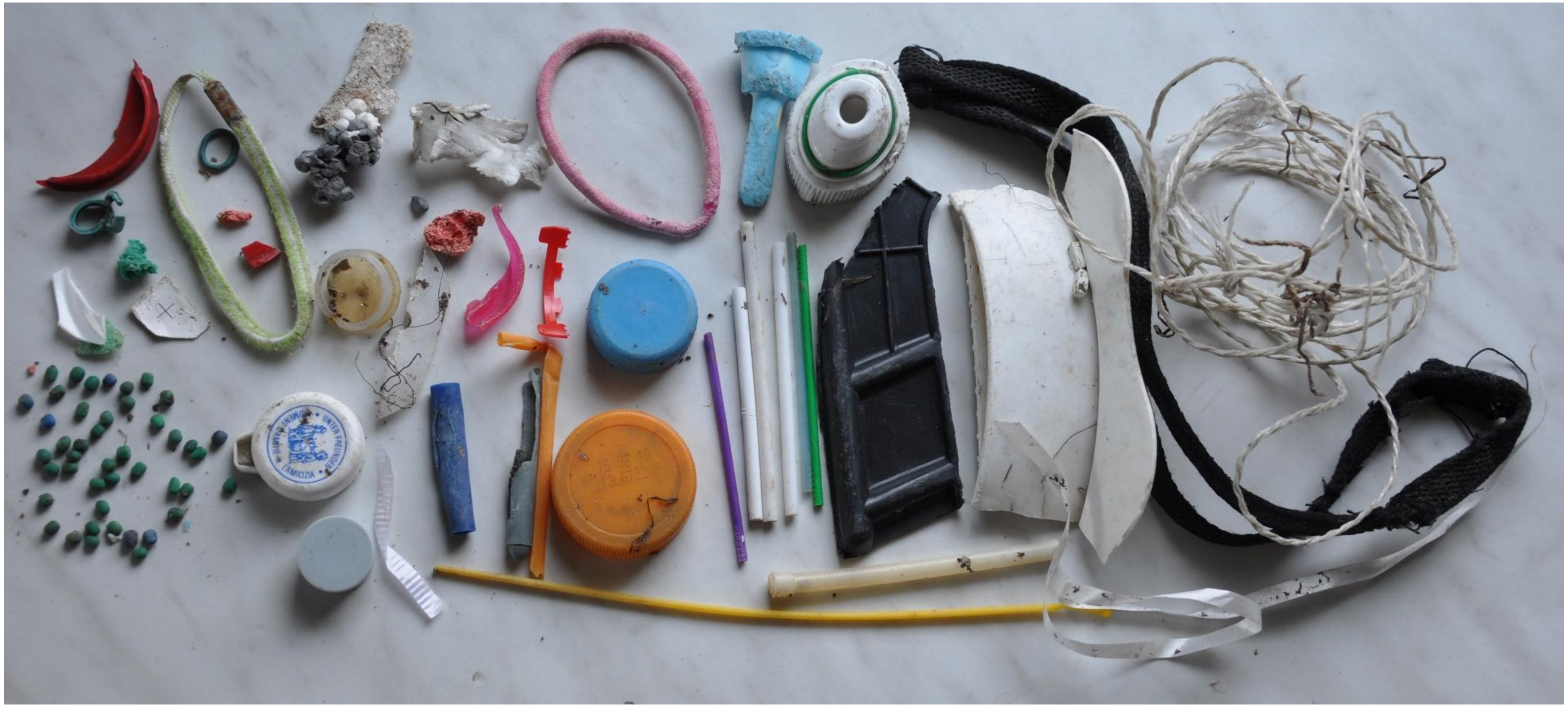

XRF spectra, as counts per second versus energy (keV), and with principal peaks identified for samples:

(a) Lake Constance - Langenargen, Uferpark (beach #13)

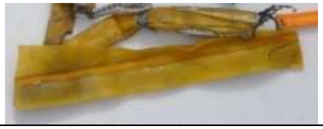

(b) Lake Constance - Kressbronn (beach #11)

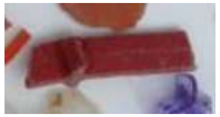

(c) Lake Constance - Lindau, Eichwald Park (beach #15)

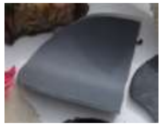

(d) Lake Constance - Staad (beach #19)

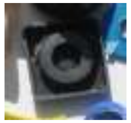

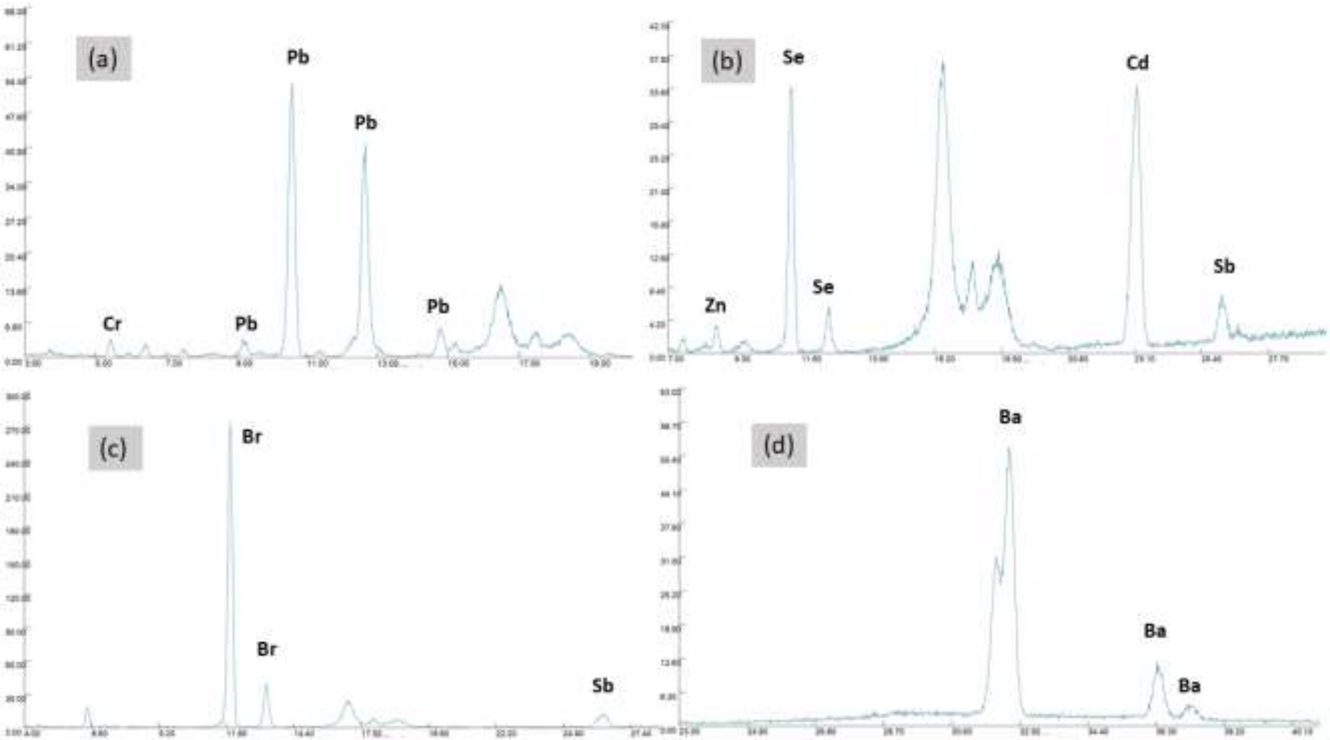

Supplement: Supplementary file 2 — Supplementary file2 (PDF 12830 KB) [file 10661_2021_9384_MOESM2_ESM.pdf]
